# Reaction of allene esters with Selectfluor/TMSX (X = I, Br, Cl) and Selectfluor/NH_4_SCN: Competing oxidative/electrophilic dihalogenation and nucleophilic/conjugate addition

**DOI:** 10.3762/bjoc.11.180

**Published:** 2015-09-16

**Authors:** A Srinivas Reddy, Kenneth K Laali

**Affiliations:** 1 Department of Chemistry, University of North Florida, 1 UNF Drive, Jacksonville, Florida 32224, USA

**Keywords:** allene esters, oxidative electrophilic dihalogenation/conjugate addition, Selectfluor, TMSX and NH_4_SCN

## Abstract

Reaction of benzyl and ethyl allenoates with TMSX (X = I, Br, Cl) and with NH_4_SCN were investigated in MeCN, DMF, and in imidazolium ionic liquids [BMIM][NTf_2_] and [BMIM][PF_6_] as solvent, in the presence and absence of Selectfluor*.* Comparative product analysis studies demonstrate that the ability of Selectflour to promote oxidative/electrophilic dihalogenation/dithiocyanation with TMSX/NH_4_SCN (as observed previously for 1-arylallenes) is diminished in allenoates, most significantly in reactions with TMSCl, and essentially disappearing in reactions with NH_4_SCN, in favor of nucleophilic/conjugate addition. The study underscores the contrasting reactivity patterns in 1-arylallenes and allenoates toward electrophilic and nucleophilic additions in halofunctionalization with TMSX/Selectfluor and thiocyanation reactions with NH_4_SCN/Selectfluor. These competing pathways are influenced by the nature of the anion, allene structure, and the choice of solvent.

## Introduction

Whereas the synthetic potential of Selectfluor^TM^ (F-TEDA-BF_4_) as an efficient, mild, and selective reagent for fluoro-functionalization of organic compounds is widely recognized and exploited [1–10], its ability to act as mediator or catalyst for oxidative functionalization is comparatively less explored [11]. Notable examples of oxidative functionalization by Selectfluor include in situ generation of electrophile equivalents Cl^+^, Br^+^, SCN^+^ and NO_2_^+^ and their reactions with aromatics [12], the bromination of representative alkenes with Selectfluor/KBr [13], and the thiocyanation of representative heteroarenes and ketones with NH_4_SCN [14–15]. Oxidative transformations such as amide to imide mediated by Selectfluor in combination with CuBr have also been shown [16–17].

In an earlier study, we reported on the potential of Selectfluor to act as mediator and oxidant in the reaction of 1-arylallenes with TMSX (X = Cl, Br, I, NCS) and with NH_4_SCN to bring about dihalogenation and dithiocyanation [18]. The predominant formation of dihaloalkenes and dithiocyanoalkenes observed in these reactions were rationalized by an electrophilic attack of “X^+^” or “SCN^+^” at the central carbon of the allenyl moiety to form incipient allyl cations which on subsequent quenching with X^−^ or SCN^−^ furnished the 2,3-adducts as major products. The 1,2-addition products were only observed with TMSCl. The reactions were carried out in MeCN and in imidazolium ionic liquids (ILs) as solvent in which Selectfluor is soluble.

Previous studies have shown that the major products arising from the reaction of 2,3-allenoates with MX/HX are hydrohalogenated compounds [19–21]. Similarly, reactions with NuH lead to Michael-type nucleophilic additions, but in the presence of phosphane catalysts an umpolung addition takes place, whereby the nucleophilic addition occurs inversely at the beta-gamma double bond [22–23]. Vinyl azides have been prepared by hydroazidation of allenyl esters through a Michael-type addition with high regio- and stereoselectivity [24].

Inspired by these results we focused our attention in the present study on the reaction of allenoates with TMSX with the aim to determine the extent by which Selectfluor could influence the electrophilic versus nucleophilic addition manifolds.

## Results and Discussion

**Scheme 1:**
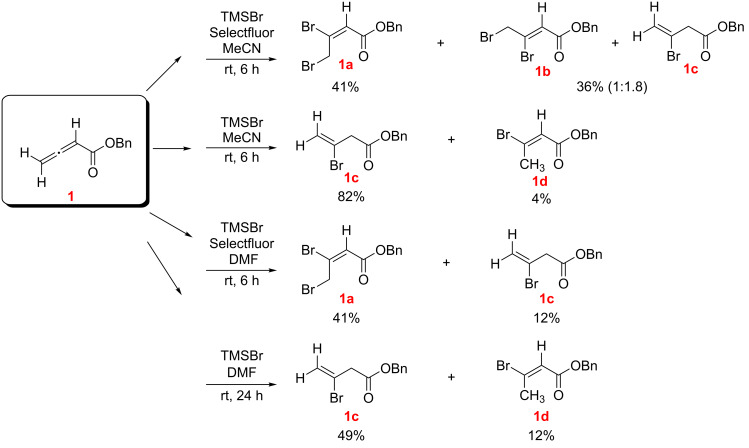
Reaction of benzyl allenoate (**1**) with TMSBr with and without Selectfluor (*E/Z* designations, as illustrated, were made by NMR; see Supporting Information File 1).

The reaction of benzyl allenoate (**1**) with TMSBr (2 equivalents) and Selectfluor (1 equivalent) (Scheme 1) in MeCN as solvent gave the regioisomeric 2,3-dibromoalkenoates **1a**, **1b** as major products along with the hydrobromination product **1c** (compounds **1b** and **1c** were inseparable by chromatography and were isolated together). In the absence of Selectfluor, dibromination products were not observed and the major product was **1c** along with tiny amounts of isomeric **1d**.

Switching to DMF as solvent and in the presence of Selectfluor, the 2,3-dibromoalkenoate **1a** and hydrobromination product **1c** were obtained, with **1c** isolated as a minor component, whereas in the absence of Selectfluor **1c** became the predominant product, and minor amounts of the isomeric **1d** was also isolated (Scheme 1).

**Scheme 2:**
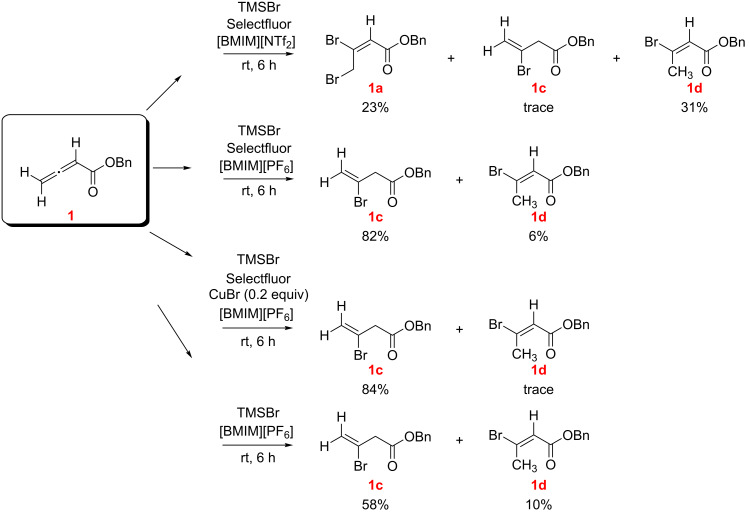
Reaction of benzyl allenoate (**1**) with TMSBr with and without Selectfluor in IL solvents (*E/Z* designations, as illustrated, were made by NMR; see Supporting Information File 1).

Switching to imidazolium ILs as solvent (Scheme 2), from the reaction of **1** with TMSBr/Selectfluor in [BMIM][NTf_2_] the dibromoalkenoate **1a** and the hydrobromination product **1d** were isolated in comparable amounts along with a trace of **1c**. Surprisingly no dihalogenation products were isolated when [BMIM][PF_6_] was employed as solvent. In this case **1c** was isolated as a major product along with minor amounts of **1d**. In an effort to enhance the oxidative power of Selectfluor and to promote dihalogenation, CuBr was used as an additive [16–17], but the outcome remained unchanged. In the absence of Selectfluor the same hydrohalogenation products **1c** and **1d** were isolated but in different ratios (Scheme 2).

**Scheme 3:**
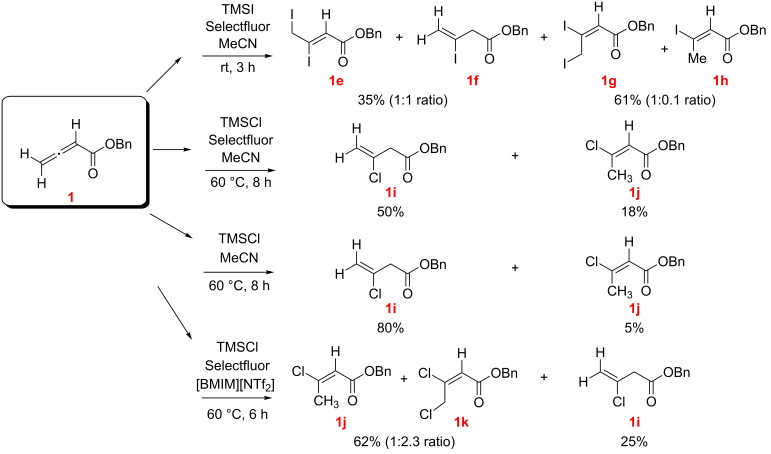
Reaction of benzyl allenoate (**1**) with TMSI and TMSCl, with and without Selectfluor (*E/Z* designations, as illustrated, were made by NMR; see Supporting Information File 1).

The reaction of benzyl allenoate (**1**) with TMSI in MeCN (Scheme 3) produced four products, namely the regioisomeric diiodoalkenoates **1e**, **1g** and the isomeric HI addition products **1f** and **1h** (compounds **1e/1f** and **1g**/**1h** were chromatographically inseparable and were isolated in pairs). Overall, the proportion of the oxidative dihalogenation products was notably larger than the hydroiodination products.

The reaction of allenoate **1** with TMSCl/Selectfluor in MeCN (Scheme 3) gave only the hydrochlorination products **1i** (major) and **1j** (minor). The same products were isolated in the absence of Selectfluor but with higher proportion of **1i**. Interestingly, repeating the reaction in [BMIM][NTf_2_] as the solvent (Scheme 3) resulted in the formation of the dichloroalkenoate **1k** as a major component, along with isomeric **1j** and **1i** (**1k** and **1j** were inseparable by chromatography and were isolated together).

**Scheme 4:**
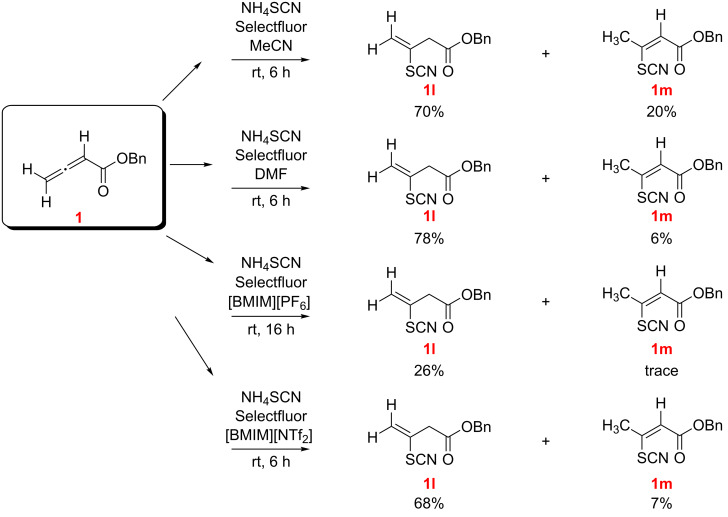
Reaction of benzyl allenoate (**1**) with NH_4_SCN/Selectfluor in different solvents (*E/Z* designations, as illustrated, were made by NMR; see Supporting Information File 1).

Focusing on thiocyanation, the reaction of benzyl allenoate (**1**) was studied with NH_4_SCN/Selectfluor in MeCN, DMF, as well as in [BMIM][PF_6_] and [BMIM][NTf_2_]. The isomeric conjugate addition products **1l** and **1m** were isolated, with **1l** as the major isomer. Unlike previous findings with 1-arylallenes [18], no dithiocyanation products were found irrespective of the choice of solvent (Scheme 4).

**Figure 1:**
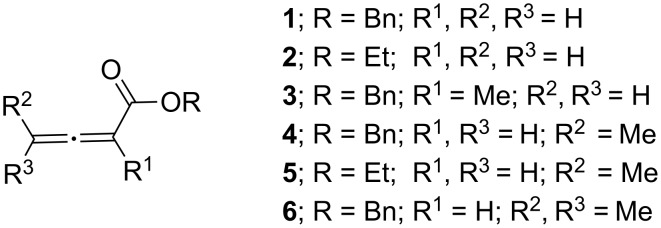
Allene esters synthesized for this study.

In order to examine a possible influence of the structure of the allenoate on the product distribution, allene esters **2**–**6** were synthesized (Figure 1).

**Scheme 5:**
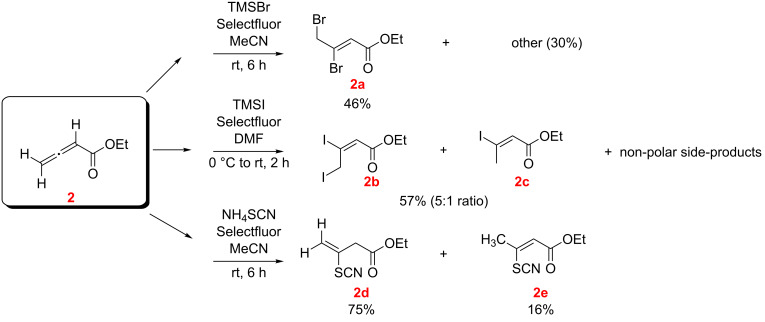
Reaction of ethyl allenoate (**2**) with TMSX/Selectfluor and NH_4_SCN/Selectfluor (*E/Z* designations, as illustrated, were made by NMR; see Supporting Information File 1).

The reactions of ethyl allenoate (**2**) with TMSBr, TMSI and with NH_4_SCN were studied in the presence of Selectfluor in MeCN and DMF (Scheme 5). With TMSBr and TMSI the dibromo- and the diiodoalkenoates **2a** and **2b** were isolated as the main products, respectively, along with minor amounts of **2c** (accompanied by unidentified side products). With NH_4_SCN however only the conjugate addition products **2d** and **2e** were observed.

**Scheme 6:**
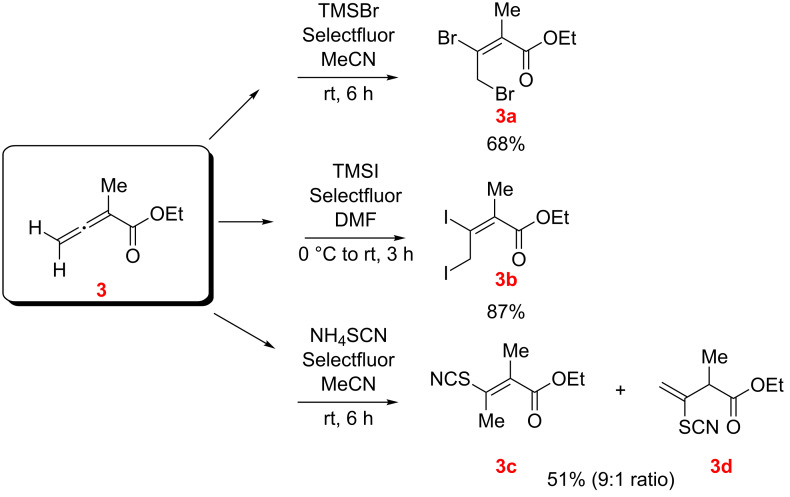
Reaction of ethyl allenoate **3** with TMSX/Selectfluor and NH_4_SCN/Selectfluor (*E/Z* designations, as illustrated, were made by NMR; see Supporting Information File 1).

The reactions listed in Scheme 5 were then repeated with ethyl allenoate **3** in MeCN as the solvent and the results are sketched in Scheme 6. The diiodo- and dibromoalkenoates **3a** and **3b** were obtained in good isolated yields. With NH_4_SCN/Selectfluor, on the other hand, only the isomeric conjugate addition (hydrothiocyantion) products **3c** and **3d** were isolated.

**Scheme 7:**
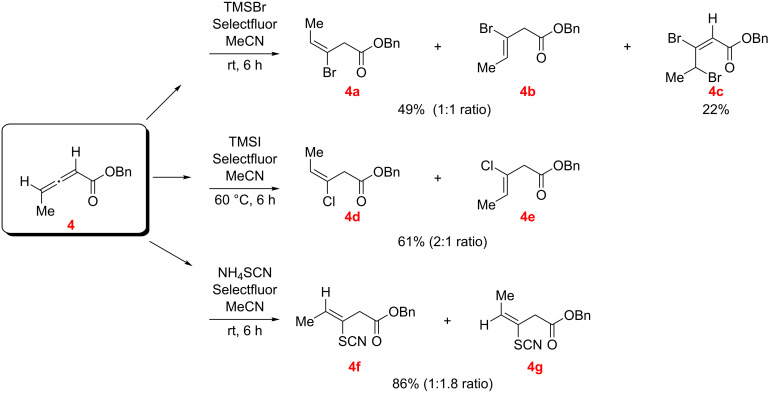
Reaction of benzyl allenoate **4** with TMSX (X = Br, and Cl)/Selectfluor and NH_4_SCN/Selectfluor (*E/Z* designations, as illustrated, were made by NMR; see Supporting Information File 1).

The reaction of benzyl allenoate **4** was examined with TMSX/Selectfluor (X = Br and Cl) and with NH_4_SCN/Selectfluor in MeCN as the solvent (Scheme 7). The dibromoalkenoate **4c** was isolated as a minor component in the reaction with TMSBr/Selectfluor, along with regioisomeric hydrobromination products **4a**, **4b** (in a 1:1 ratio by NMR; this fraction also contained traces of unreacted **4**). With TMSCl/Selectfluor and NH_4_SCN/Selectfluor only the corresponding regioisomeric conjugate addition products **4d**, **4e** and **4f**, **4g** were isolated.

**Scheme 8:**
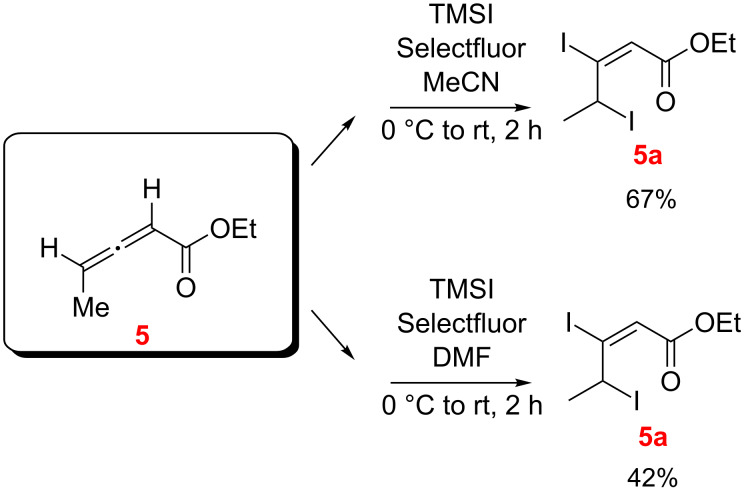
Reaction of ethyl allenoate **5** with TMSI/Selecfluor in DMF and MeCN as the solvents (*E/Z* designations, as illustrated, were made by NMR; see Supporting Information File 1).

The diiodoalkenoate **5a** was obtained in good isolated yield from the reaction of ethyl allenoate **5** with TMSI/Selectfluor in DMF and in MeCN and the conjugate addition products were not observed (Scheme 8).

**Scheme 9:**
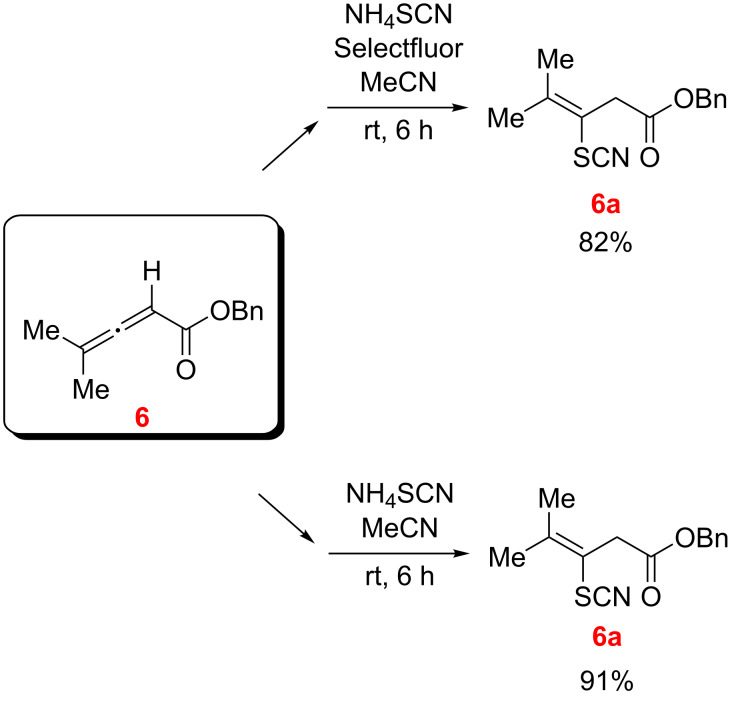
Reaction of benzyl allenoate **6** with NH_4_SCN in presence and absence of Selectfluor.

Finally, the reaction of benzyl allenoate **6** with NH_4_SCN with and without Selectfluor was studied in MeCN as the solvent. Consistent with earlier cases examined, the conjugate addition product **6a** was isolated in good yield, and products resulting from oxidative dithiocyanation were not observed (Scheme 9).

### Comparative discussion

Collectively, the comparative product analysis studies described herein demonstrate that the efficacy of Selectfluor in promoting oxidative/electrophilic dihalogenation (with TMSX) and dithiocyanation (with NH_4_SCN), which were previously studied in reactions with 1-arylallenes [18], is notably diminished toward electron-deficient allenoates, whereby the nucleophilic conjugate addition effectively competes, resulting in mixtures of both types of products.

**Scheme 10:**
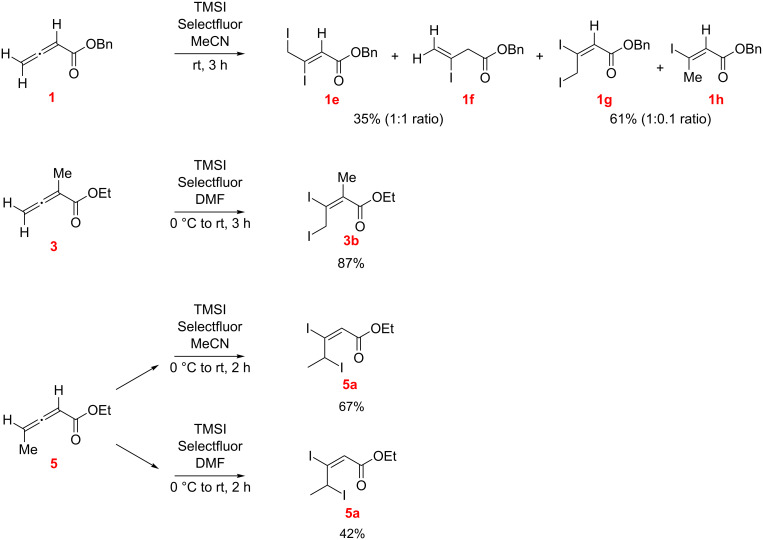
Influence of allenoate structure.

The electrophilic dihalogenation competes most effectively in the case of the TMSI/Selectfluor system leading to 2,3-diiodoalkenoates as the major products. By contrast, conjugate addition products are predominantly formed with TMSCl/Selectfluor and exclusively with NH_4_SCN/Selectfluor. These competing pathways are influenced by the nature of the anion, reflecting the ease of X^−^ → “X^+^” oxidative transformation, and the choice of the solvent [25]. The increased chemoselectivity (and yields) toward formation of the 2,3-dihalogenation products observed with the methyl-substituted allenoates **3** and **5**, especially in reactions with TMSI/Selectfluor (see Scheme 10), appears consistent with stabilization of the incipient allenyl cation in the oxidative/electrophilic pathway.

## Supporting Information

File 1 Experimental section.

File 2 NMR spectra.
