# Supplementary material for: Reaction of allene esters with Selectfluor/TMSX (X = I, Br, Cl) and Selectfluor/NH4SCN: Competing oxidative/electrophilic dihalogenation and nucleophilic/conjugate addition
Source: Beilstein J Org Chem. 2015 Sep 16;11:1641–8. doi: 10.3762/bjoc.11.180 (PMC4660907; doi:10.3762/bjoc.11.180)
Supplement: File 1 — Experimental section. [file Beilstein_J_Org_Chem-11-1641-s001.pdf]

# Supporting Information File 1

# for

# Reaction of allene esters with Selectfluor/TMSX (X = I, Br, Cl) and Selectfluor/$NH_4SCN$: Competing oxidative/electrophilic dihalogenation and nucleophilic/conjugate addition

A. Srinivas Reddy, Kenneth K. Laali*[§]

Address: Department of Chemistry, University of North Florida, 1 UNF Drive, Jacksonville, Florida 32224, USA

Email: Kenneth K. Laali - kenneth.laali@UNF.edu

*Corresponding author

[§]Tel: 904-620-1503, Fax: 904-620-3535

## Experimental Section

**General**. The allene esters were synthesized according to the literature methods [1-4]. Reagents were high purity commercial samples and were used without further purification. HPLC-grade MeCN and dry DMF were used. Imidazolium ionic liquids [BMIM][$NTf_2$] and [BMIM][$PF_6$] were prepared as previously described [5]. Column chromatography was performed on silica gel (63–200 mesh). NMR spectra were recorded in $CDCl_3$ using a 500 MHz instrument. MS analyses were performed at UNC-Wilmington (HRMS), at University of South Florida (ESIMS), and at Ionsense.Inc (HRMS). GC, GC–MS and FTIR analyses were performed in-house on standard research grade instruments.

**General procedure 1:** To a stirred solution of the allenoate (0.5 mmol) in MeCN or DMF, Selectfluor (0.5 mmol) and TMSX (X = I, Br, or Cl) (1.0 mmol) or $NH_4SCN$ (1.0 mmol) were added at room temperature under a nitrogen atmosphere, and the reaction mixture was stirred at the required temperatures (typically at rt for TMSBr and TMSI and at 60 °C for TMSCl). After completion of the reaction (monitored by TLC), the reaction mixture was diluted with diethyl ether (30 mL) and was washed with aq saturated $NaHCO_3$ followed by water, dried ($MgSO_4$), evaporated, and purified by silica-gel column chromatography using 2% to 5% diethyl ether in hexane as eluent. The same general procedure was used for reactions performed in the absence of Selectfluor.

**General procedure 2:** In reactions using imidazolium IL as the solvent, the experiments were performed in small Schlenk tubes under a nitrogen atmosphere (using 2 mL of the ionic liquid). The order of addition of reagents was changed as follows: Selectfluor (0.5 mmol) and TMSX (X = Br, Cl or I) or $NH_4SCN$ (1.0 mmol) were mixed with the ionic liquid (2 mL) and the allenoate (0.5 mmol) was then introduced with efficient stirring. After completion of the reaction as monitored by TLC (refer to schemes) the reaction mixture was diluted with diethyl ether (30 mL) and was washed with aq saturated $NaHCO_3$ followed by water, dried ($MgSO_4$), evaporated, and purified by silica-gel column chromatography using 2% to 5% diethyl ether in hexane as eluent.

**(*E*)-Benzyl 3,4-dibromobut-2-enoate (1a)**: colorless liquid. 41% yield (GC purity assay > 98%). $^1$H NMR ($CDCl_3$, 500 MHz): $\delta$ 7.40-7.37 (m, 5H), 6.46 (s, 1H), 5.19 (s, 2H), 4.87 (s, 2H). NOE: irradiation of δ 6.46 signal resulted in no enhancement in δ 4.87 signal. $^{13}$C NMR ($CDCl_3$, 125 MHz): $\delta$ 163.4, 142.1, 135.2, 128.8, 128.7, 128.5, 125.6, 67.1, 31.8. IR ($cm^{-1}$): 3032, 2931, 1715, 1620, 1327, 1195, 965, 746. MS (HRMS-ESI): calcd for $C_{11}H_{11}Br_2O_2$ $[M+H]^+$ 335.0113, found 335.0377.

**(*Z*)-Benzyl 3,4-dibromobut-2-enoate (1b) and benzyl 3-bromo-but-3-enoate (1c)**: colorless liquid. 36% combined yield (ratio 1 to 1.8 by NMR and 1 to 2.4 by GC). $^1$H NMR ($CDCl_3$, 500 MHz): $\delta$ 7.40-7.35 (m), 6.75 (br s),[a] 5.79-5.78 (m),[b] 5.64 (d, *J* = 1.9 Hz ),[b] 5.22 (s),[a] 5.20 (s),[b] 4.29 (s),[a] 3.55 (d, *J* = 1.0 Hz)[b]. NOE**:** irradiation of $\delta$ 6.75 and $\delta$ 4.29 signals resulted in mutual enhancement. $^{13}$C NMR ($CDCl_3$, 125 MHz): $\delta$ 168.8, 135.5, 135.4, 128.7,

128.7, 128.6, 128.5, 128.4, 128.4, 123.8, 122.7, 121.5, 67.2,[b] 66.9, [a] 46.8,[b] 37.0[a]. IR ($cm^{-1}$): 2920, 1730, 1631, 1454, 1161, 1001, 745. MS (GC-MS): m/z 253 [$M-H^+$][b] *(a and b designations refer to specific signals for **1b** and **1c** respectively).*

**Benzyl 3-bromobut-3-enoate (1c):** colorless liquid; variable yields (refer to Schemes 1-2). GC purity assay > 99%. $^1$H NMR ($CDCl_3$, 500 MHz): $\delta$ 7.39-7.35 (m, 5H), 5.79 (s, br; 1H), 5.65 (d, *J* = 1.5 Hz, 1H), 5.20 (s, 2H), 3.55 (s, 2H). $^{13}$C NMR ($CDCl_3$, 125 MHz): $\delta$ 168.8, 135.5, 128.6, 128.5, 128.4, 123.8, 121.5, 67.1, 46.8. IR ($cm^{-1}$): 2917, 1735, 1632, 1166, 898, 742. MS (GC-MS): *m/z* 254 [$M^+$]. MS (HRMS-ESI): calcd for $C_{11}H_{11}BrNaO_2$ $[M+Na]^+$ 277.1119, found 276.9834.

**(*E*)-Benzyl 3-bromobut-2-enoate (1d):** colorless liquid; minor product (refer to Schemes 1-2). GC purity assay > 99%. $^1$H NMR ($CDCl_3$, 500 MHz): $\delta$ 7.38-7.35 (m, 5H), 6.40 (s, 1H), 5.16 (s, 2H), 2.80 (s, 2H). NOE: irradiation of $\delta$ 6.40 and $\delta$ 2.80 resonances resulted in no mutual enhancement. $^{13}$C NMR ($CDCl_3$, 125 MHz): $\delta$ 164.3, 144.7, 135.7, 128.7, 128.5, 128.4, 123.1, 66.4, 26.5. IR ($cm^{-1}$): 2922, 1718, 1330, 1173, 860. MS (HRMS-ESI): calcd for $C_{11}H_{11}BrNaO_2$ $[M+Na]^+$ 277.1119, found 276.9834.

**(*Z*)-Benzyl 3,4-diiodobut-2-enoate (1e)[a] and benzyl 3-iodobut-3-enoate (1f)[b]**: reddish liquid. 35% combined yield (ratio 1 to 1 by NMR and 1 to 1:15 by GC). $^1$H NMR ($CDCl_3$, 500 MHz): $\delta$ 7.39-7.34 (m), 6.70 (s; 1H),[a] 6.20 (d, *J* = 0.9 Hz; 1H),[b] 5.92 (d, *J* = 1.9 Hz; 1H),[b] 5.20 (s; 2H),[a] 5.18 (s; 2H),[b] 4.57 (s; 2H),[a] 3.62 (s, 2H)[b]. $^{13}$C NMR ($CDCl_3$, 125 MHz): $\delta$ 169.1,[b] 163.8,[a] 135.5, 135.4, 130.6; 128.7, 128.7, 128.6, 128.5, 128.5, 127.2, 114.8,[a] 97.5,[b] 67.18, 66.9, 50.6,[b] 17.6[a]. NOE: irradiation of $\delta$ 6.7 signal resulted in enhancement in $\delta$ 4.57. IR ($cm^{-1}$, mixture): 2920, 1723, 1608, 1453, 911. MS (GC-MS): *m/z* 302 [$M^+$] [b]. HRMS (ESI-DART) (positive ion mode): 428.8835 [$M^+$][a] and 445.9102 [$M^+/NH_3$][a] *(a and b designations refer to specific signals for **1e** and **1f** respectively).*

**(*E*)-Benzyl 3,4-diiodobut-2-enoate (1g)[a] and (*E*)-benzyl 3-iodobut-2-enoate(1h)[b]:** reddish liquid**.** 61% combined yield (ratio 1 to 0.1 by NMR and 80/20 by GC). $^1$H NMR ($CDCl_3$, 500 MHz): $\delta$ 7.40-7.34 (m), 6.70-6.69 (m),[b] 6.63 (s; 1H),[a] 5.18 (s; 2H),[a] 5.14 (s),[b] 5.08 (s; 2H),[a] 3.01 (d, *J* = 1.5 Hz)[b]. $^{13}$C NMR ($CDCl_3$, 125 MHz): $\delta$ 163.6, 135.3, 131.4, 131.2, 128.7, 128.6, 128.4, 128.4, 128.5, 122.4, 66.8,[a] 66.3,[b] 29.8,[b] 10.8[a]. NOE**:** irradiation of $\delta$ 6.63 and $\delta$ 5.08 signals resulted in no mutual enhancement; similarly irradiation of $\delta$ 6.69 and $\delta$ 3.30

resulted in no mutual enhancement. IR ($cm^{-1}$; mixture): 2918, 1711, 1597, 1327, 1185, 742. MS (GC-MS): *m/z* 302 [$M^+$],[b] ($M^+$ for **1g**: n.o.).

**Benzyl 3-chlorobut-3-enoate (1i)**: pale yellow liquid; variable yields (refer to Scheme 3). GC purity assay >99%. $^1$H NMR ($CDCl_3$, 500 MHz): $\delta$ 7.39-7.34 (m, 5H), 5.40 (d, *J* = 0.98, 1H), 5.36 (s, 1H), 5.20 (s, 2H), 3.43 (s, 2H). $^{13}$C NMR ($CDCl_3$, 125 MHz): 168.7, 135.5, 134.2, 128.7, 128.5, 128.3, 116.9, 67.1, 44.7. IR ($cm^{-1}$): 2923, 1735, 1683, 1133, 860, 736. MS (GC-MS): *m/z* 210 $[M]^+$,135 $[M-Cl]^+$, 129, 108, 92.

**(*E*)-Benzyl 3-chlorobut-2-enoate (1j):** pale yellow liquid in variable yields (refer to Scheme 3). GC purity assay ~90%. $^1$H NMR ($CDCl_3$, 500 MHz): $\delta$ 7.39-7.35 (m, 5H), 6.13 (unresolved q, *J* = < 1Hz, 1H), 5.16 (s, 2H), 2.59 (appearance s, 3H). NOE: irradiation of $\delta$ 6.13 and $\delta$ 2.59 resonances resulted in no mutual enhancement. $^{13}$C NMR ($CDCl_3$, 125 MHz): $\delta$ 164.5, 133.4, 128.7, 128.5, 128.4, 128.3, 118.9, 66.3, 23.8. IR ($cm^{-1}$): 2923, 1720, 1635, 1183, 1088, 912. MS (GC-MS): *m/z* 210 $[M]^+$, 175 $[M-Cl]^+$,147, 129, 103.

**(*E*)-Benzyl 3-chlorobut-2-enoate (1j) and (*E*)-benzyl 3,4-dichlorobut-2-enoate (1k):** colorless liquid. 62% combined isolated yield (ratio 1 to 2.3 by NMR and 32/68 by GC). $^1$H NMR ($CDCl_3$, 500 MHz): $\delta$ 7.39-7.36 (m), 6.26 (s),[b] 6.14 (d, *J* = 1.4 Hz),[a] 5.20 (s),[b] 5.17 (s),[a] 4.83 (s),[b] 2.60 (d, *J* ~ 1:0 Hz)[a]. NOE: Irradiation of $\delta$ 6.14 and $\delta$ 2.60 signals resulted in no mutual enhancement, similarly irradiation of $\delta$ 6.26 and $\delta$ 4.83 signals resulted in no mutual enhancement.$^{13}$C NMR ($CDCl_3$, 125 MHz): $\delta$ 164.5,[a] 163.3,[b] 153.3,[a] 150.0,[b] 135.8, 135.2,[b] 128.8, 128.7, 128.7, 128.5, 128.4, 128.3, 122.1, 118.9, 67.0,[b] 66.3, 42.8,[b] 23.8[b]. IR ($cm^{-1}$, mixture): 3032, 1718, 1632, 1453, 1332, 1160, 741. MS (GC-MS): *m/z* 210 [$M^+$][a] and 245 [$M^+$][b] (*a and b designations refer to specific signals for **1j** and **1k** respectively).*

**Benzyl 3-thiocyanatobut-3-enoate (1l ):** pale-yellow liquid in variable isolated yields (refer to Scheme 4). GC purity assay > 99%. $^1$H NMR ($CDCl_3$, 500 MHz): $\delta$ 7.39-7.36 (m, 5H), 5.75 (d, *J* = 1.5 Hz, 1H), 5.67 (brs, 1H), 5.19 (s, 2H), 3.50 (s, 2H). $^{13}$C NMR ($CDCl_3$, 125 MHz): 168.4, 135.1, 128.7, 128.7, 128.5, 127.8, 124.0, 109.6, 67.5, 41.7. IR ($cm^{-1}$): 2847, 2156, 1733, 1623, 1172, 905, 726. MS (HRMS-ESI): calcd for $C_{12}H_{11}NNaO_2S$ $[M+Na]^+$ 256.0403, found 256.0417.

**(*Z*)-Benzyl 3-thiocyanatobut-2-enoate (1m):** pale-yellow liquid; minor product in variable isolated yields (refer to Scheme 4). GC purity assay > 99%. $^{1}$H NMR ($CDCl_3$, 500 MHz): $\delta$ 7.38-7.37 (m, 5H), 6.25 (s, 1H), 5.19 (s, 2H), 2.54 (s, 3H). NOE**:** irradiation of $\delta$ 6.25 resulted in enhancement of $\delta$ 2.54. $^{13}$C NMR ($CDCl_3$, 125 MHz): $\delta$ 165.8, 147.6, 135.2, 128.8, 128.7, 128.5, 117.1, 110.2, 67.0, 26.0. IR (cm$^{-1}$): 2917, 2848, 2157, 1691, 1606, 1319, 1187, 1035, 832. MS (HRMS-ESI): calcd for $C_{12}H_{11}NNaO_2S$ $[M+Na]^+$ 256.0403, found 256.0411.

**(*Z*)-Ethyl 3,4-dibromobut-2-enoate (2a)**: colorless liquid. 46% yield (GC purity assay > 99%). $^{1}$H NMR ($CDCl_3$, 500 MHz): $\delta$ 6.68 (s, 1H), 4.28 (d, *J* = 0.9 Hz, 2H), 4.43 (q, *J* = 7.3 Hz, 2H), 1.31 (t, *J* = 7.3 Hz). NOE**:** irradiation of $\delta$ 6.68 signal resulted in enhancement at $\delta$ 4.28 signal. $^{13}$C NMR ($CDCl_3$, 125 MHz): $\delta$ 163.7, 133.6, 123.0, 61.2, 37.1, 14.2. IR (cm$^{-1}$): 2979, 1724, 1630, 1024, 864. MS (ESI): *m/z* 272.8 $[M+H]^+$.

**(*E*)-Ethyl 3,4-diiodobut-2-enoate (2b) and (*E*)-ethyl 3-iodobut-2-enoate (2c):** reddish liquid. 57% yield (ratio 5:1 by NMR). $^{1}$H NMR ($CDCl_3$, 500 MHz): $\delta$ 6.63 (d, *J* = 1.5 Hz, 1H),$^{b}$ 6.57 (s, 1H),$^{a}$ 5.07 (s, 2H),$^{a}$ 4.20 (q, *J* = 7.3 Hz, 2H),$^{a}$ 4.17 (q, *J* = 7.3 Hz),$^{b}$ 2.99 (d, *J* ~ 1:0 Hz),$^{b}$ 1.30 (t, *J* = 7.3 Hz, 3H),$^{a}$ 1.28 (t, *J* = 7.3 Hz)$^{b}$. NOE: Irradiation of $\delta$ 6.57 and $\delta$ 5.07 signals resulted in no mutual enhancement, similarly irradiation of $\delta$ 6.63 and $\delta$ 2.99 resonances led to no mutual enhancement. $^{13}$C NMR ($CDCl_3$, 125 MHz): $\delta$ 164.2,$^{b}$ 163.8,$^{a}$ 131.8$^{a}$, 131.6,$^{b}$ 121.6,$^{a}$ 120.4,$^{b}$ 61.1,$^{a}$ 60.5,$^{b}$ 31.1,$^{b}$ 14.3$^{b}$, 14.2,$^{b}$ 10.9$^{a}$. IR (cm$^{-1}$, mixture): 2976, 1709, 1599, 1327, 742. MS (GC-MS): *m/z* 365 $[M-H]^{+a}$ and 239 $[M-H]^{+b}$ (*a and b designations refer to specific signals for **2b** and **2c** respectively).*

**Ethyl 3-thiocyanatobut-3-enoate (2d):** colorless liquid. 75% yield (GC purity assay > 99%). $^{1}$H NMR ($CDCl_3$, 500 MHz): $\delta$ 5.74 (d, *J* = 1.9 Hz, 1H), 5.67-5.66 (m, 1H), 4.21 (q, *J* = 6.8 Hz, 2H), 3.44 (d, *J* = 0.9 Hz, 2H), 1.29 (t, *J* = 6.8 Hz, 3H). $^{13}$C NMR ($CDCl_3$, 125 MHz): 168.5, 128.0, 123.8, 109.7, 61.8, 41.8, 14.1. IR (cm$^{-1}$): 2916, 2156, 1731, 1623, 1328, 1185, 1026. MS (GC-MS): *m/z* 171 $[M]^+$. HRMS (ESI-DART): 172.0423$[M+H]^+$and 189.0689 $[M^+/NH_3]$.

**(*Z*)-Ethyl 3-thiocyanatobut-2-enoate (2e):** colorless liquid (minor product, refer to Scheme 5). $^{1}$H NMR ($CDCl_3$, 500 MHz): $\delta$ 6.19 (s, 1H), 4.21 (q, *J* = 7.34 Hz, 2H), 2.52 (s, 3H), 1.30 (t, *J* = 7.34 Hz, 3H). IR (cm$^{-1}$): 2952, 2151, 1718, 1627, 1458, 1190, 766, 628.

**(*E*)-Ethyl 3,4-dibromo-2-methylbut-2-enoate (3a)**: colorless liquid. 68% yield (GC purity assay > 99%). $^1$H NMR ($CDCl_3$, 500 MHz): $\delta$ 4.73 (s, 2H), 4.27 (q, *J* = 6.8 Hz, 2H), 2.11 (s, 3H), 1.34 (t, *J* = 6.8 Hz, 3H). NOE: irradiation of $\delta$ 4.73 and $\delta$ 2.11 signals resulted in no mutual enhancement. $^{13}$C NMR ($CDCl_3$, 125 MHz): $\delta$ 165.5, 134.6, 132.9, 61.9, 35.4, 21.5, 14.2. IR (cm$^{-1}$): 2979, 1712, 1629, 1256, 1130, 765. MS (HRMS-ESI): calcd for $C_7H_{10}Br_2NaO_2$ $[M+Na]^+$ 306.9795, found 306.8948.

**(*E*)-Ethyl 3,4-diiodo-2-methylbut-2-enoate (3b):** reddish liquid. 87% yield. $^1$H NMR ($CDCl_3$, 500 MHz): $\delta$ 4.9 (s, 2H), 4.26 (q, *J* = 6.85 Hz, 2H), 2.09 (s, 3H), 1.35 (t, *J* = 6.85 Hz, 3H). NOE: irradiation of $\delta$ 4.9 and $\delta$ 2.09 signals resulted in no mutual enhancement. $^{13}$C NMR ($CDCl_3$, 125 MHz): $\delta$ 164.2, 135.9, 117.6, 61.7, 27.8, 15.7, 14.3. IR (cm$^{-1}$): 2976, 1706, 1587, 1235, 1116, 976. MS (HRMS-ESI): calcd for $C_7H_{10}I_2NaO_2$ $[M+Na]^+$ 402.9518 found: 402.8668 $[M+Na]^+$.

**(*E*)-Ethyl 2-methyl-3-thiocyanatobut-2-enoate (3c) and ethyl 2-methyl-3-thiocyanato-but-3-enoate (3d):** pale-yellow liquid. $^1$H NMR ($CDCl_3$, 500 MHz): $\delta$ 5.76 (m, unresolved),[b] 5.69 (m, unresolved),[b] 4.24 (q, *J* = 6.8 Hz, 2H),[a] 4.20 (q, *J* = 6.8 Hz),[b] 2.46 (pseudo-s, 3H),[a] 2.05 (s, 3H),[a] 1.47 (Me, unresolved),[b] 1.34-1.31(m, 3H),[a] 1.31-1.28 (m)[b]. NOE**:** irradiation of $\delta$ 2.46 and $\delta$ 2.05 signals resulted in no mutual enhancement. $^{13}$C NMR: $\delta$ 169.2,[b] 167.5,[a] 138.5,[a] 133.4,[b] 124.3,[a] 121.3,[b] 112.4,[a] 61.8,[a] 61.0,[b] 46.4,[b] 22.8,[a] 17.8,[a] 15.9,[b] 14.2,[a] 14.1[b]. IR (cm$^{-1}$; mixture): 2981, 2152, 1714, 1684, 1592, 1319, 1192, 854,768. MS (GC-MS): *m/z* 185 $[M]^+$, 158, 140, 127 $[M-SCN]^+$, 112, 99 (*a and b designations refer to specific signals for* ***3c*** *and* ***3d*** *respectively).*

**(*E*)-Benzyl 3-bromopent-3-enoate (4a) and (*Z*)-benzyl 3-bromopent-3-enoate (4b):** colorless liquid, 49% combined yield including traces of unreacted **4** (1: 1 *Z/E* isomer ratio by NMR, 46 : 38 ratio by GC assay). $^1$H NMR ($CDCl_3$, 500 MHz)**:** 7.40-7.33 (m), 6.21 (q, *J* = 7.3 Hz), 6.16 (q, *J* = 6.8 Hz), 5.65-5.60 (m), 5.2 (s), 5.19 (s), 5.18 (s), 3.58 (s), 3.54 (s), 1.88 (d, *J* = 6.8 Hz), 1.81-1.77 (m), 1.68 (d, *J* =7.3 Hz). IR (cm$^{-1}$): 2918, 1732, 1598, 1162, 966, 736. MS (GC-MS): *m/z* 269 $[M+1]^+$. MS (HRMS-ESI) calcd for $C_{12}H_{13}BrNaO_2$ $[M+Na]^+$ 291.1385, found 290.9962.

**(*E*)-benzyl 2,3-dibromopent-3-enoate (4c):** pale-yellow liquid. 22% yield (GC purity assay > 98%). $^1$H NMR ($CDCl_3$, 500 MHz): $\delta$ 7.39-7.35 (m, 5H), 6.42 (q, *J* = 6.8 Hz, 1H), 6.36 (s, 1H), 5.18 (q, *J* = 12.2 Hz, 2H), 1.80 (d, *J* = 6.3 Hz, 3H). NOE: irradiation of $\delta$ 6.42 and

δ 6.36 signals resulted in no mutual enhancement. $^{13}$C NMR ($CDCl_3$, 125 MHz): δ 163.5, 150.2, 135.2, 128.8, 128.7, 128.5, 123.2, 67.0, 43.5, 25.3. IR ($cm^{-1}$): 2921, 1715, 614, 1184, 912, 744. MS (HRMS-ESI): calcd for $C_{12}H_{12}Br_2NaO_2$ $[M+Na]^+$ 369.0488, found 368.9106.

**(*Z*)-Benzyl 3-chloropent-3-enoate (4e) and (*E*)-benzyl 3-chloropent-3-enoate (4d):** pale yellow liquid. 61% combined yield (1: 2 ratio by NMR, 45/54 by GC assay-unresolved peak separation). $^1$H NMR ($CDCl_3$, 500 MHz): δ 7.40-7.33 (m), 5.90 (q, *J* = 7.5 Hz),[b] 5.74-5.73 (m),[a] 5.19 (s),[b] 5.18 (s),[a] 3.45 (s),[b] 3.39 (s),[a] 1.79-1.78 (m),[a] 1.70[b] (d, *J* = 6.8 Hz). NOE**:** irradiation of δ 5.74 resulted in enhancement at δ 3.39 and δ 1.77, and irradiation of δ 3.39 signal resulted in enhancement at δ 5.74, but irradiation of δ 5.90 and δ 3.45 signals resulted in no mutual enhancement. $^{13}$C NMR ($CDCl_3$, 125 MHz): δ 169.5,[a] 169.0,[b] 135.7, 128.6, 128.5, 128.5,[b] 128.4, 128.3, 128.2, 127.0,[a] 126.7,[b] 125.4,[a] 125.3,[b] 67.0, 66.9, 44.9,[a] 39.5,[b] 14.4,[a] 14.3[b]. IR ($cm^{-1}$; *Z/E* mixture): 3031, 2918, 1735, 1659, 1496, 1453, 1260, 969.
MS (HRMS-ESI): calcd for $C_{12}H_{13}ClNaO_2$ $[M+Na]^+$ 247.1891, found 247.0506 (*a* and *b designations refer to specific signals for* ***4e*** *and* ***4d*** *respectively).*

**(*Z*)-Benzyl 3-thiocyanatopent-3-enoate (4f) and (*E*)-benzyl 3-thiocyanatopent-3-enoate (4g)** colorless liquid. 86% combined yield (1: 1.8 ratio by NMR; 28/72 by GC assay-unresolved peak separation). $^1$H NMR ($CDCl_3$, 500 MHz): δ 7.41-7.34 (m), 6.37 (q, *J* = 6.8 Hz),[b] 6.14 (q, *J* = 6.8 Hz),[a] 5.19 (s),[a] 5.18 (s),[b] 3.53 (br s),[b] 3.52 (unresolved t , *J* ~ 1 Hz),[a] 1.95 (dd, *J* = 6.8 and 1.0 Hz)[a], 1.81 (d, *J* = 6.8 Hz)[b]. NOE**:** irradiation of δ 6.14 and δ 3.52 signals resulted in mutual enhancement but irradiation of δ 6.37 and δ 3.52 signals resulted in no mutual enhancement. $^{13}$C NMR ($CDCl_3$, 125 MHz): δ 169.4,[a] 168.6,[b] 139.5,[b] 138.4,[a] 135.3, 128.7, 128.6, 128.5, 128.5, 128.4, 128.4, 119.0, 118.1, 110.0,[b] 109.9,[a] 67.4,[b] 67.3,[a] 43.1,[a] 37.0,[b] 16.2,[a] 15.6[b]. MS (HRMS-ESI): calcd for $C_{13}H_{13}NNaO_2S$ $[M+Na]^+$ 270.3025, found: 270.0569. HRMS (ESI-DART) (positive ion) 248.0735 $[M+H]^+$ and 265.0998 $[M^+/NH_3]$. IR ($cm^{-1}$, isomer mixture): 3032, 2153, 1731, 1453, 1315, 1164, 733 (*a* and *b designations refer to specific signals for* ***4f*** *and* ***4g*** *respectively).*

**(*Z*)-Ethyl 3,4-diiodopent-2-enoate ( 5a):** major product in variable isolated yields (refer to Scheme 8). $^1$H NMR ($CDCl_3$, 500 MHz): δ 6.48 (s, 1H), 6.38 (q, *J* = 6.3 Hz, 1H), 4.19 (q, *J* = 7.3 Hz, 2H), 1.87 (d, *J* = 6.8 Hz, 3H), 1.30 (t, *J* = 7.3 Hz, 3H). NOE: Irradiation of δ 6.48 and 6.38 signals resulted in no mutual enhancement. $^{13}$C NMR ($CDCl_3$, 125 MHz):

$\delta$ 164.0, 136.2, 129.2, 61.1, 31.1, 24.5, 14.2. IR ($cm^{-1}$): 2978, 1709, 1592, 1336, 1288, 1147, 1102. MS (HRMS-ESI): calcd for $C_7H_{10}I_2NaO_2$ $[M+Na]^+$ 402.9518, found 402.8662.

**Benzyl 4-methyl-3-thiocyanatopent-3-enoate (6a):** colorless liquid. $^1H$ NMR ($CDCl_3$, 500 MHz): $\delta$ 7.39-7.34 (m, 5H), 5.18 (s, 2H), 3.59 (s, 2H), 2.09 (s, 3H), 1.88 (s, 3H). $^{13}C$ NMR ($CDCl_3$, 125 MHz); $\delta$ 169.5, 147.9, 135.4, 128.6, 128.5, 128.3, 111.0, 110.9, 67.2, 39.6, 23.8, 22.1. IR ($cm^{-1}$): 2916, 2151, 1730, 1453, 1162, 911, 695. MS (HRMS-ESI): calcd for $C_{14}H_{15}NNaO_2S$ $[M+Na]^+$ 284.0721, found 284.0723.

# References


1. Rout, L; Harned, A. M. *Chem. Eur. J.* **2009**, 15, 12926 – 12928
2. Trost, B. M.; Pinkerton, A.B.; Seidel, M. *J. Am. Chem. Soc.* **2001**, *123*, 12466-12476
3. Xu, C.-P.; Huang, P.-Q.; Py, S. *Org. Lett.*, **2012**, *14*, 2034-2037.
4. Keck, G. E.; Giles, R. L,; Cee, V. J.; Wager, C. A.; Yu, T.; Kraft, M. B. *J. Org. Chem.* **2008**, *73*, 9675–9691
5. Shukla, M.; Srivastava, N.; Saha, S. in: S.T. Handy (Ed.), Ionic Liquids-Classes and Properties, *In Tech*, **2011**, pp. 153–170 (Chapter 7).
