# Supplementary material for: Reaction of allene esters with Selectfluor/TMSX (X = I, Br, Cl) and Selectfluor/NH4SCN: Competing oxidative/electrophilic dihalogenation and nucleophilic/conjugate addition
Source: Beilstein J Org Chem. 2015 Sep 16;11:1641–8. doi: 10.3762/bjoc.11.180 (PMC4660907; doi:10.3762/bjoc.11.180)
Supplement: File 2 — NMR spectra. [file Beilstein_J_Org_Chem-11-1641-s002.pdf]

Supporting Information File 2

for

Reaction of allene esters with Selectfluor/TMSX (X = I, Br, Cl) and Selectfluor/NH_4SCN: Competing oxidative/electrophilic dihalogenation and nucleophilic/conjugate addition

A. Srinivas Reddy, Kenneth K. Laali*[§]

Address: Department of Chemistry, University of North Florida, 1 UNF Drive, Jacksonville, Florida 32224, USA

Email: Kenneth K. Laali - kenneth.laali@UNF.edu

*Corresponding author

[§]Tel: 904-620-1503, Fax: 904-620-3535

NMR spectra

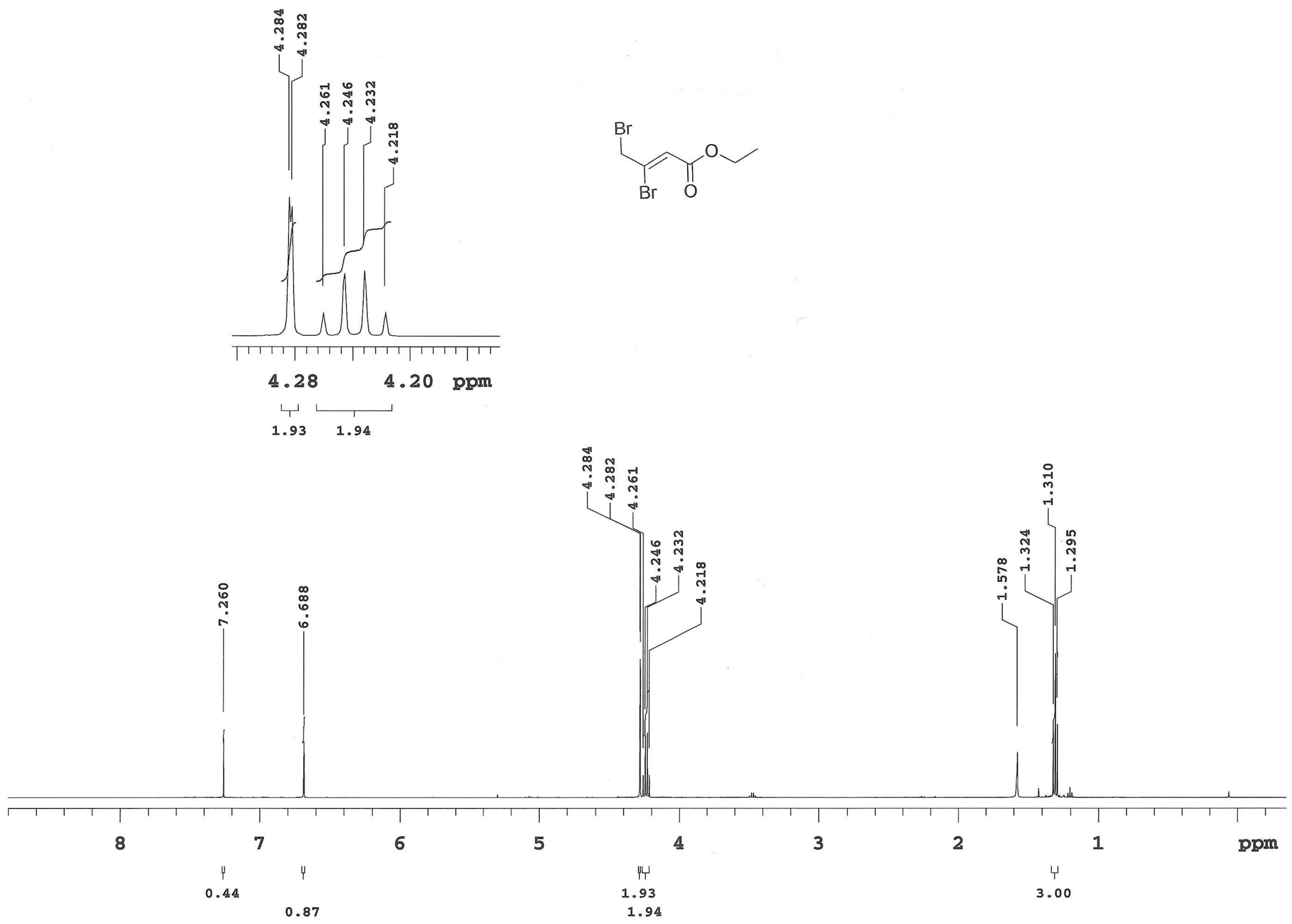
Br
Br
O
O
4.284
4.282
4.261
4.246
4.232
4.218
4.28
4.20
ppm
1.93
1.94
7.260
6.688
1.578
1.324
1.310
1.295
8
7
6
5
4
3
2
1
ppm
0.44
0.87
1.93
1.94
3.00

A-1

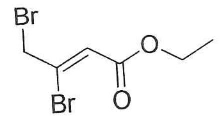
Br
Br
O
O

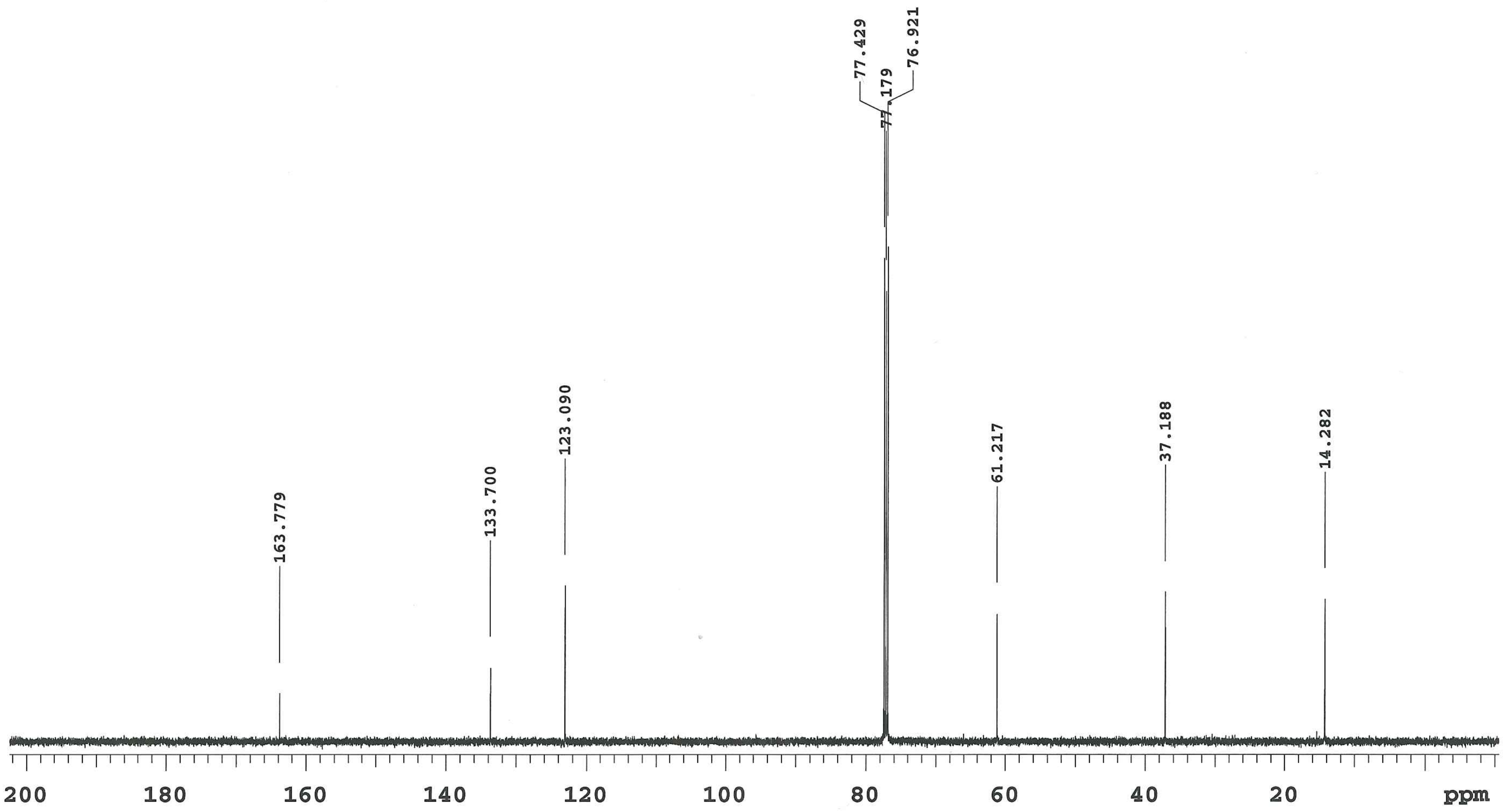
163.779
133.700
123.090
77.429
77.179
76.921
61.217
37.188
14.282
200
180
160
140
120
100
80
60
40
20
ppm

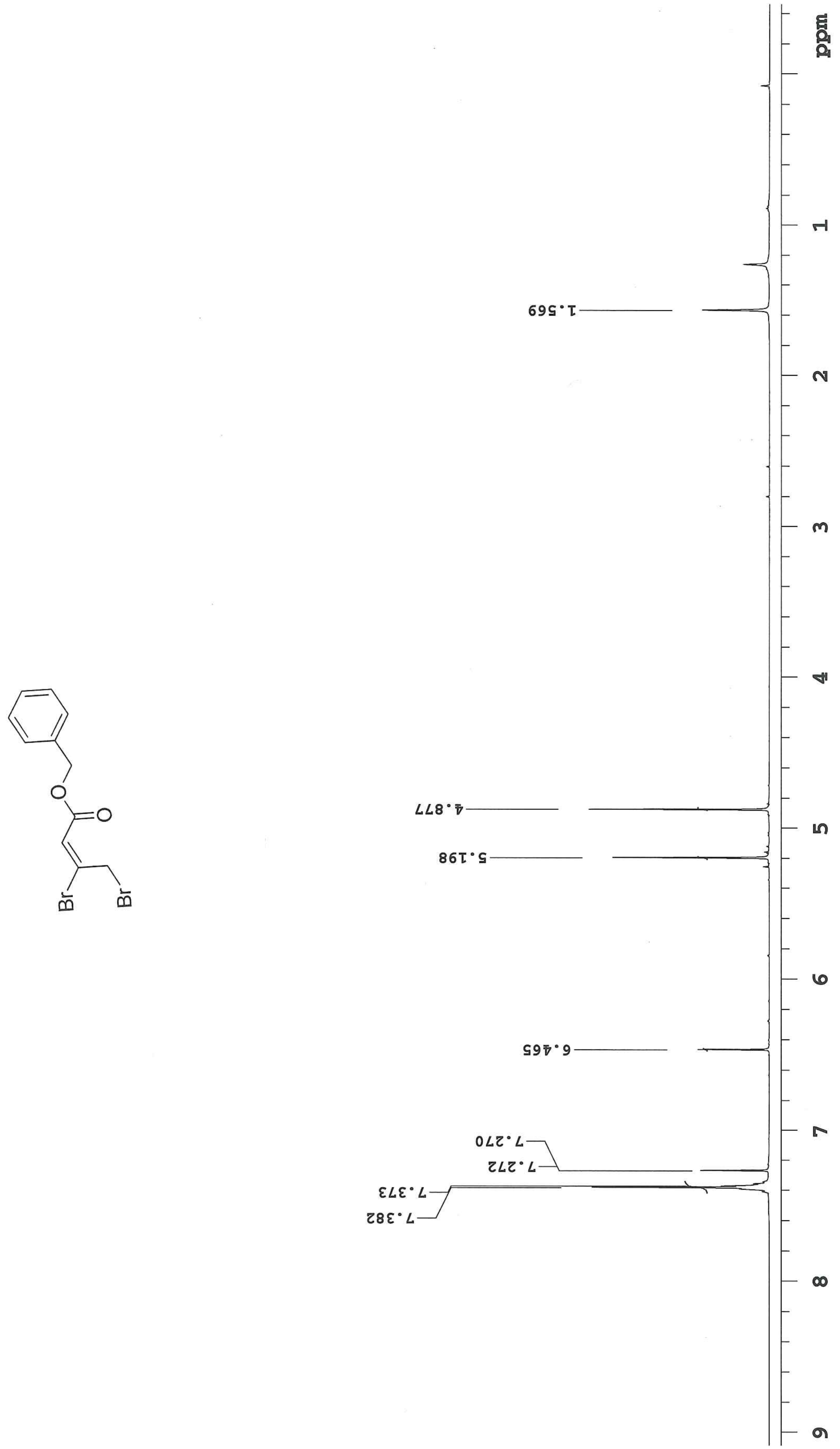
Br
Br
O
O
1.569
4.877
5.198
6.465
7.270
7.272
7.373
7.382
2.07
2.00
0.78
4.88
ppm
1
2
3
4
5
6
7
8
9

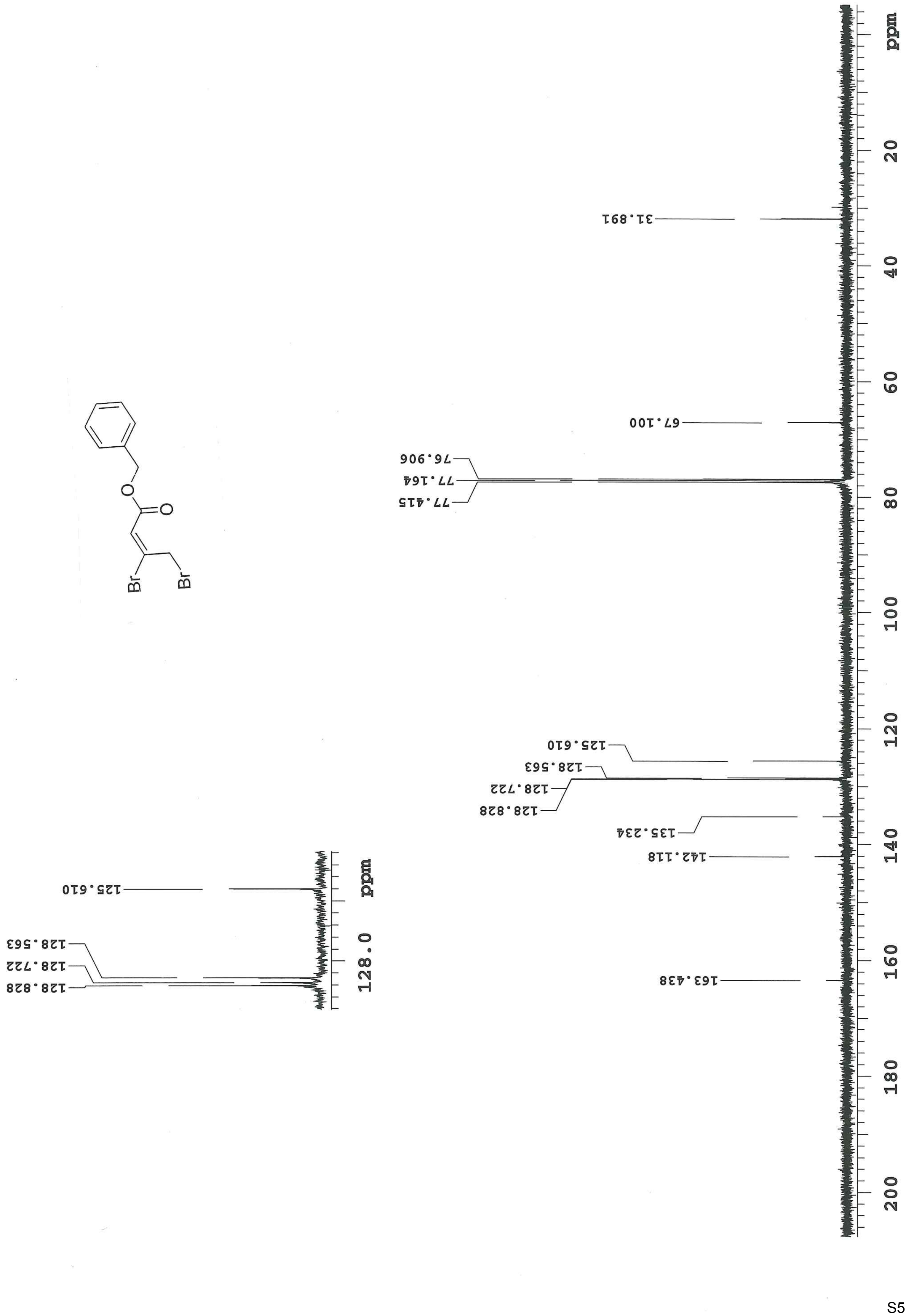
Br
Br
O
O
31.891
67.100
76.906
77.164
77.415
125.610
128.563
128.722
128.828
135.234
142.118
163.438
ppm
20
40
60
80
100
120
140
160
180
200
125.610
128.563
128.722
128.828
128.0
ppm

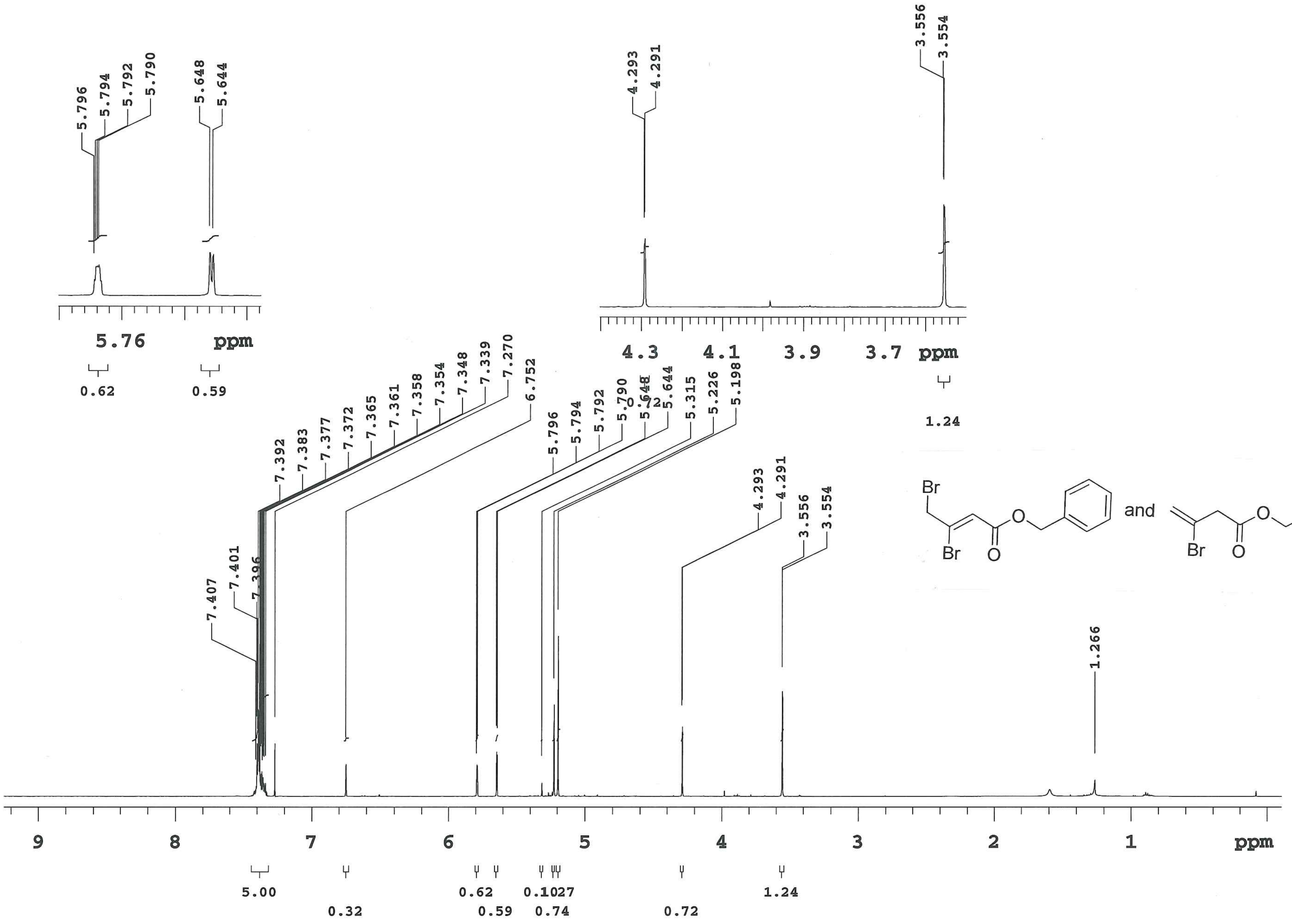

5.796
5.794
5.792
5.790
5.648
5.644
5.76
ppm
0.62
0.59
4.293
4.291
3.556
3.554
4.3
4.1
3.9
3.7
ppm
1.24
7.407
7.401
7.396
7.392
7.383
7.377
7.372
7.365
7.361
7.358
7.354
7.348
7.339
7.270
6.752
5.796
5.794
5.792
5.790
5.648
5.644
5.315
5.226
5.198
4.293
4.291
3.556
3.554
1.266
Br
Br
O
O
and
Br
O
O
9
8
7
6
5
4
3
2
1
ppm
5.00
0.32
0.62
0.59
0.1027
0.74
0.72
1.24

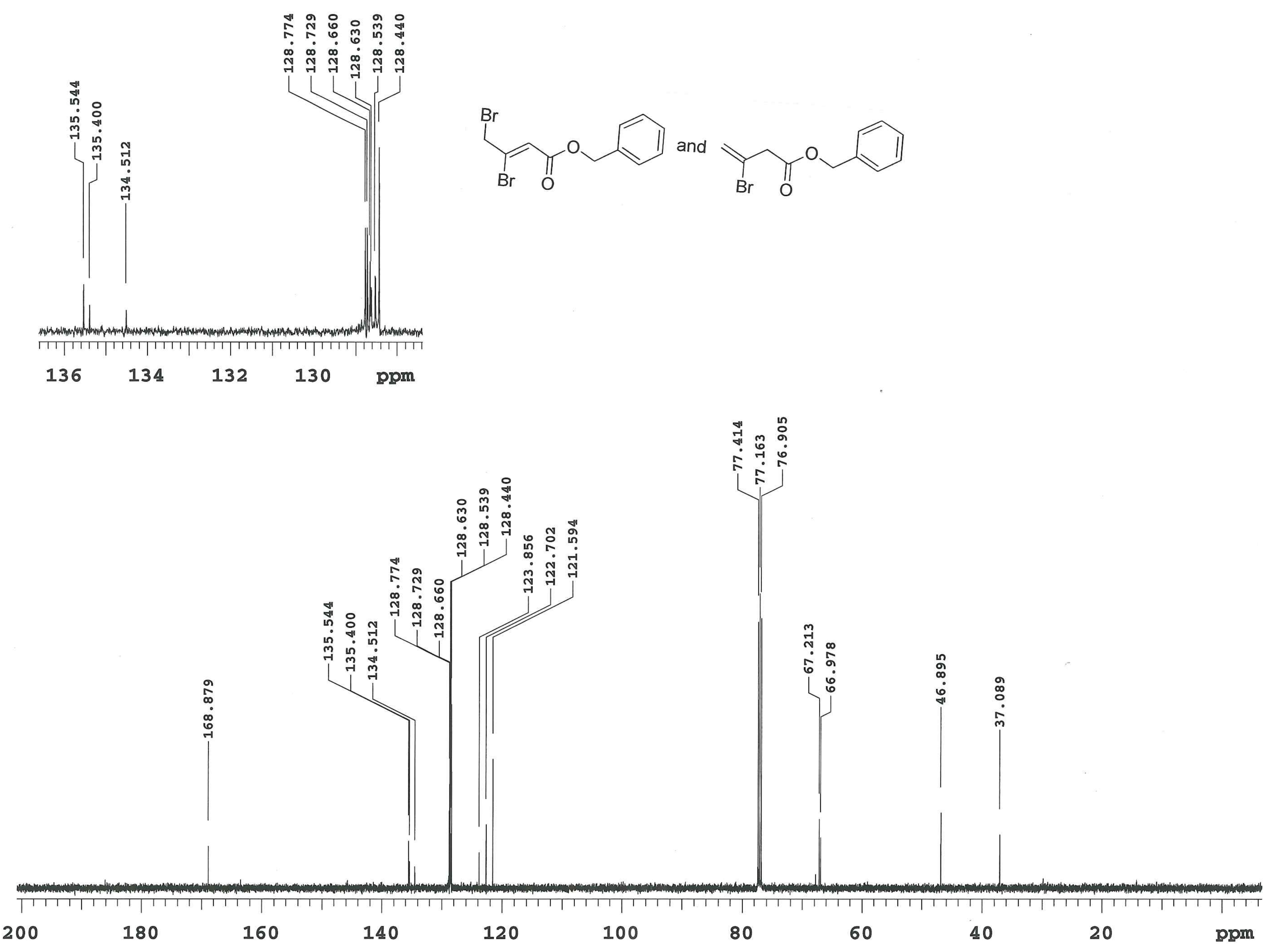
135.544
135.400
134.512
128.774
128.729
128.660
128.630
128.539
128.440
Br
Br
O
O
and
Br
O
O
136
134
132
130
ppm
168.879
135.544
135.400
134.512
128.774
128.729
128.660
128.630
128.539
128.440
123.856
122.702
121.594
77.414
77.163
76.905
67.213
66.978
46.895
37.089
200
180
160
140
120
100
80
60
40
20
ppm

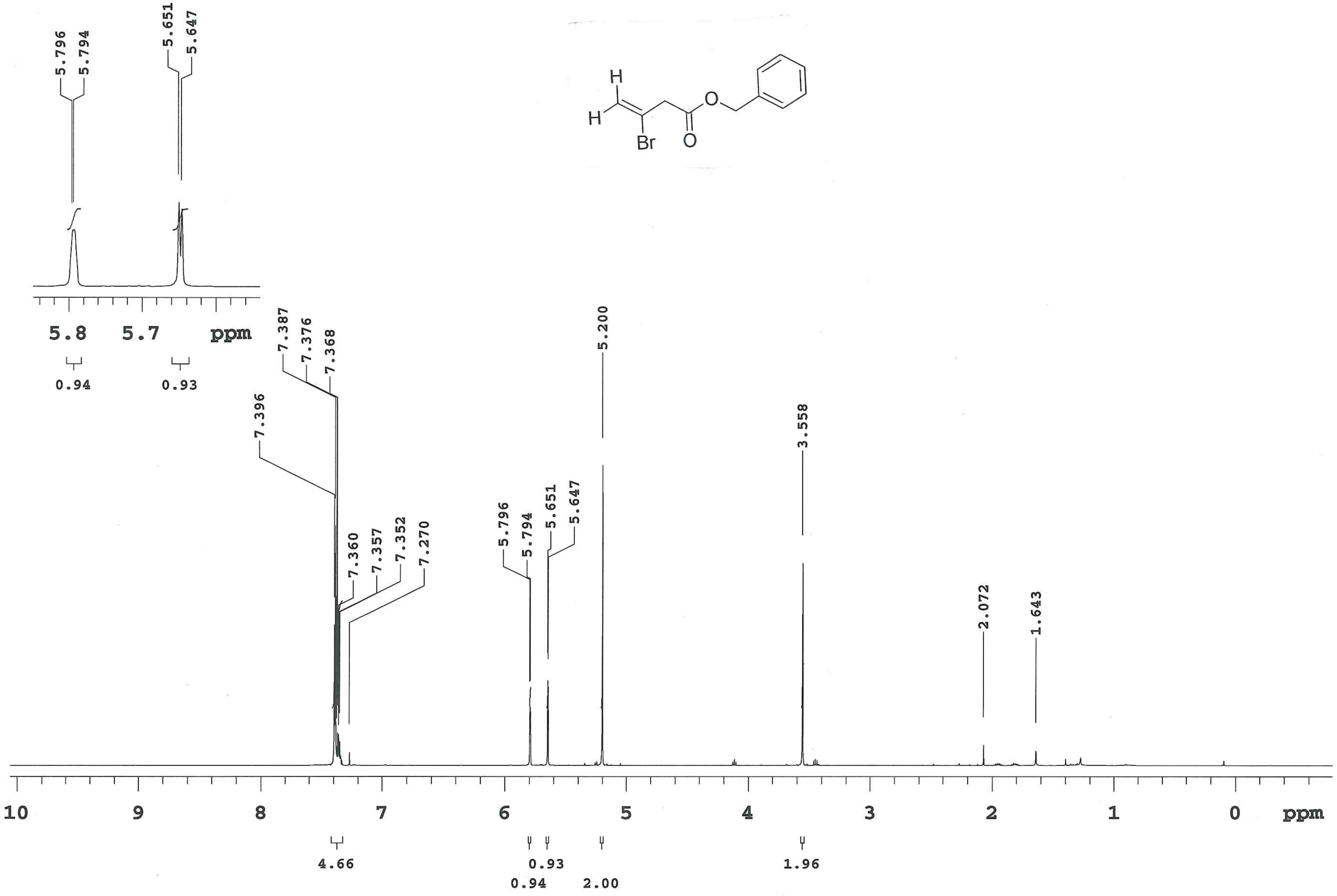
H
H
Br
O
O
5.796
5.794
5.651
5.647
5.8
5.7
ppm
0.94
0.93
7.387
7.376
7.368
7.396
7.360
7.357
7.352
7.270
5.796
5.794
5.651
5.647
5.200
3.558
2.072
1.643
10
9
8
7
6
5
4
3
2
1
0
ppm
4.66
0.93
0.94
2.00
1.96

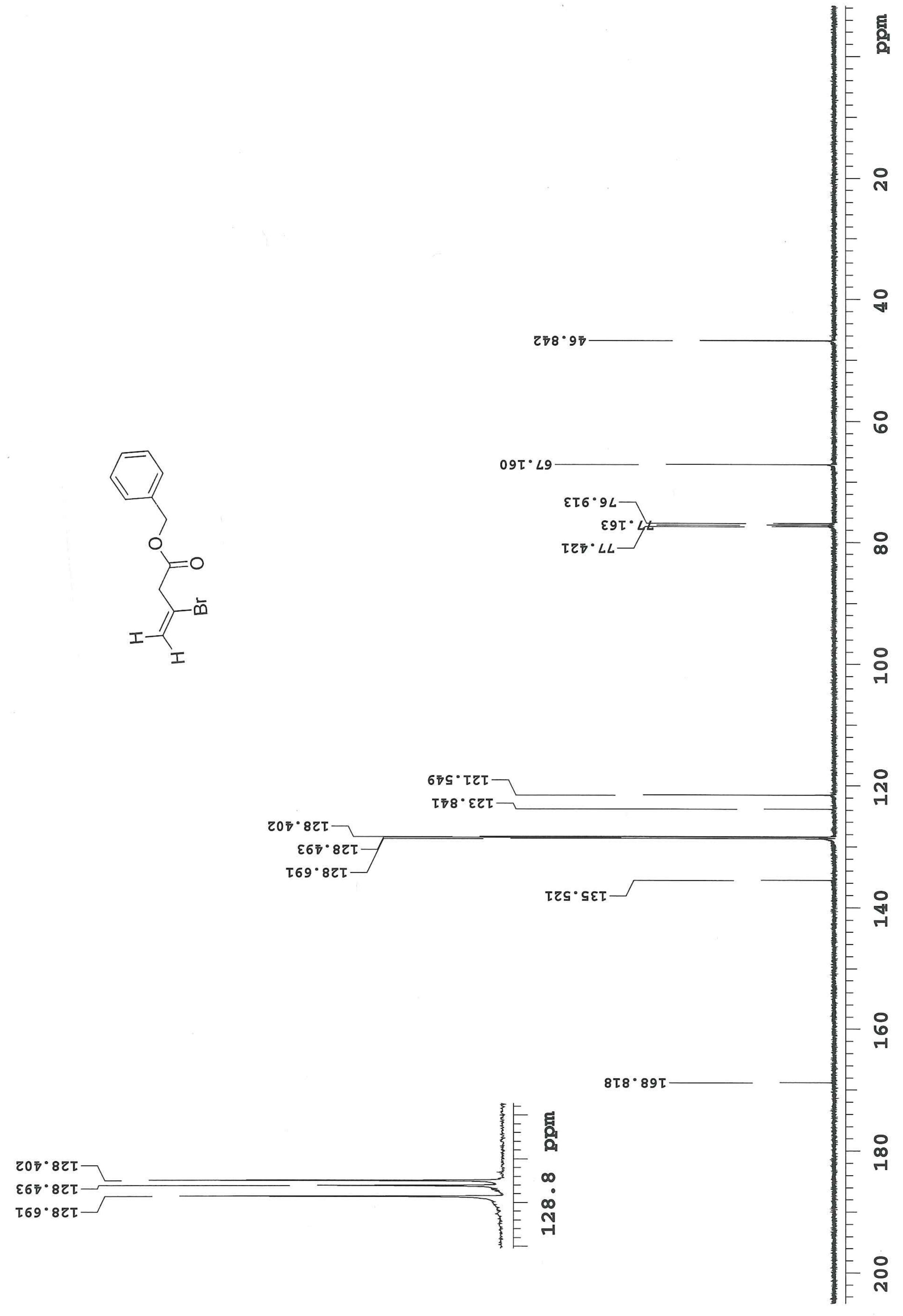
O
O
Br
H
H
46.842
67.160
76.913
77.163
77.421
121.549
123.841
128.402
128.493
128.691
135.521
168.818
20
40
60
80
100
120
140
160
180
200
ppm
128.402
128.493
128.691
128.8 ppm

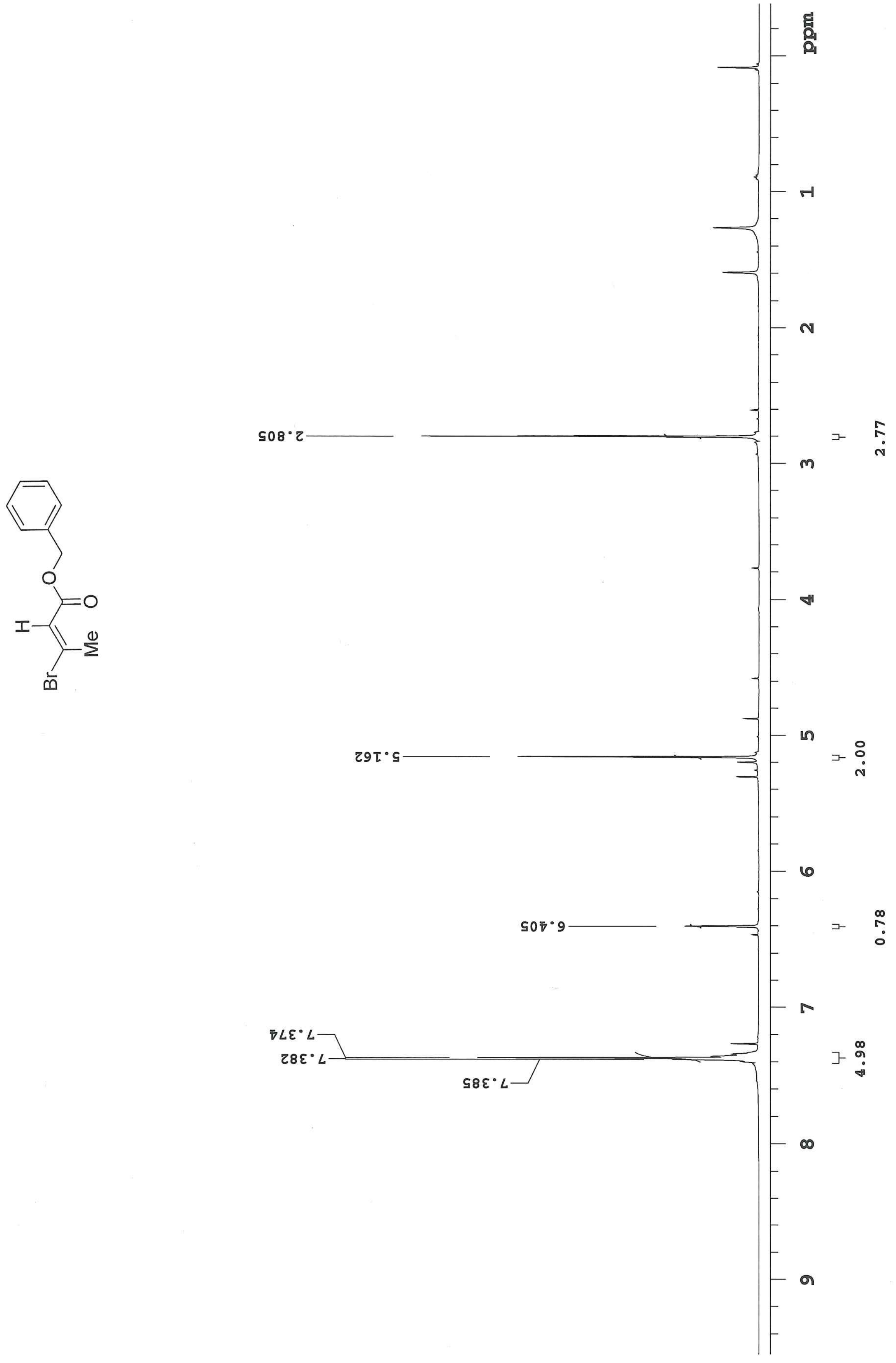

Br
H
Me
O
O
2.805
5.162
6.405
7.374
7.382
7.385
2.77
2.00
0.78
4.98
1
2
3
4
5
6
7
8
9
ppm

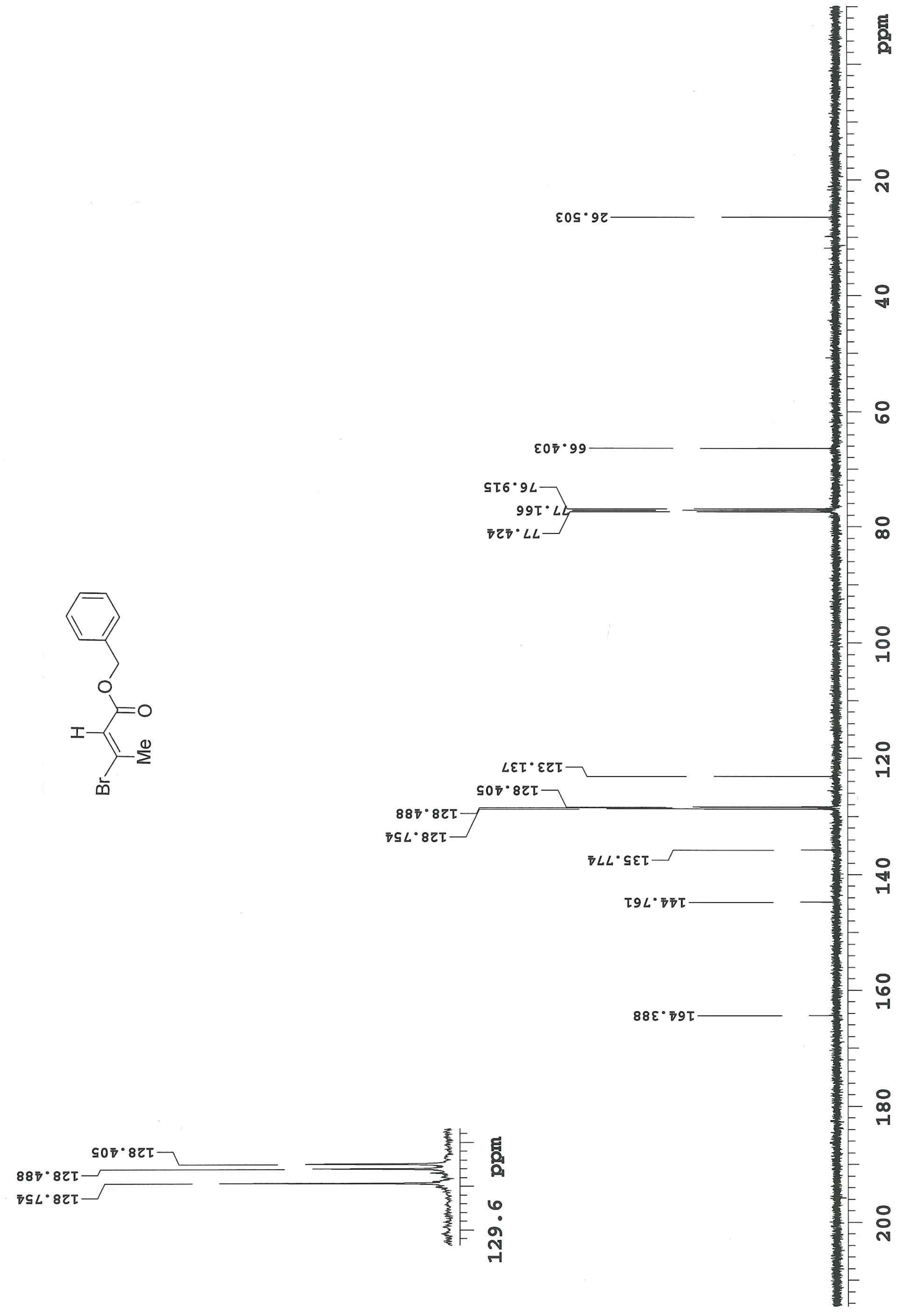

H
O
O
Br
Me
26.503
66.403
76.915
77.166
77.424
123.137
128.405
128.488
128.754
135.774
144.761
164.388
128.405
128.488
128.754
129.6 ppm
ppm
20
40
60
80
100
120
140
160
180
200

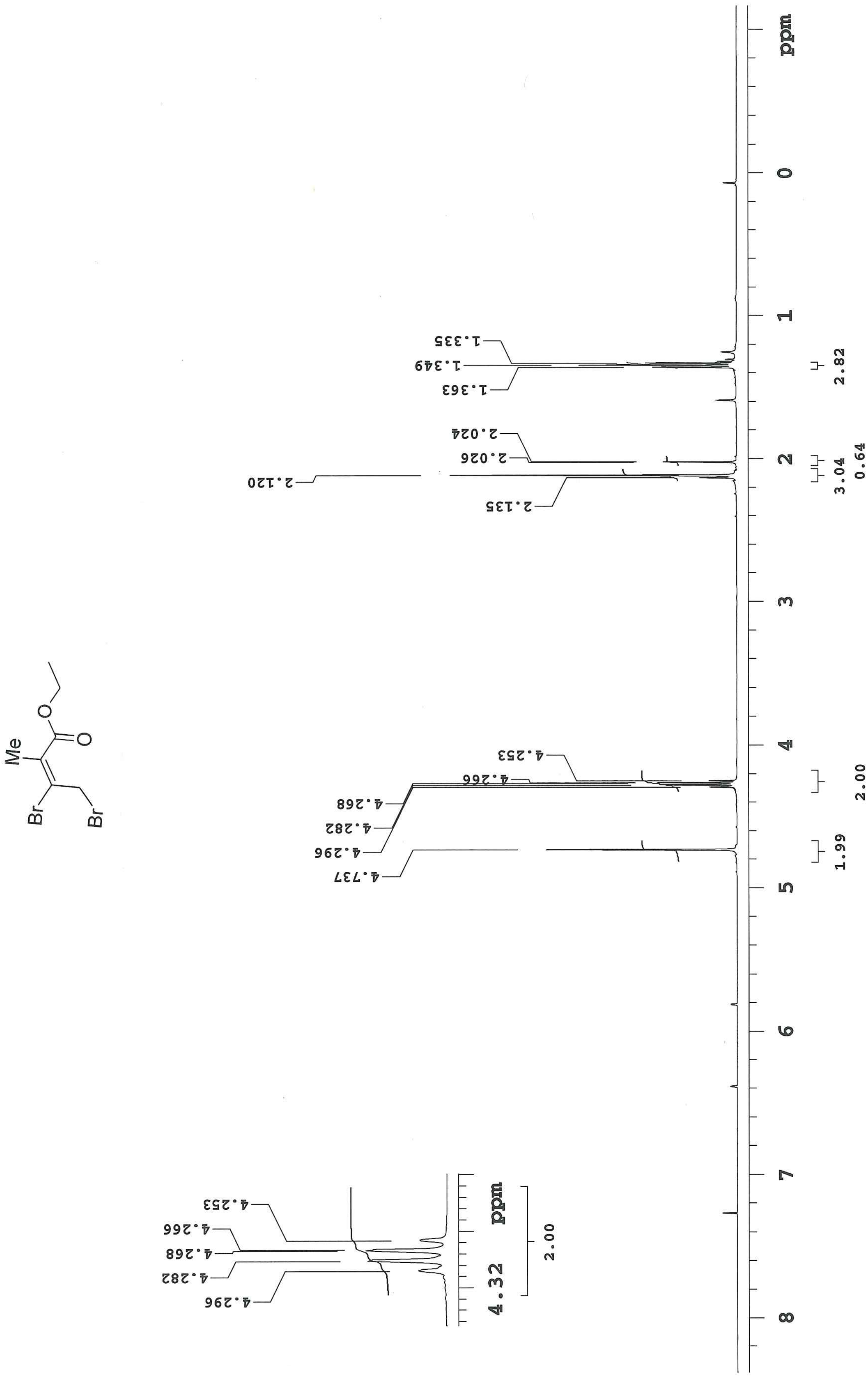

Me
O
O
Br
Br
ppm
0
1
2
3
4
5
6
7
8
1.335
1.349
1.363
2.024
2.026
2.120
2.135
4.253
4.266
4.268
4.282
4.296
4.737
2.82
3.04
0.64
2.00
1.99
4.32

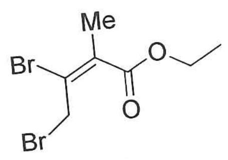
Me
Br
O
O
Br

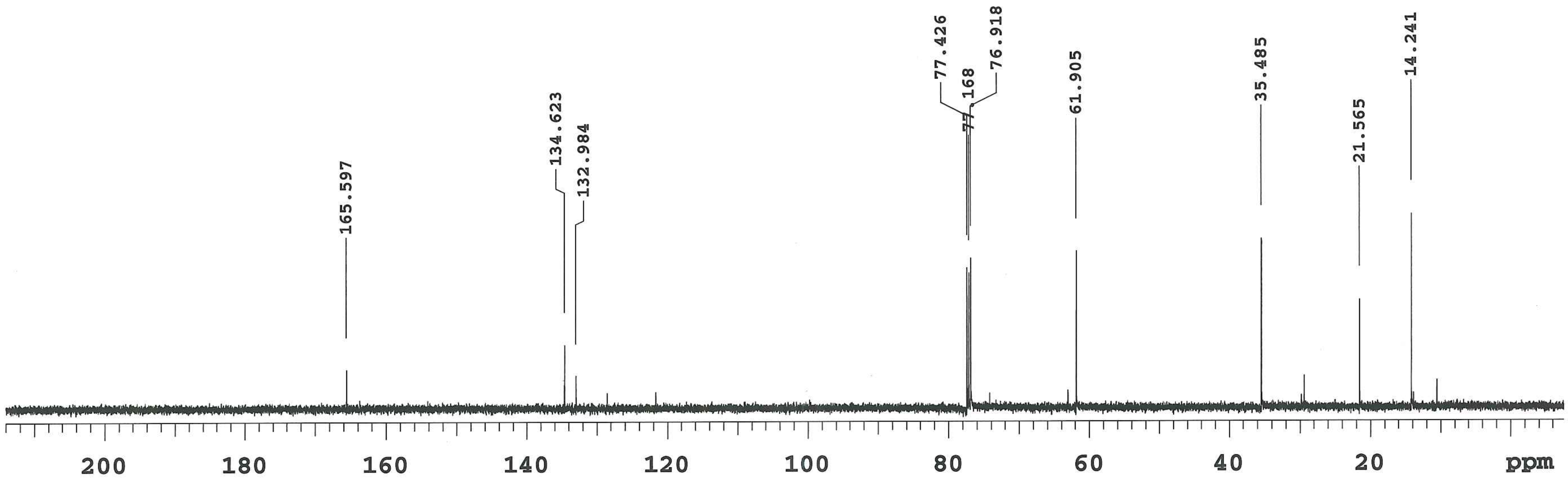
165.597
134.623
132.984
77.426
77.168
76.918
61.905
35.485
21.565
14.241
200
180
160
140
120
100
80
60
40
20
ppm

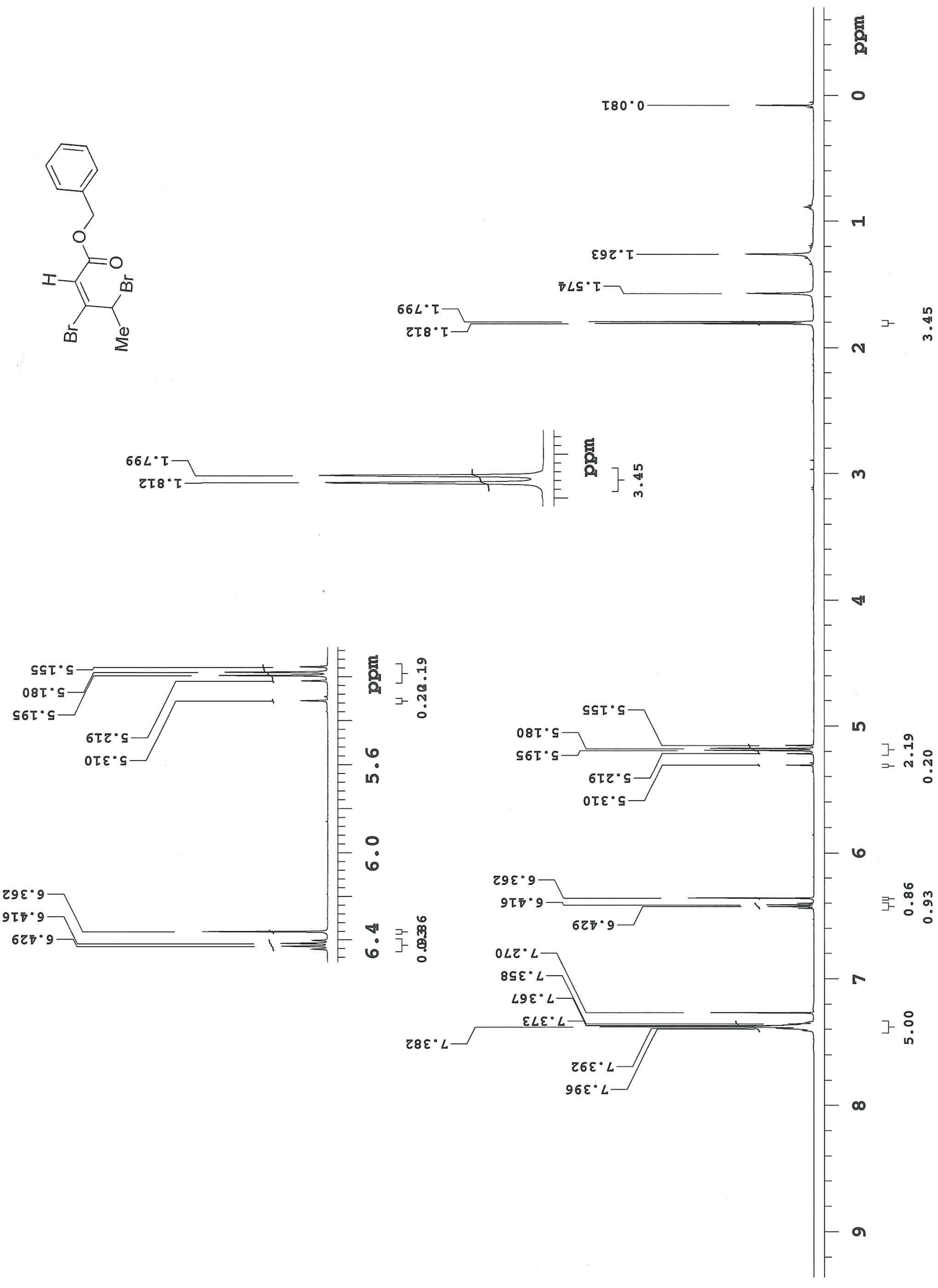
Br
H
O
O
Me
Br
0.081
1.263
1.574
1.799
1.812
5.155
5.180
5.195
5.219
5.310
6.362
6.416
6.429
7.270
7.358
7.367
7.373
7.382
7.392
7.396
3.45
2.19
0.20
0.86
0.93
5.00
ppm

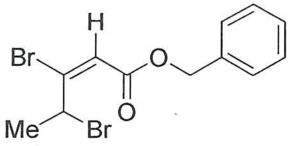

H
Br
O
Me
Br
O

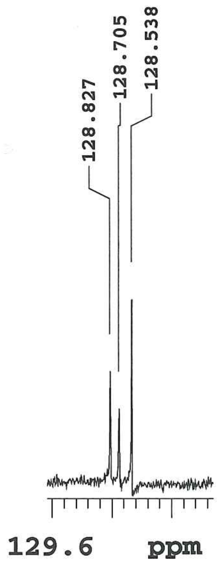

128.827
128.705
128.538
129.6
ppm

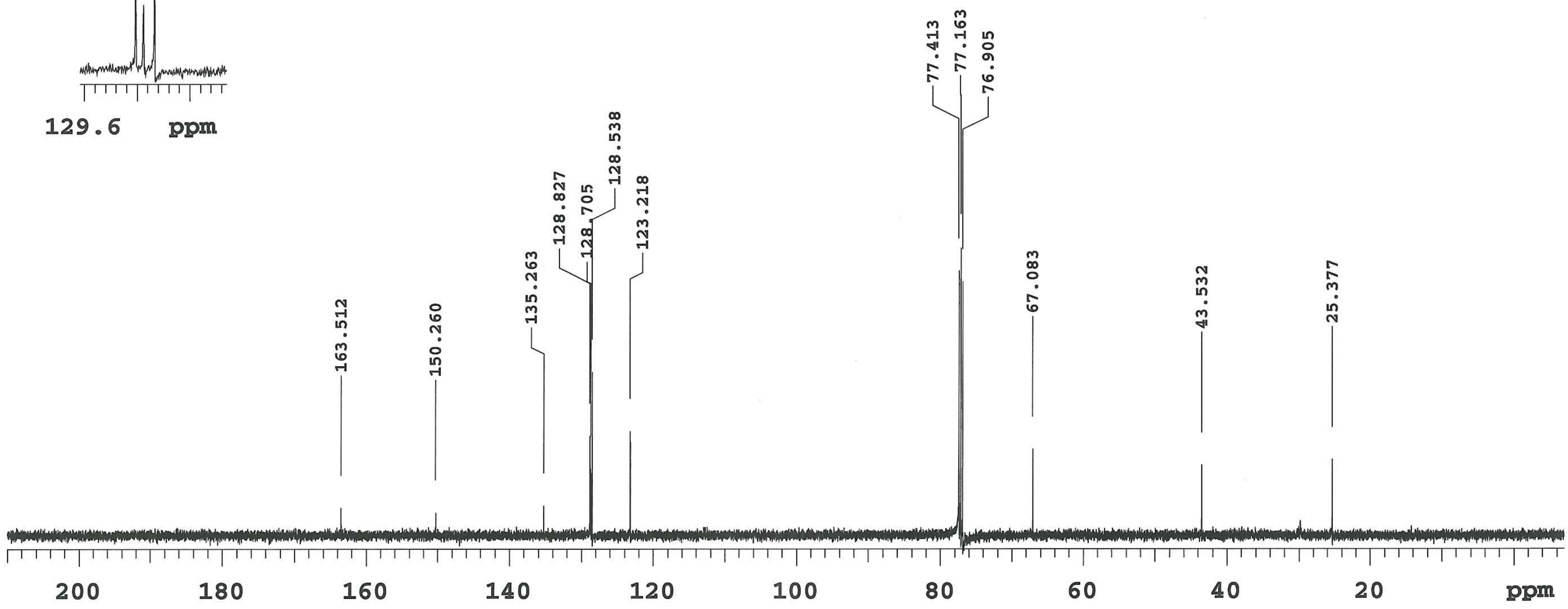

163.512
150.260
135.263
128.827
128.705
128.538
123.218
77.413
77.163
76.905
67.083
43.532
25.377
200
180
160
140
120
100
80
60
40
20
ppm

6g

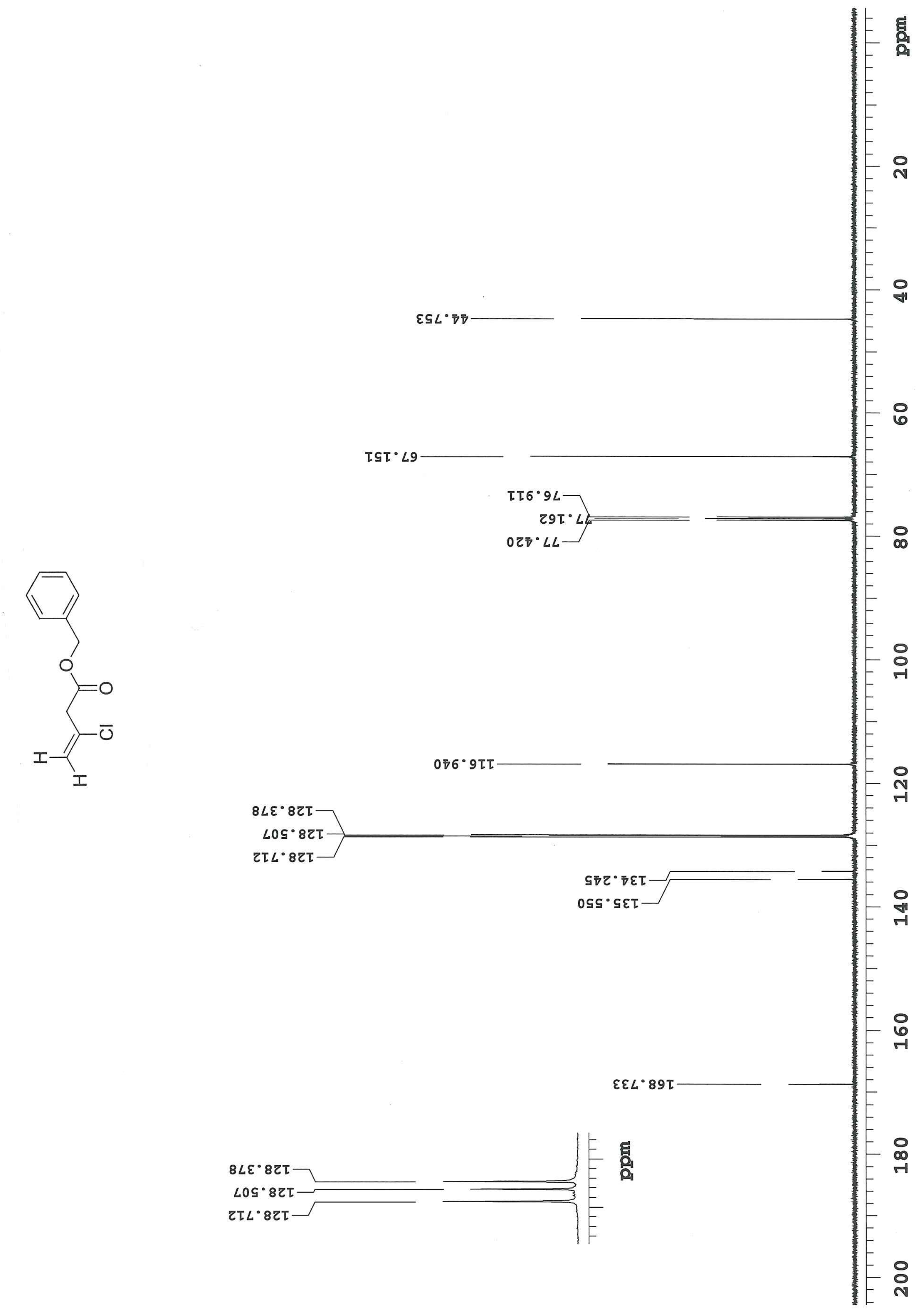
44.753
67.151
76.911
77.162
77.420
116.940
128.378
128.507
128.712
134.245
135.550
168.733
128.378
128.507
128.712
ppm
Cl
O
O
H
H
20
40
60
80
100
120
140
160
180
200
ppm

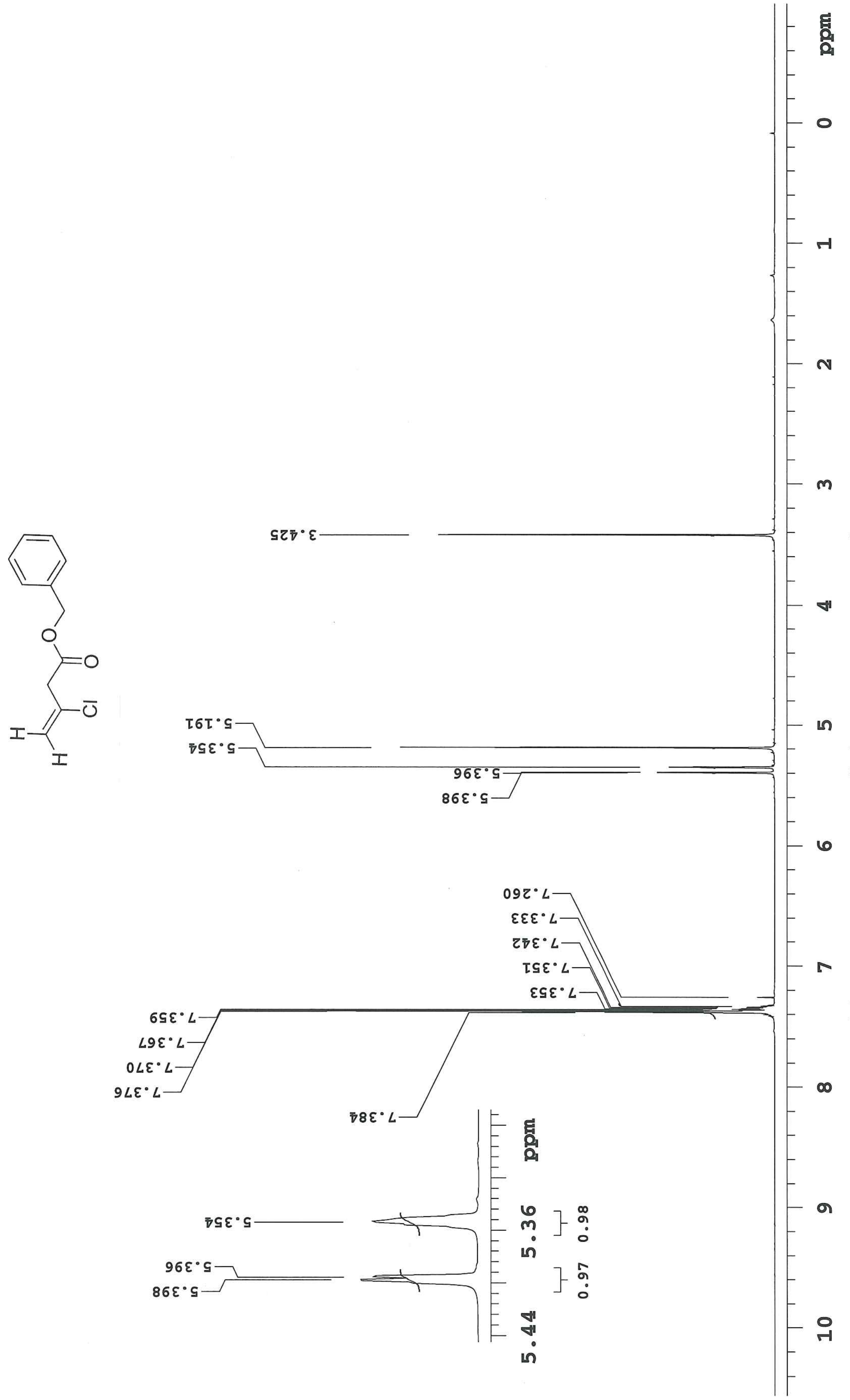

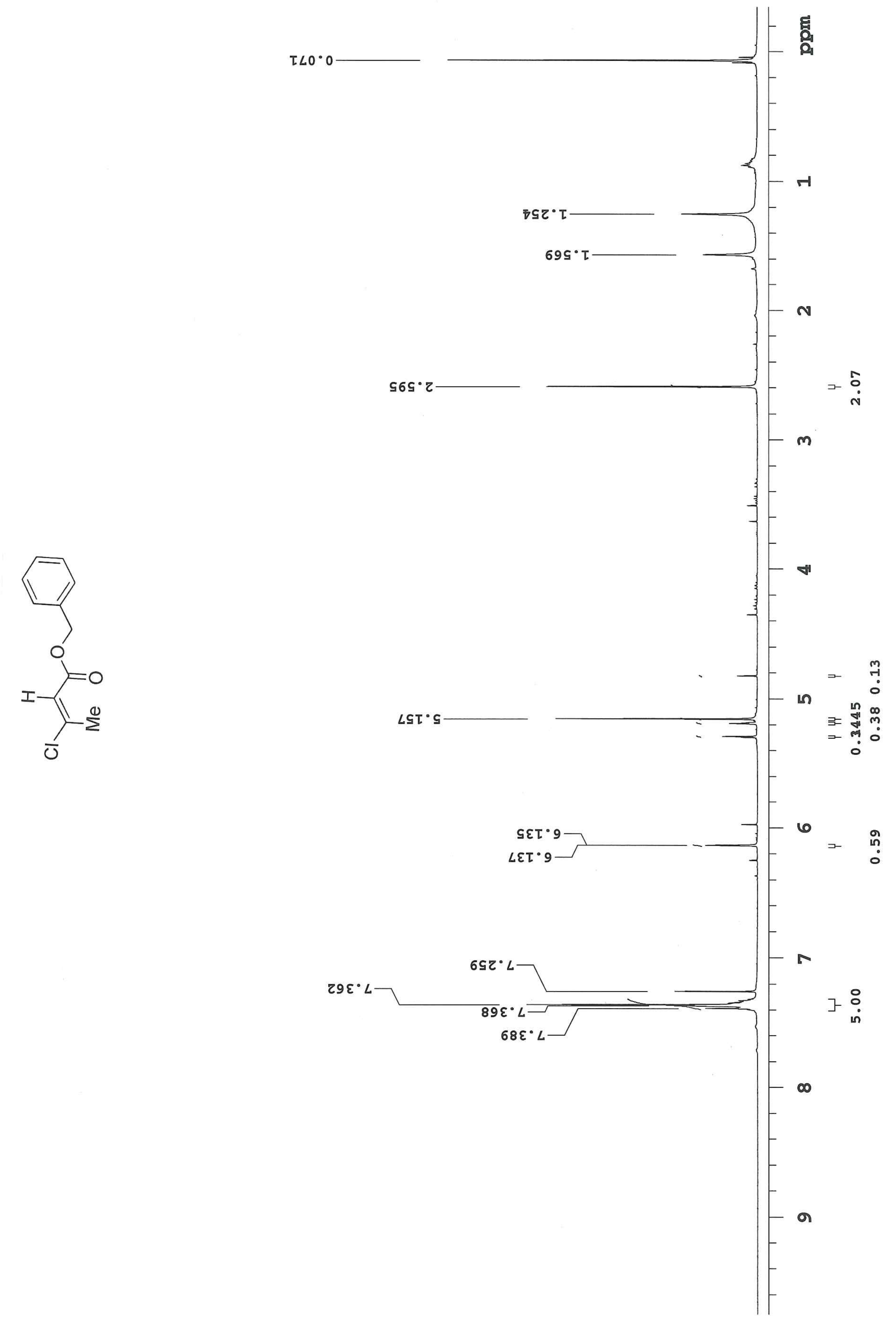
0.071
1.254
1.569
2.595
5.157
6.135
6.137
7.259
7.362
7.368
7.389
2.07
0.3445
0.38
0.13
0.59
5.00
ppm
1
2
3
4
5
6
7
8
9
H
O
Cl
Me

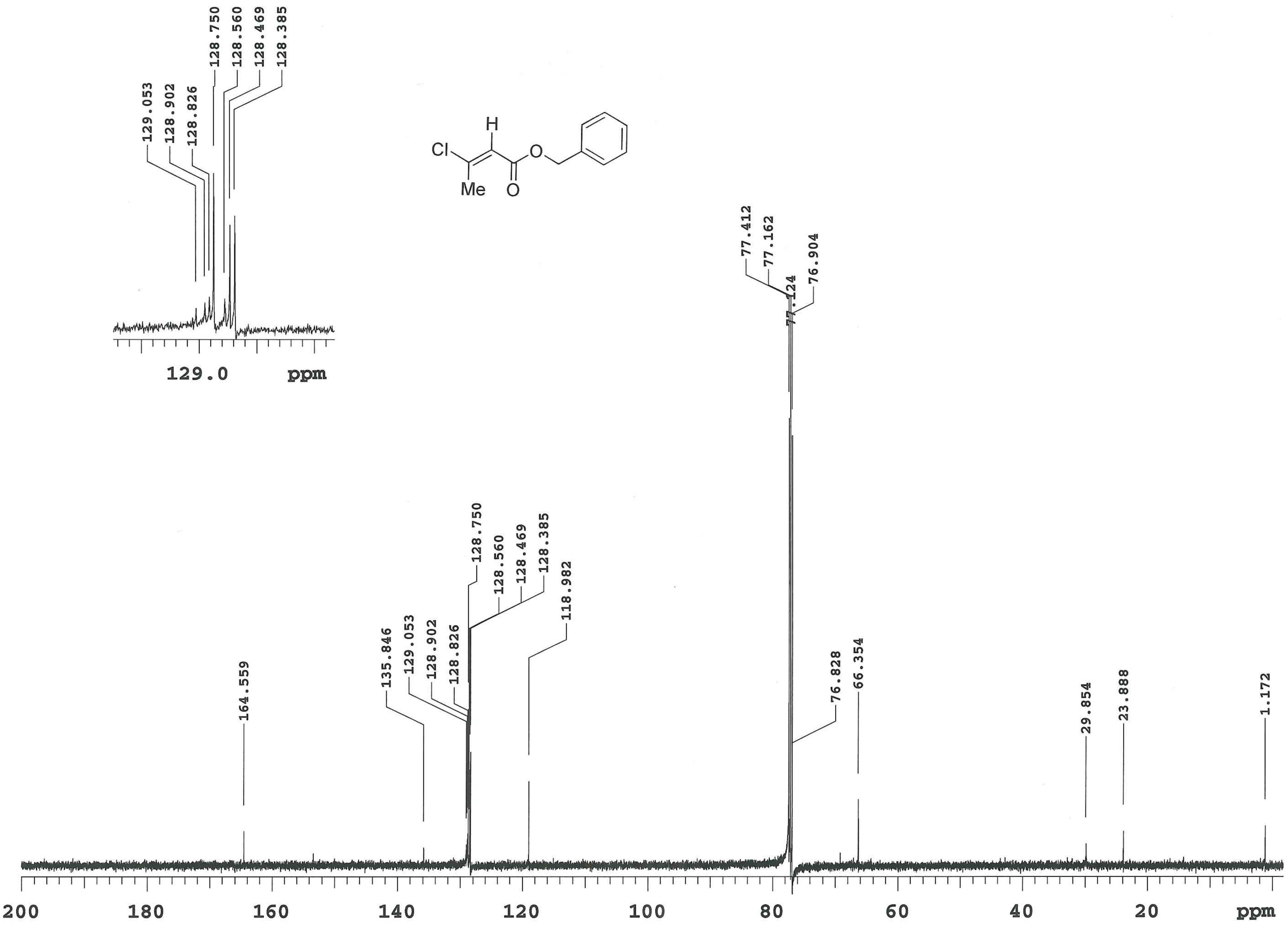
129.053
128.902
128.826
128.750
128.560
128.469
128.385
129.0
ppm
H
Cl
O
Me
O
77.412
77.162
77.124
76.904
164.559
135.846
129.053
128.902
128.826
128.750
128.560
128.469
128.385
118.982
76.828
66.354
29.854
23.888
1.172
200
180
160
140
120
100
80
60
40
20
ppm

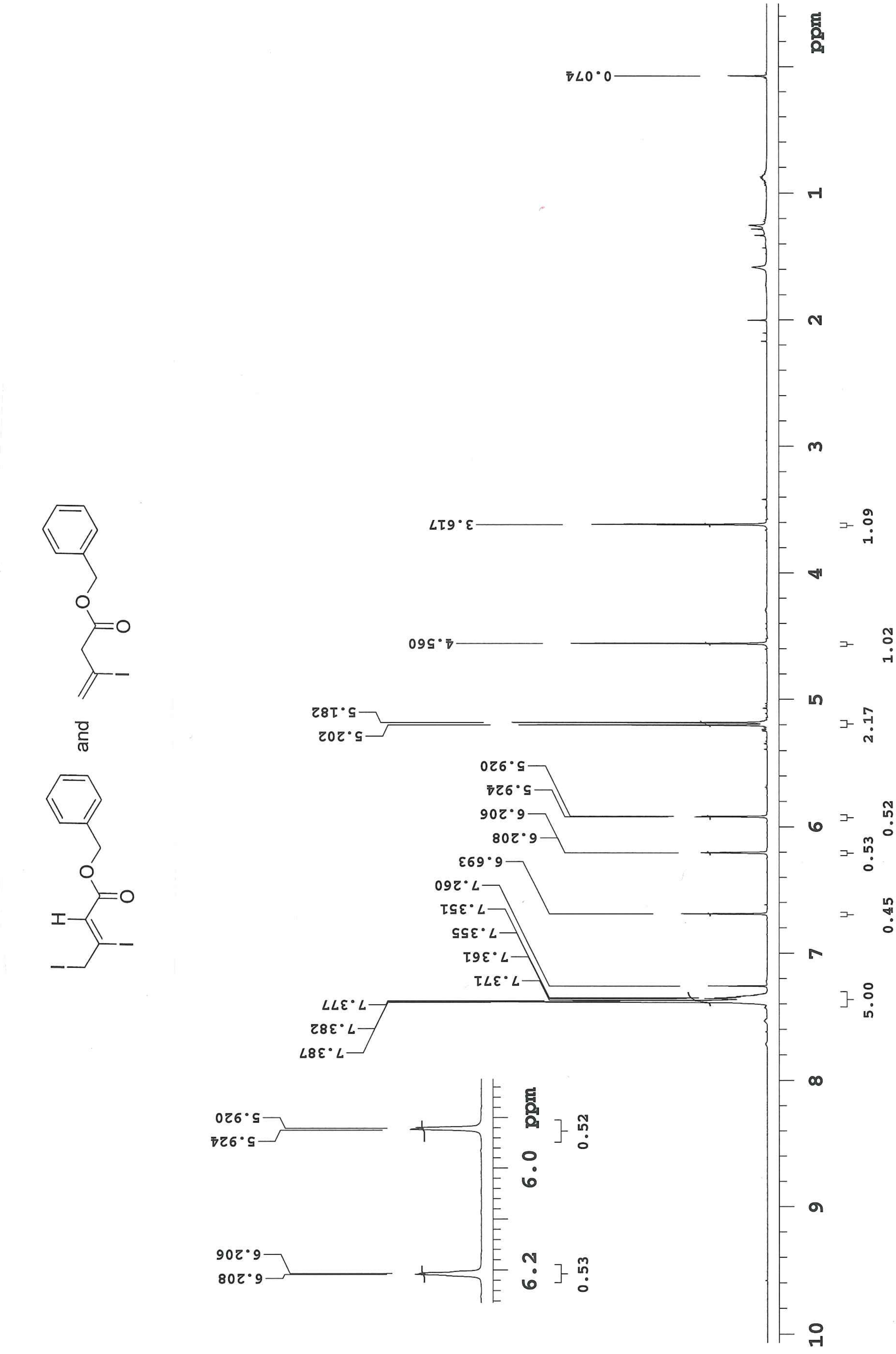
and
0.074
3.617
4.560
5.182
5.202
5.920
5.924
6.206
6.208
6.693
7.260
7.351
7.355
7.361
7.371
7.377
7.382
7.387
1.09
1.02
2.17
0.52
0.53
0.45
5.00
ppm
6.0
6.2

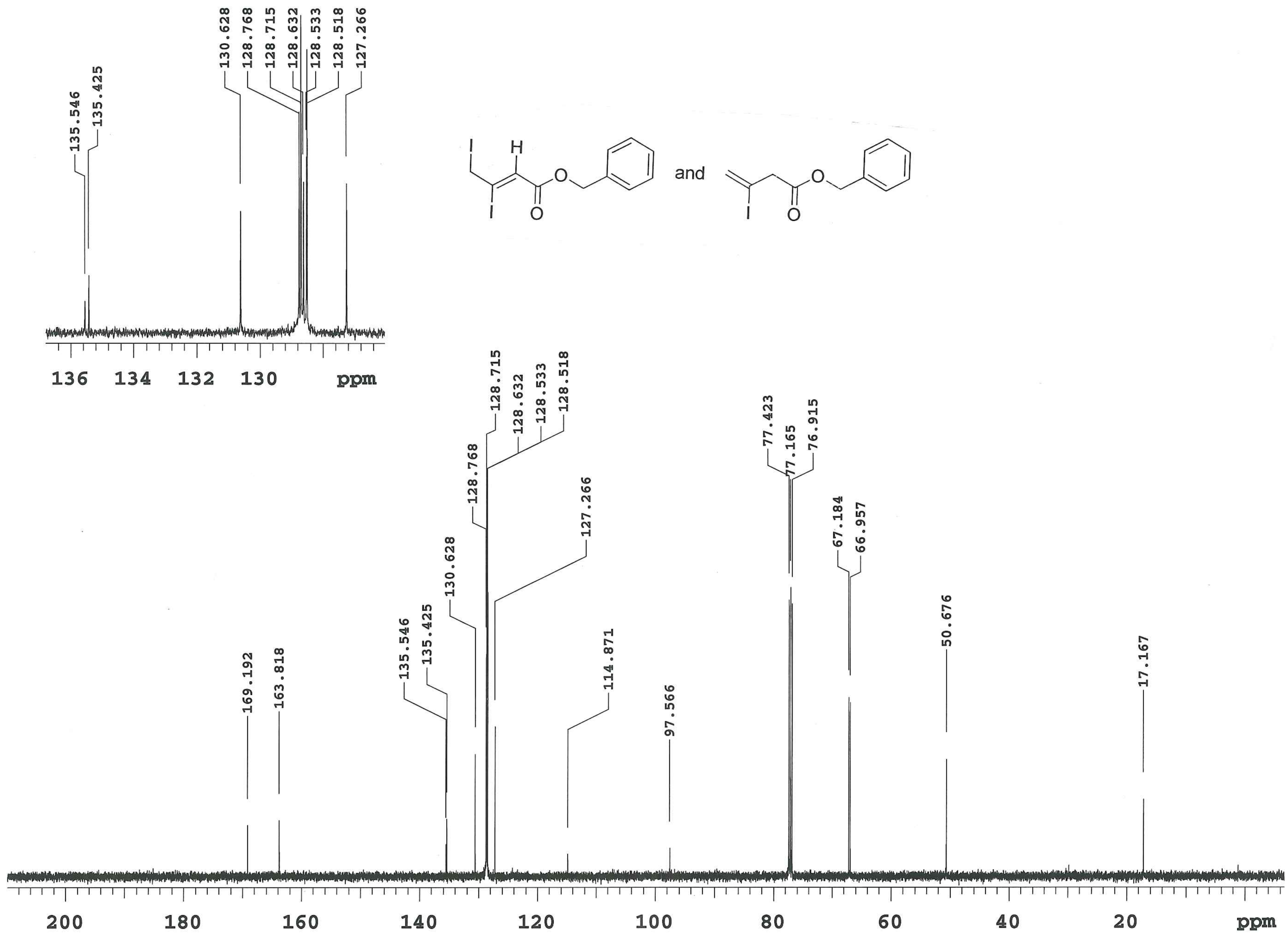

135.546
135.425
130.628
128.768
128.715
128.632
128.533
128.518
127.266
136
134
132
130
ppm
and
169.192
163.818
114.871
97.566
77.423
77.165
76.915
67.184
66.957
50.676
17.167
200
180
160
140
120
100
80
60
40
20

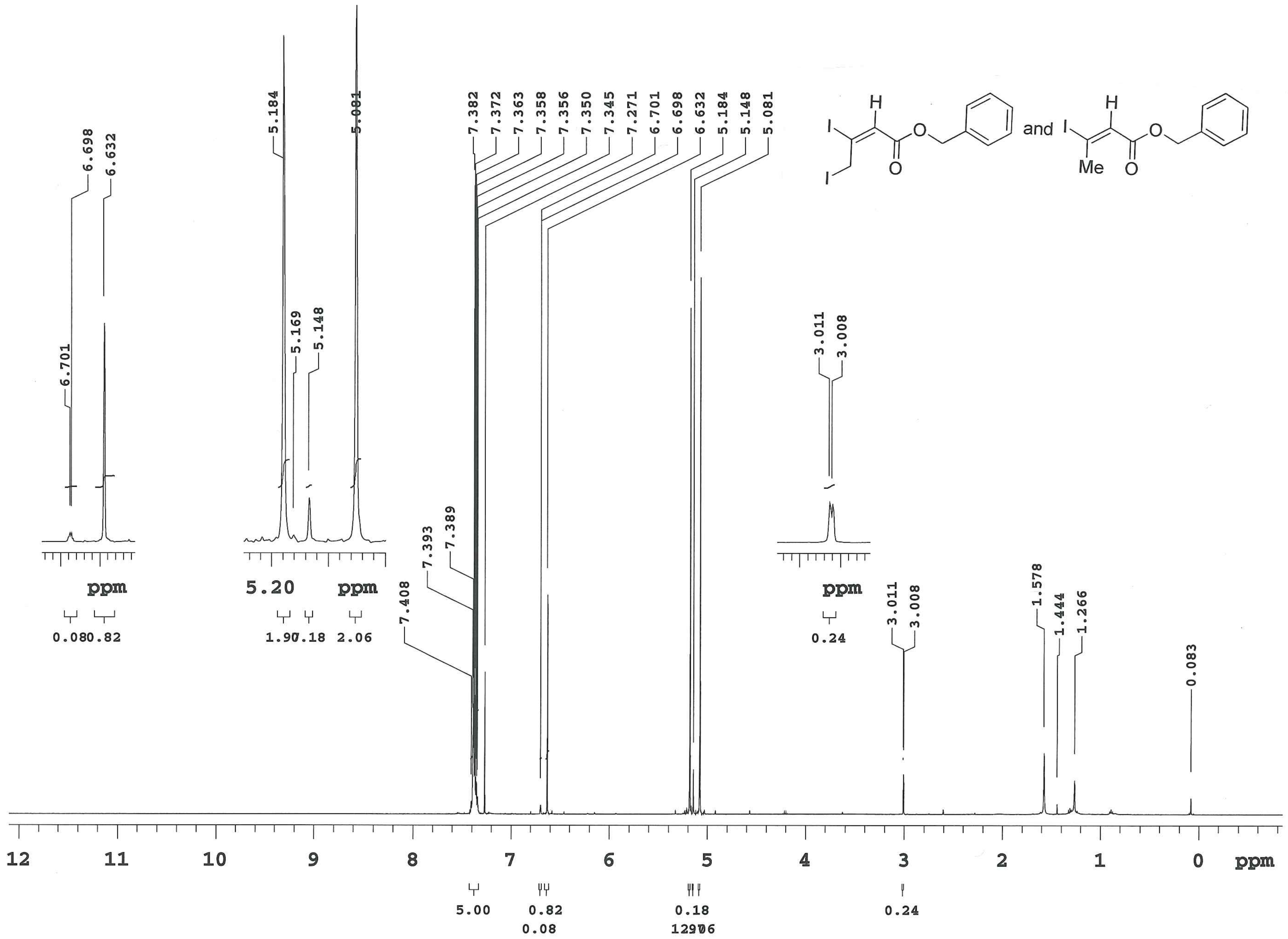

and
Me
H
I
O
6.698
6.632
6.701
ppm
0.080.82
5.184
5.169
5.148
5.081
5.20
ppm
1.907.18
2.06
7.382
7.372
7.363
7.358
7.356
7.350
7.345
7.271
6.701
6.698
6.632
5.184
5.148
5.081
7.408
7.393
7.389
3.011
3.008
ppm
0.24
3.011
3.008
1.578
1.444
1.266
0.083
12
11
10
9
8
7
6
5
4
3
2
1
0
ppm
5.00
0.82
0.08
0.18
12976
0.24

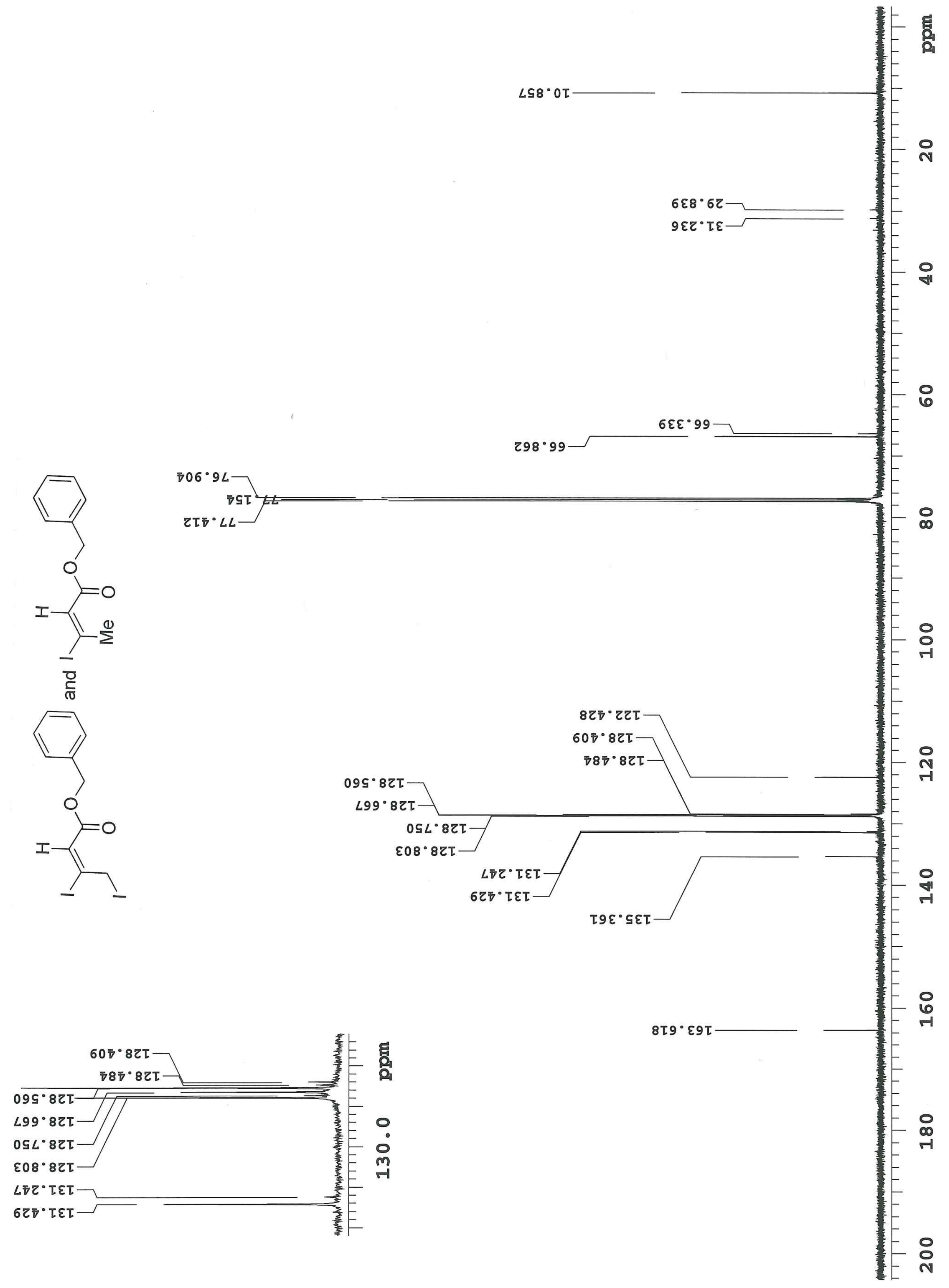
10.857
29.839
31.236
66.339
66.862
76.904
77.154
77.412
122.428
128.409
128.484
128.560
128.667
128.750
128.803
131.247
131.429
135.361
163.618
ppm
20
40
60
80
100
120
140
160
180
200
130.0
and
H
I
Me
O

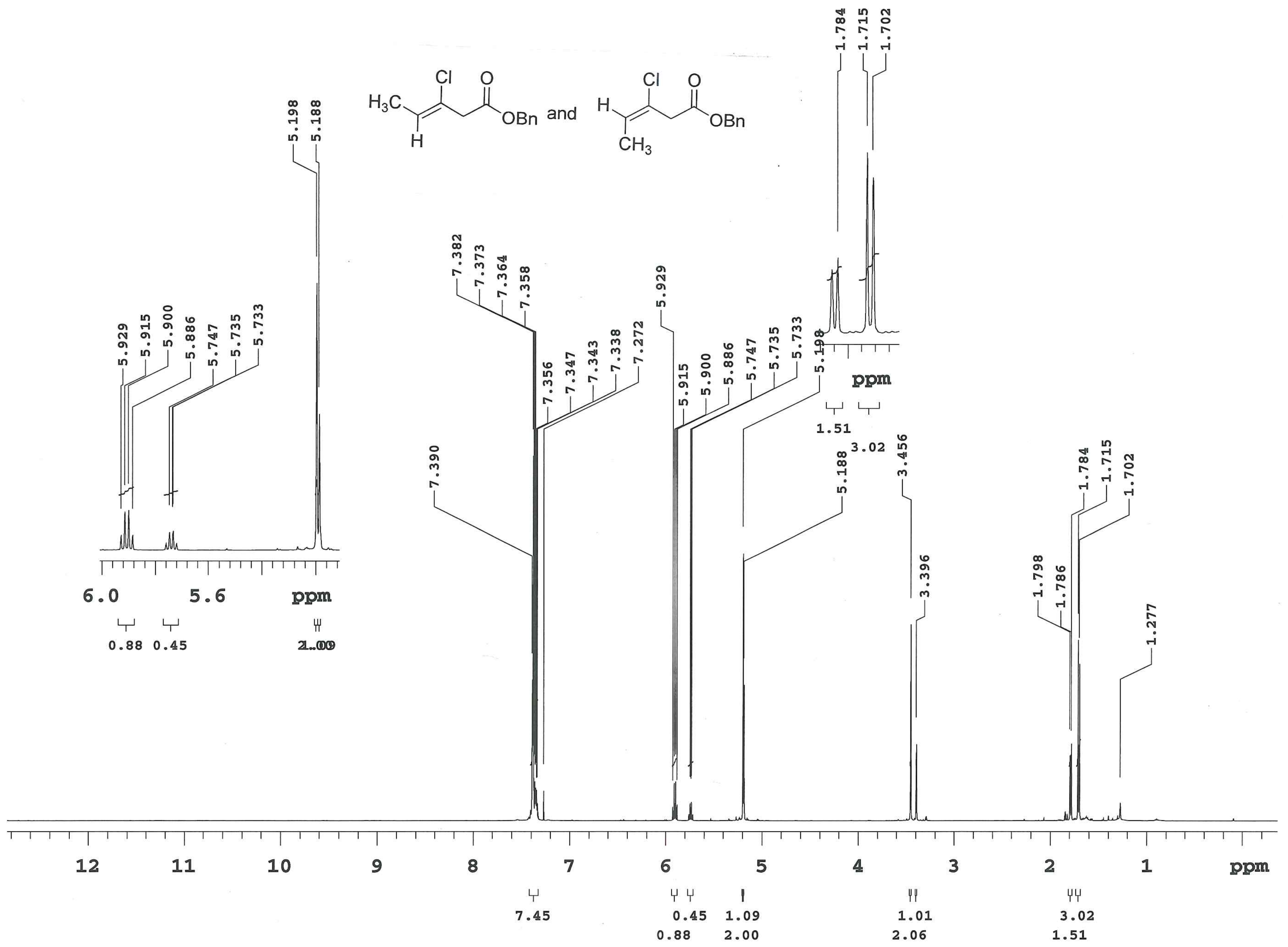

Cl
O
H3C
OBn
H
and
Cl
O
H
OBn
CH3
5.198
5.188
5.929
5.915
5.900
5.886
5.747
5.735
5.733
6.0
5.6
ppm
0.88 0.45
2.00
1.09
7.382
7.373
7.364
7.358
7.390
7.356
7.347
7.343
7.338
7.272
5.929
5.915
5.900
5.886
5.747
5.735
5.733
5.198
5.188
1.784
1.715
1.702
ppm
1.51
3.02
3.456
3.396
1.798
1.786
1.784
1.715
1.702
1.277
12
11
10
9
8
7
6
5
4
3
2
1
ppm
7.45
0.45
0.88
1.09
2.00
1.01
2.06
3.02
1.51

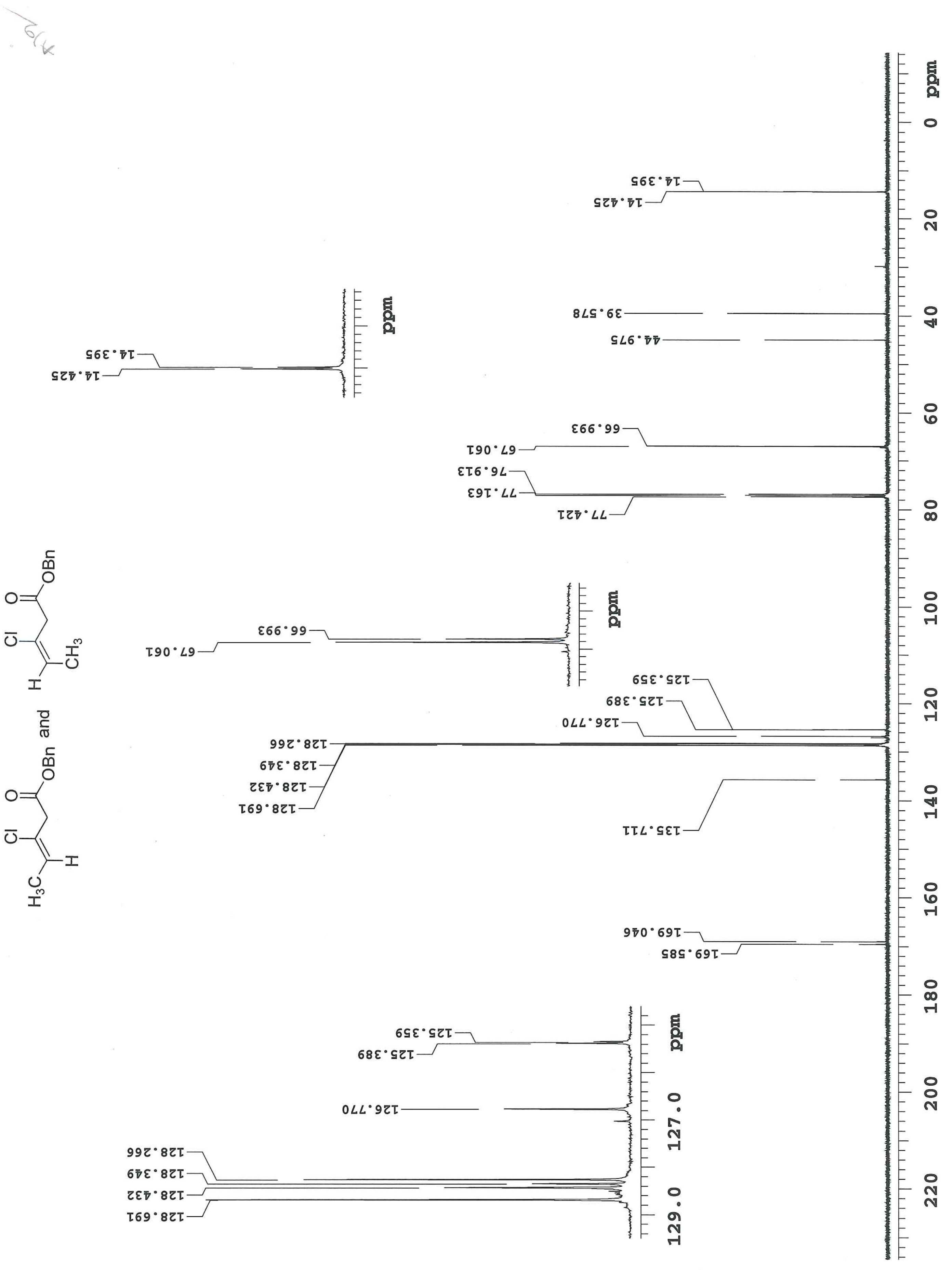

Cl
O
OBn
H$_3$C
H
and
Cl
O
OBn
H
CH$_3$
169.585
169.046
135.711
128.691
128.432
128.349
128.266
126.770
125.389
125.359
77.421
77.163
76.913
67.061
66.993
44.975
39.578
14.425
14.395
ppm
220
200
180
160
140
120
100
80
60
40
20
0
129.0
127.0

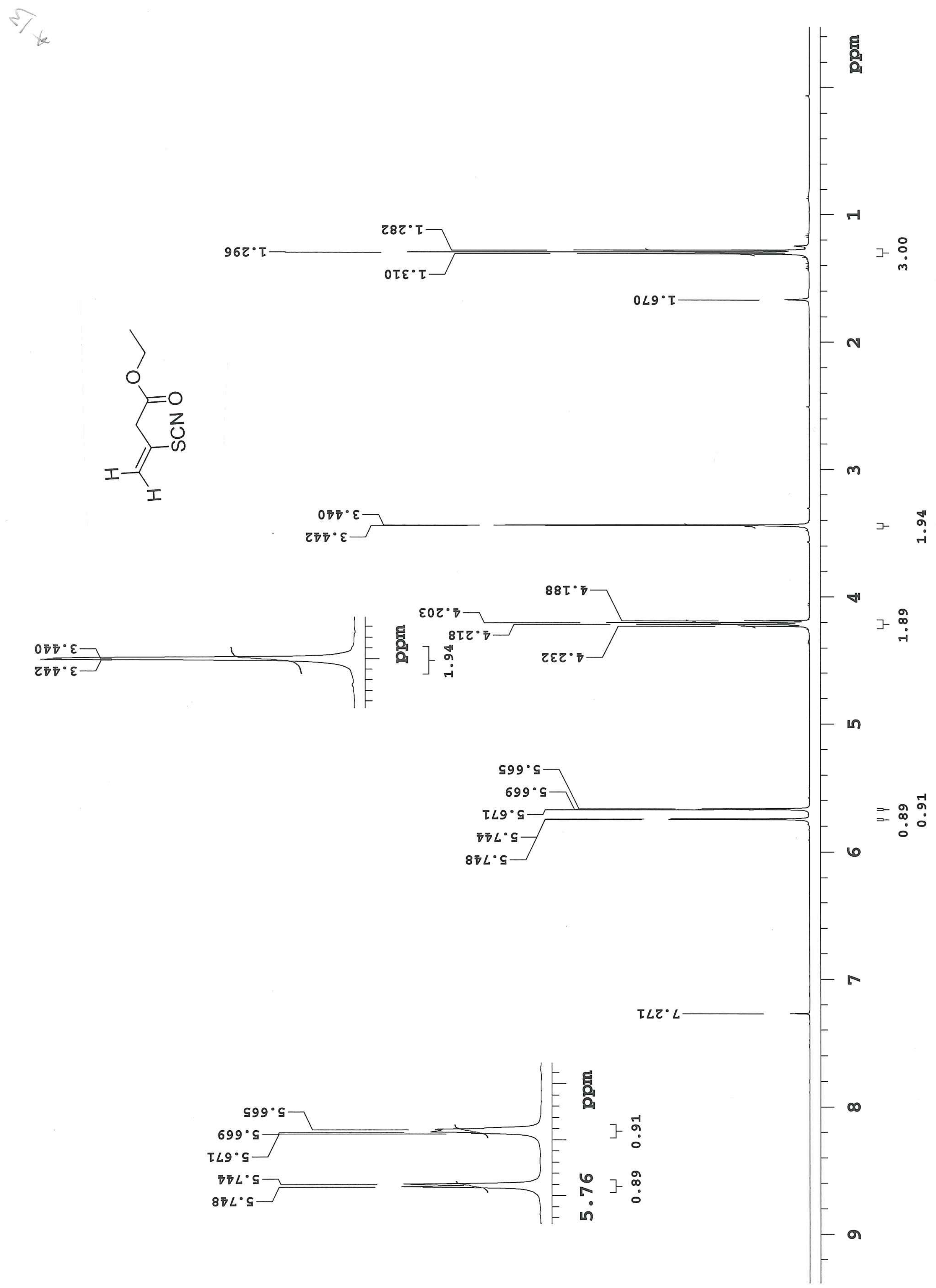
1.296
1.282
1.310
1.670
3.440
3.442
4.188
4.203
4.218
4.232
5.665
5.669
5.671
5.744
5.748
7.271
3.00
1.94
1.89
0.89
0.91
5.76
ppm
SCN
O
H

A13

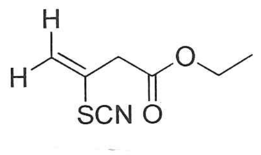
H
H
SCN
O
O

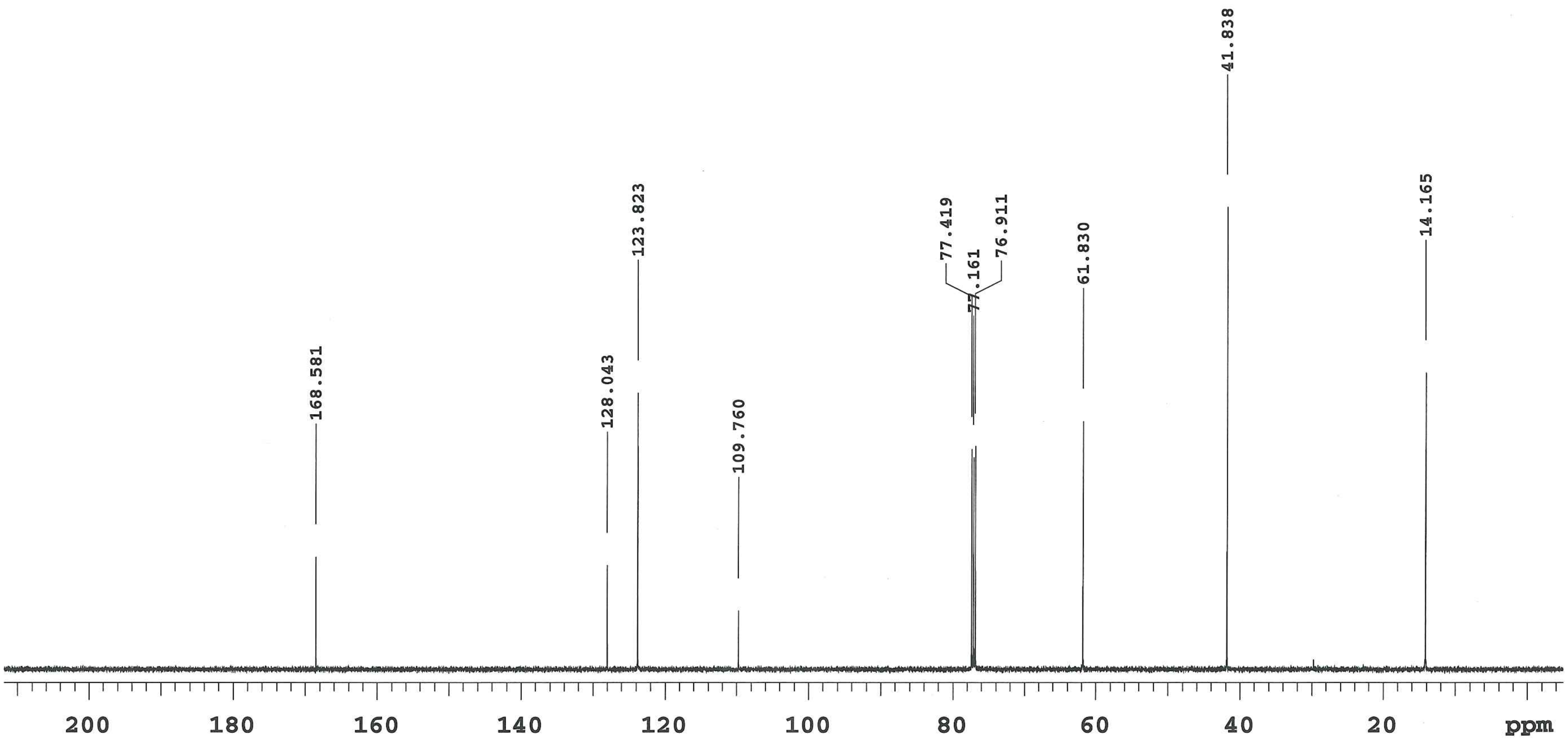
168.581
128.043
123.823
109.760
77.419
77.161
76.911
61.830
41.838
14.165
200
180
160
140
120
100
80
60
40
20
ppm

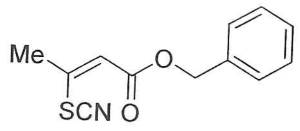
Me
SCN
O
O

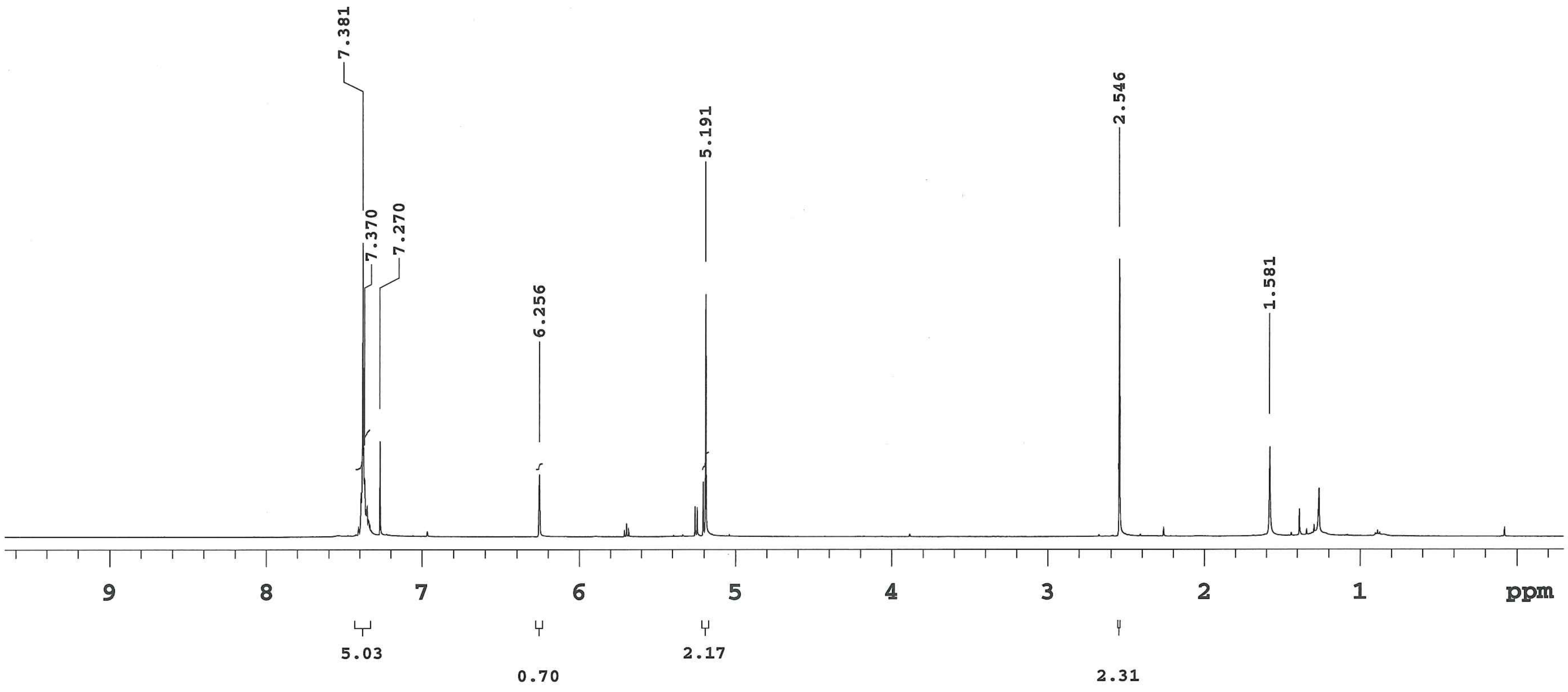
7.381
7.370
7.270
6.256
5.191
2.546
1.581
9
8
7
6
5
4
3
2
1
ppm
5.03
0.70
2.17
2.31

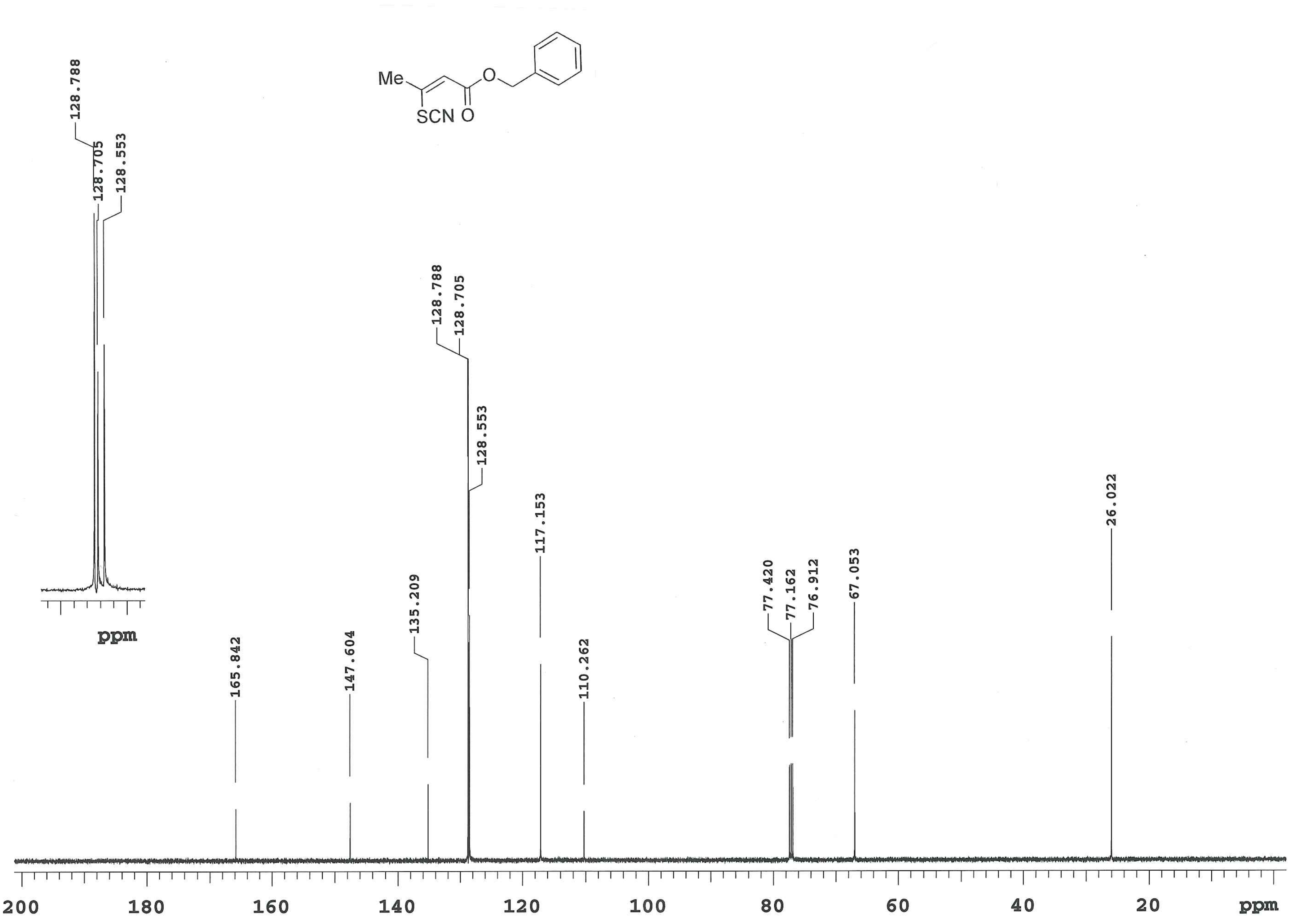
Me
SCN
O
O
128.788
128.705
128.553
ppm
165.842
147.604
135.209
128.788
128.705
128.553
117.153
110.262
77.420
77.162
76.912
67.053
26.022
200
180
160
140
120
100
80
60
40
20
ppm

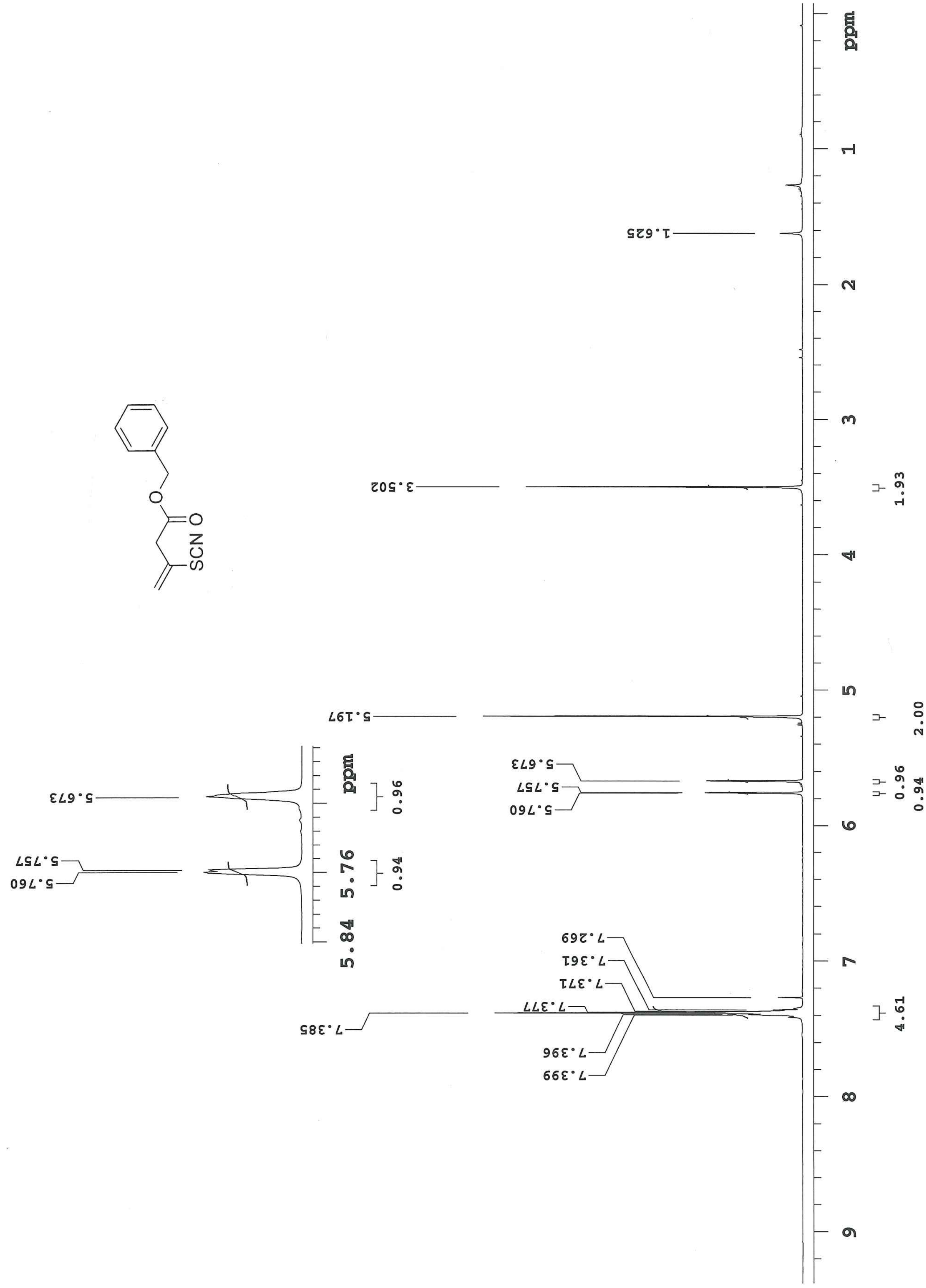
SCN O
O
1.625
3.502
5.197
5.673
5.757
5.760
7.269
7.361
7.371
7.377
7.385
7.396
7.399
1.93
2.00
0.96
0.94
4.61
5.84 5.76
ppm

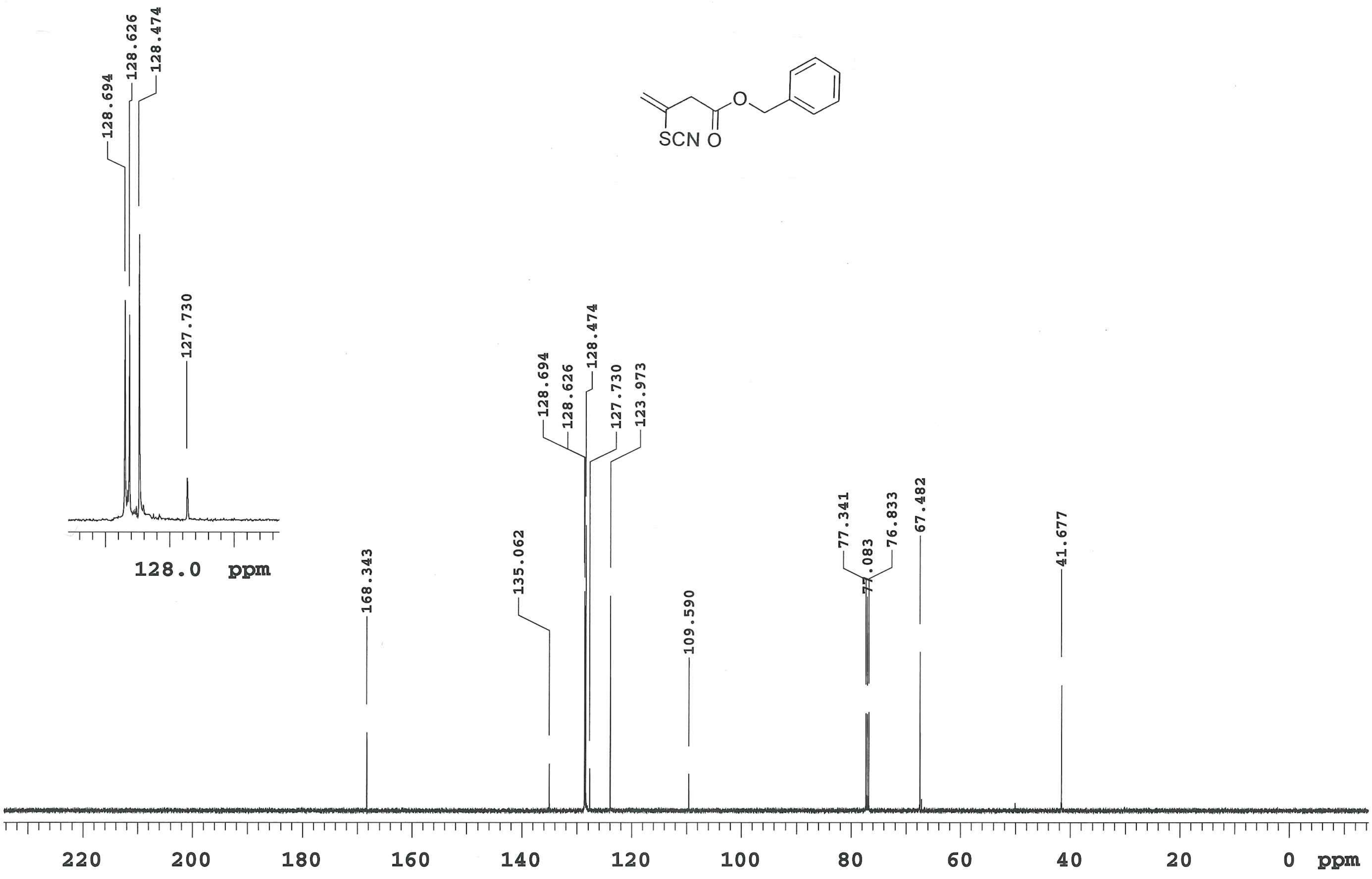
SCN O
128.694
128.626
128.474
127.730
128.0 ppm
168.343
135.062
128.694
128.626
128.474
127.730
123.973
109.590
77.341
77.083
76.833
67.482
41.677
220 200 180 160 140 120 100 80 60 40 20 0 ppm

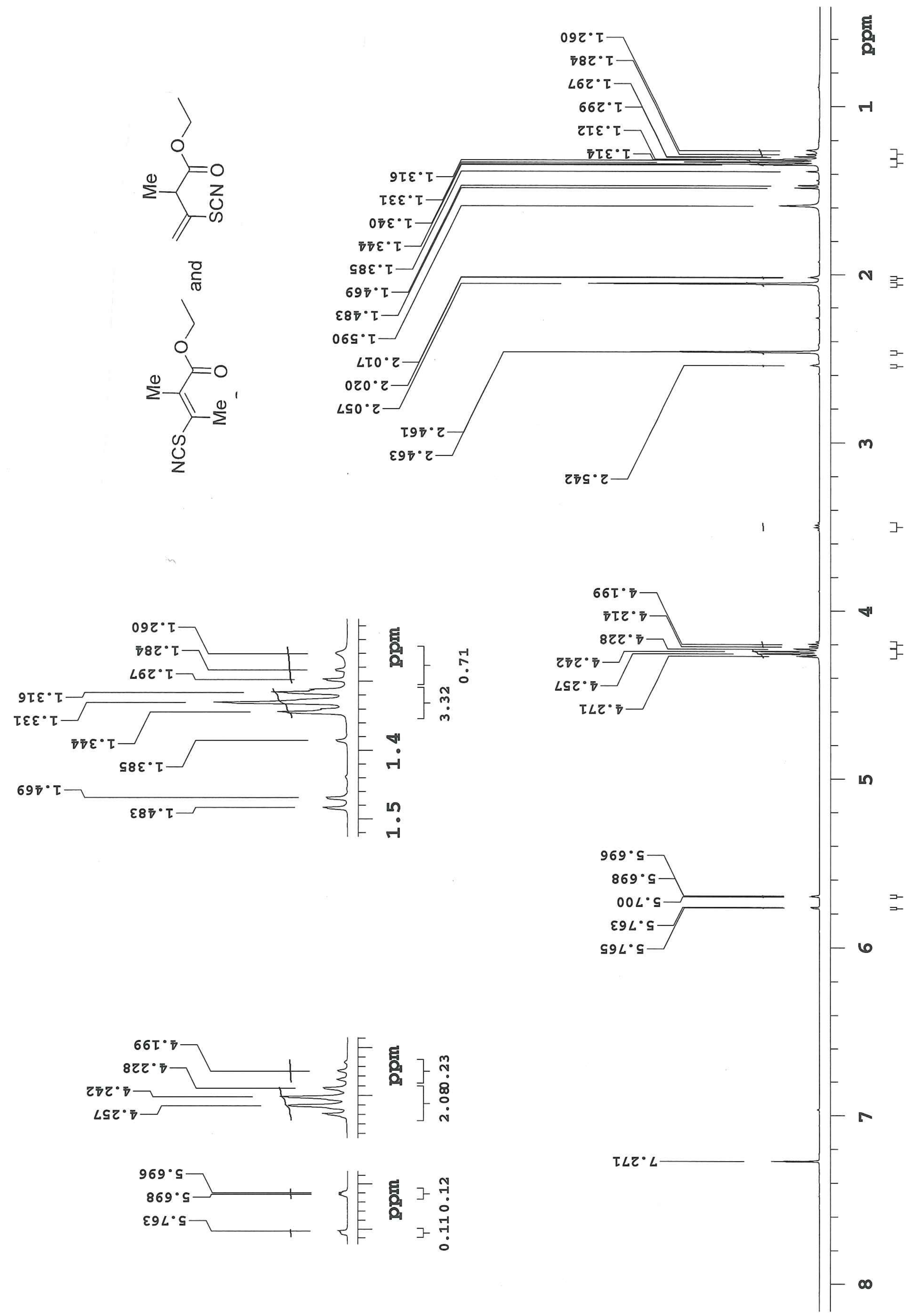
NCS
Me
Me
O
O
and
Me
SCN
O
O
ppm
1.260
1.284
1.297
1.299
1.312
1.314
1.316
1.331
1.340
1.344
1.385
1.469
1.483
1.590
2.017
2.020
2.057
2.461
2.463
2.542
4.199
4.214
4.228
4.242
4.257
4.271
5.696
5.698
5.700
5.763
5.765
7.271
0.71
3.32
0.18
2.99
2.32
0.13
0.08
2.08
0.23
0.11
0.12
1.4
1.5
2.080.23
0.110.12

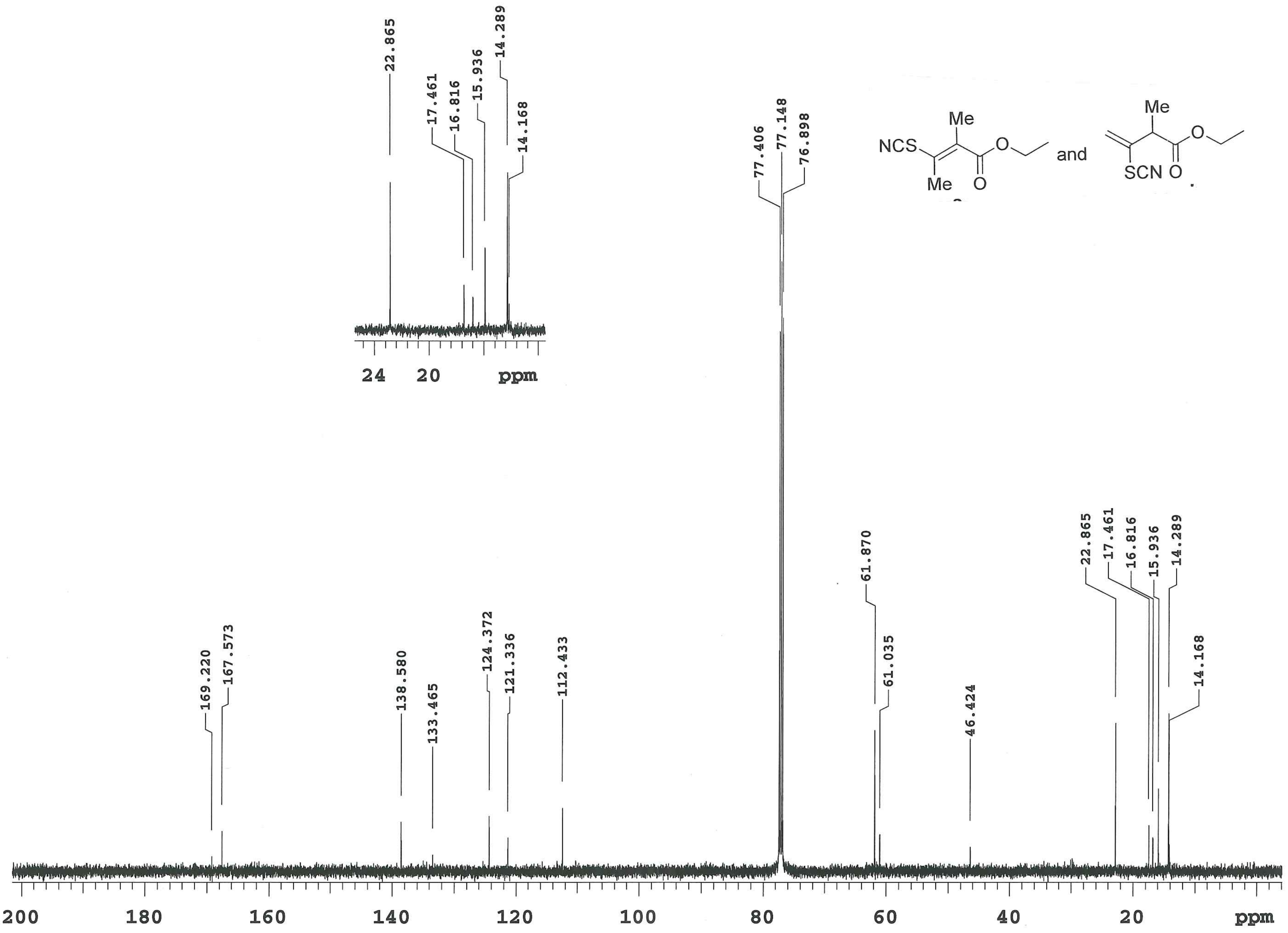

22.865
17.461
16.816
15.936
14.289
14.168
24
20
ppm
77.406
77.148
76.898
NCS
Me
Me
O
O
and
Me
SCN
O
O
169.220
167.573
138.580
133.465
124.372
121.336
112.433
61.870
61.035
46.424
200
180
160
140
120
100
80
60
40

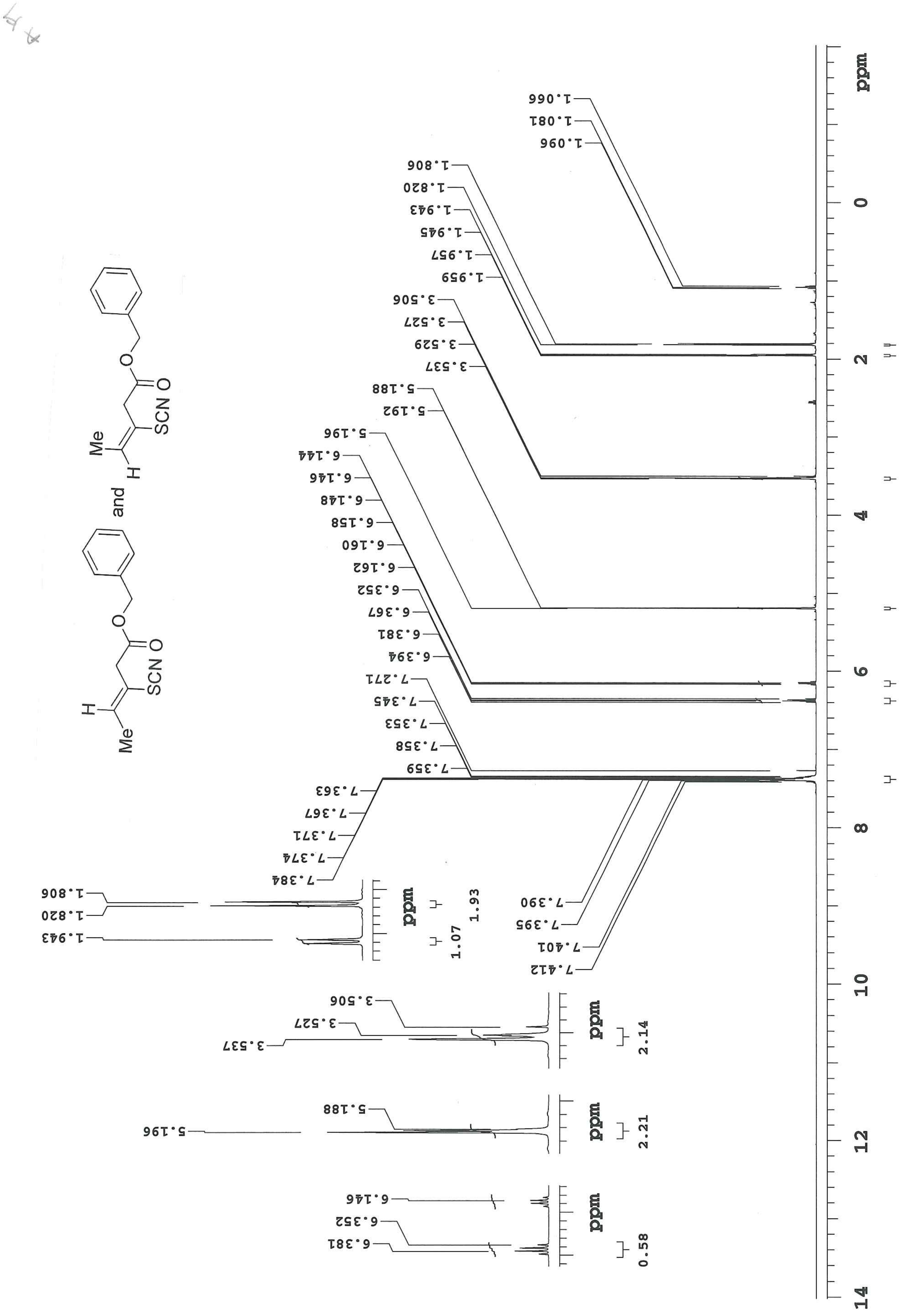

Me
H
SCN
O
and
ppm

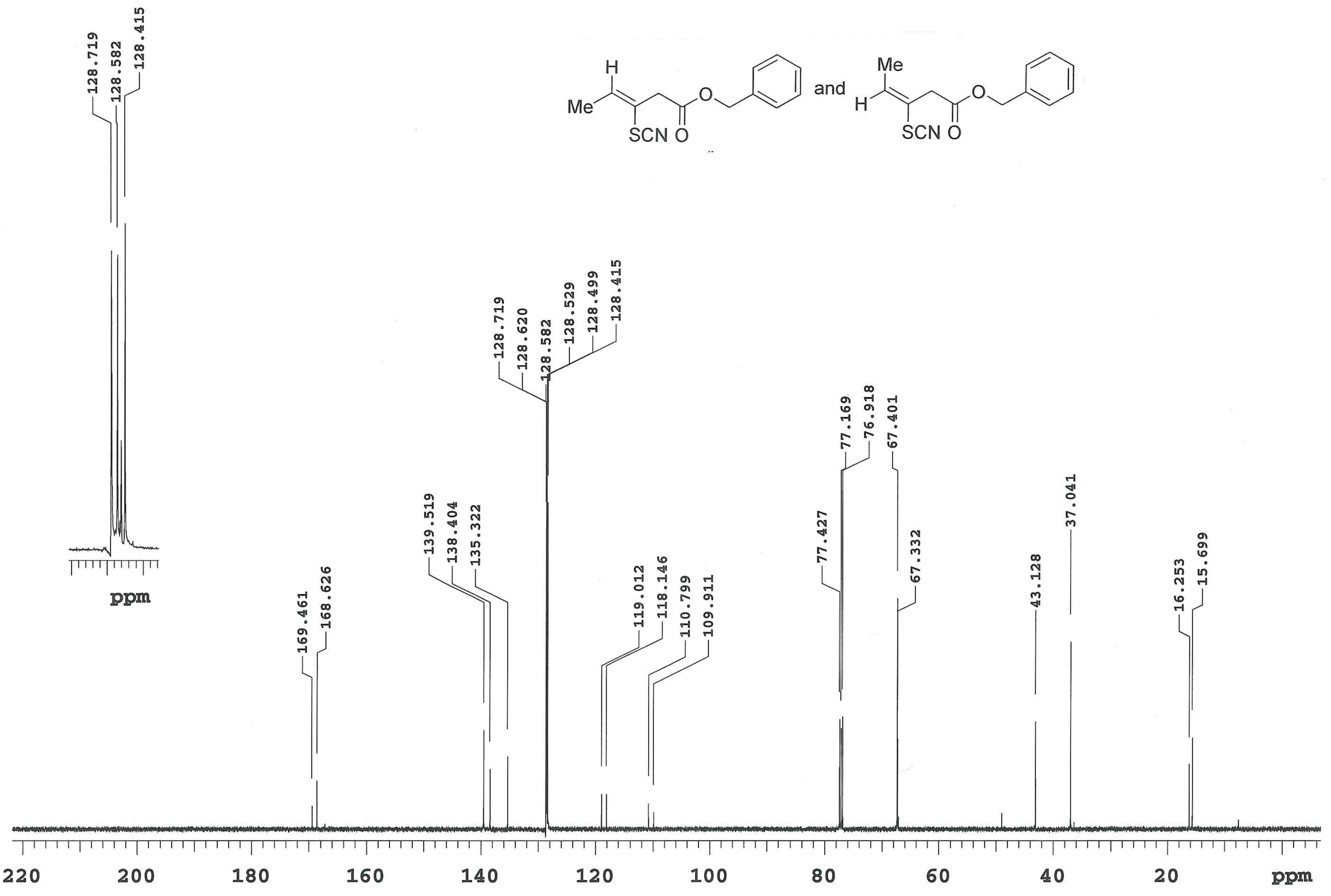

Me
H
SCN O
and
Me
H
SCN O
128.719
128.582
128.415
ppm
169.461
168.626
139.519
138.404
135.322
128.719
128.620
128.582
128.529
128.499
128.415
119.012
118.146
110.799
109.911
77.427
77.169
76.918
67.401
67.332
43.128
37.041
16.253
15.699
220
200
180
160
140
120
100
80
60
40
20
ppm

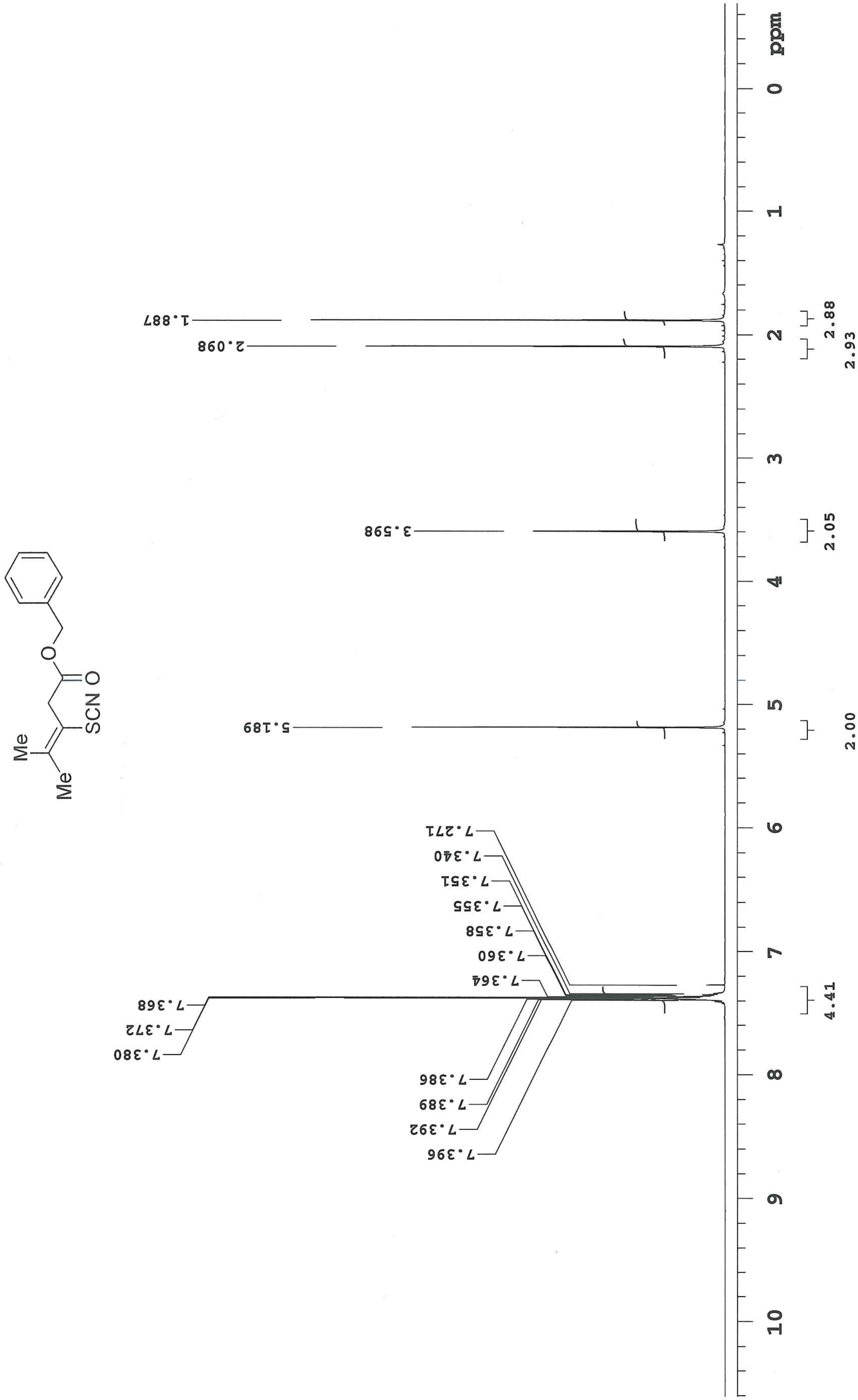
Me
Me
SCN
O
O
1.887
2.098
3.598
5.189
7.271
7.340
7.351
7.355
7.358
7.360
7.364
7.368
7.372
7.380
7.386
7.389
7.392
7.396
2.88
2.93
2.05
2.00
4.41
ppm
0
1
2
3
4
5
6
7
8
9
10

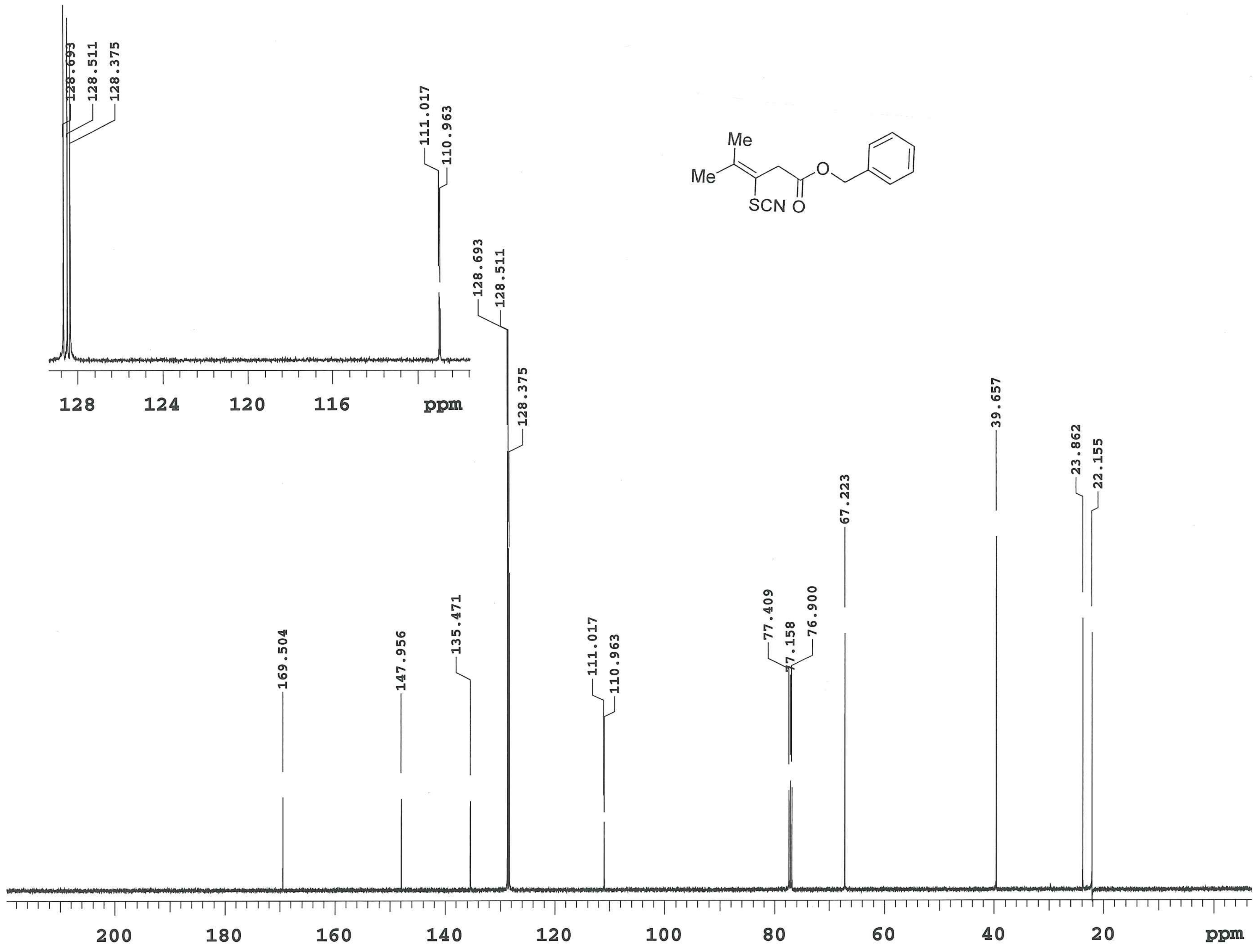
Me
Me
SCN
O
O
128.693
128.511
128.375
111.017
110.963
128
124
120
116
ppm
169.504
147.956
135.471
128.693
128.511
128.375
111.017
110.963
77.409
77.158
76.900
67.223
39.657
23.862
22.155
200
180
160
140
120
100
80
60
40
20
ppm

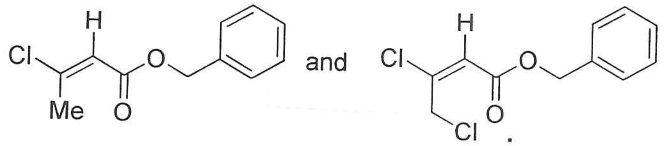
H
Cl
Me
O
O
and
H
Cl
O
O
Cl

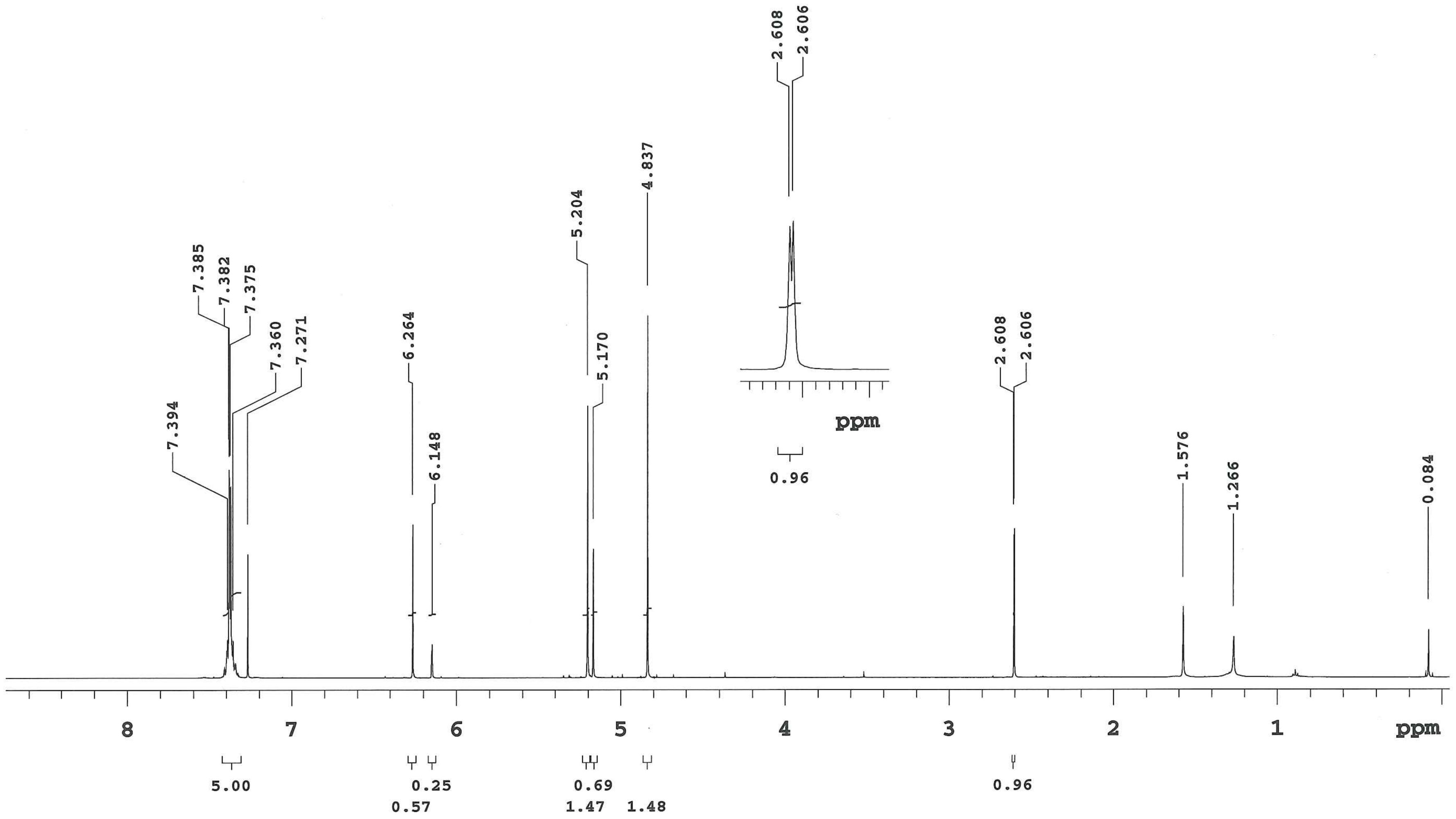
7.394
7.385
7.382
7.375
7.360
7.271
6.264
6.148
5.204
5.170
4.837
2.608
2.606
ppm
0.96
2.608
2.606
1.576
1.266
0.084
8
7
6
5
4
3
2
1
ppm
5.00
0.25
0.57
0.69
1.47
1.48
0.96

A21

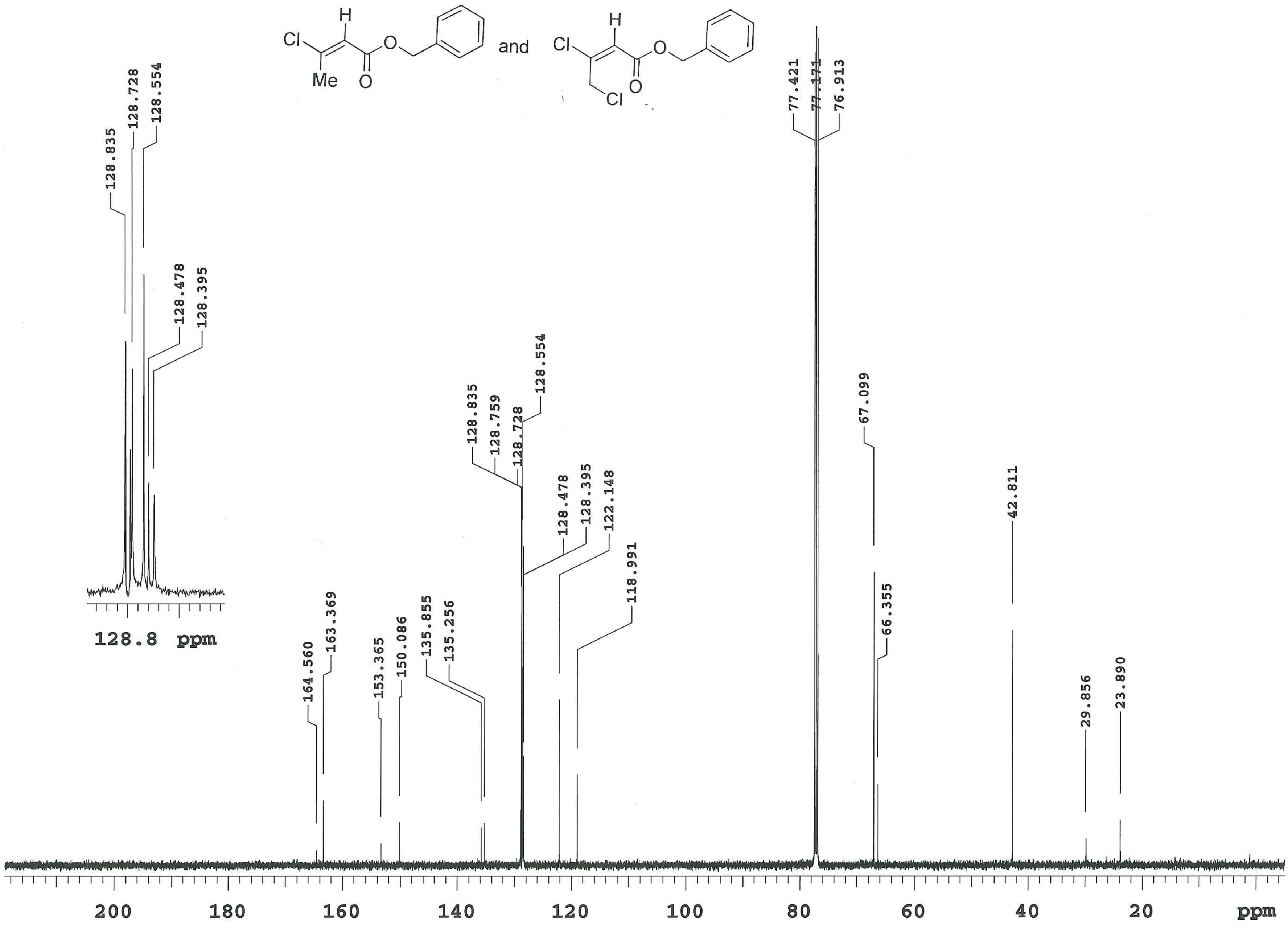
and
Cl
H
Me
O
128.835
128.728
128.554
128.478
128.395
128.8 ppm
164.560
163.369
153.365
150.086
135.855
135.256
128.835
128.759
128.728
128.554
128.478
128.395
122.148
118.991
77.421
77.171
76.913
67.099
66.355
42.811
29.856
23.890
200
180
160
140
120
100
80
60
40
20
ppm

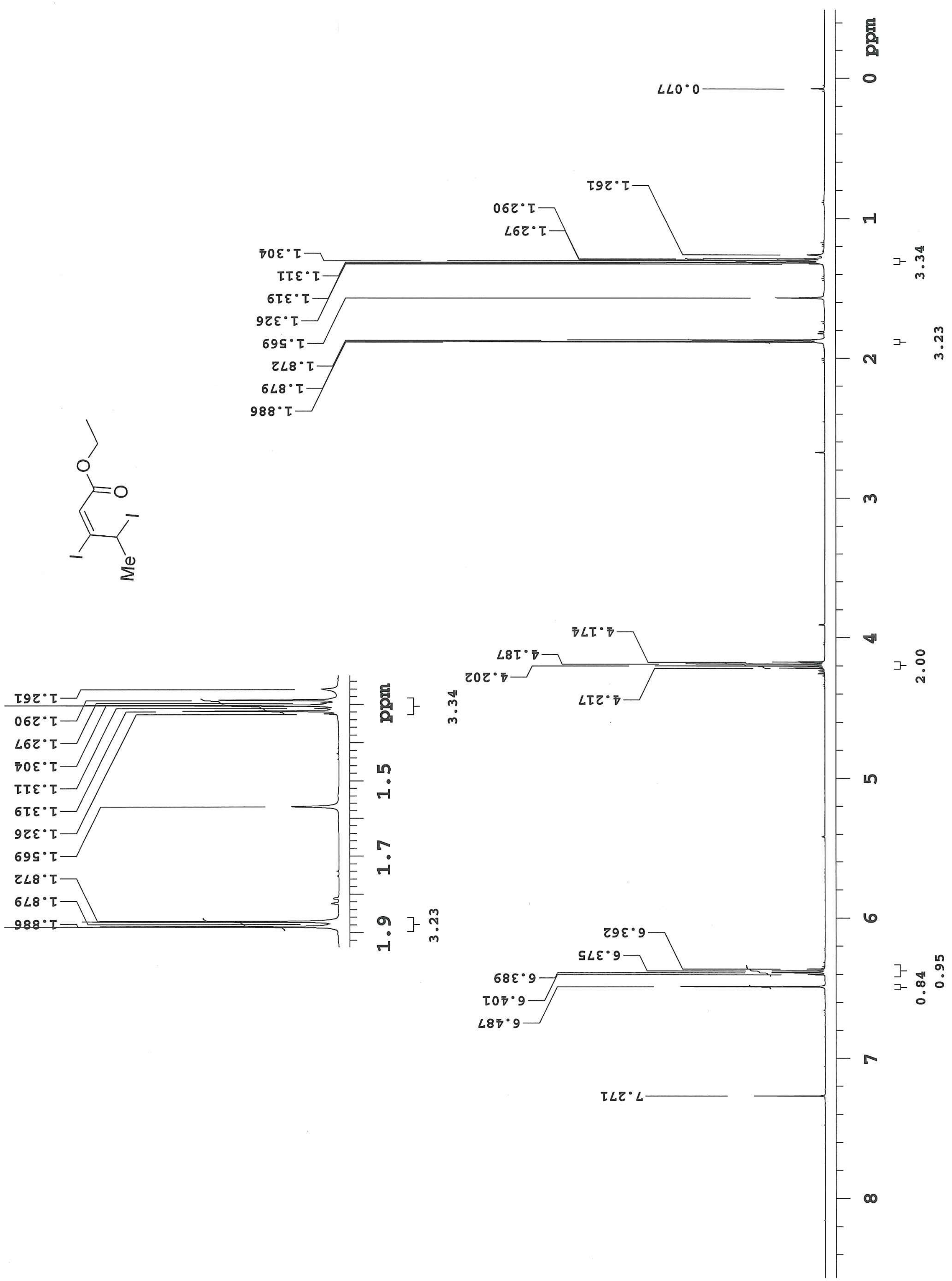
7.271
6.487
6.401
6.389
6.375
6.362
4.217
4.202
4.187
4.174
1.886
1.879
1.872
1.569
1.326
1.319
1.311
1.304
1.297
1.290
1.261
0.077
0.84
0.95
2.00
3.23
3.34
ppm
Me
I
O

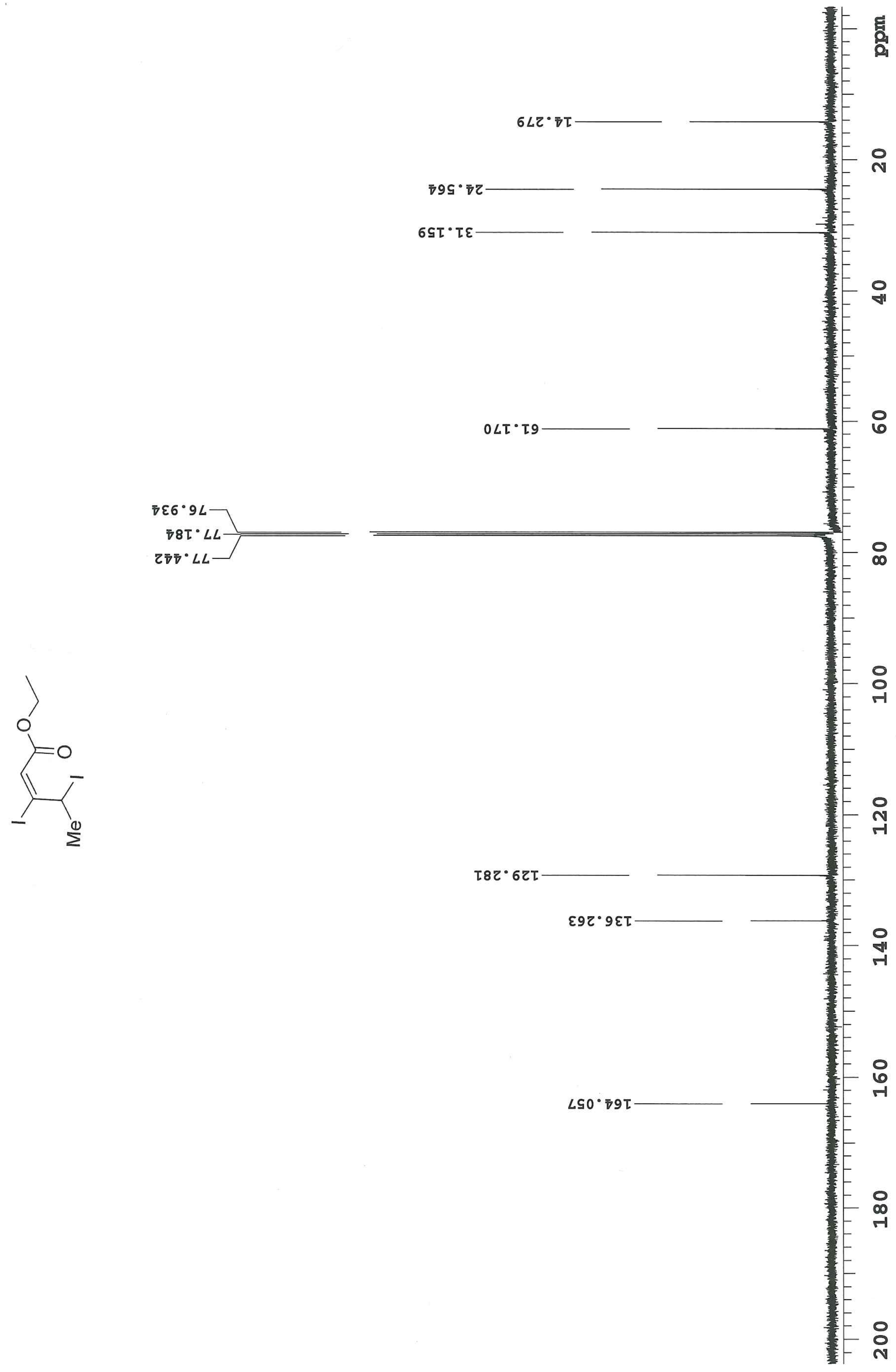
14.279
24.564
31.159
61.170
76.934
77.184
77.442
129.281
136.263
164.057
ppm
20
40
60
80
100
120
140
160
180
200
O
O
I
I
Me

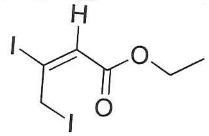
H
I
O
O
I

and

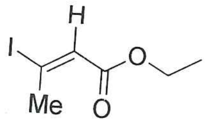
H
I
O
O
Me

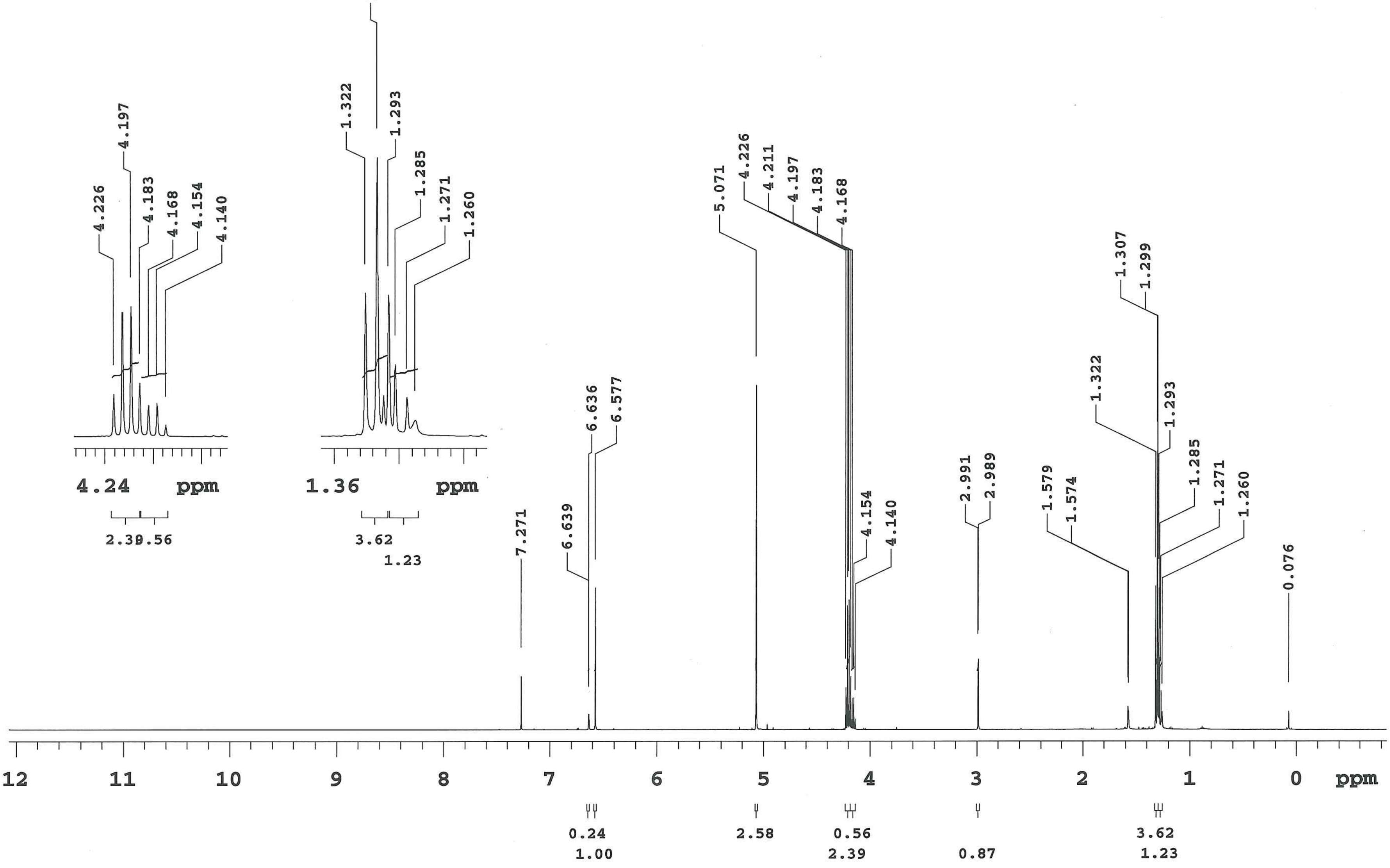
4.226
4.197
4.183
4.168
4.154
4.140
4.24 ppm
2.39
0.56
1.322
1.307
1.293
1.285
1.271
1.260
1.36 ppm
3.62
1.23
7.271
6.639
6.636
6.577
5.071
4.226
4.211
4.197
4.183
4.168
4.154
4.140
2.991
2.989
1.579
1.574
1.322
1.307
1.299
1.293
1.285
1.271
1.260
0.076
12
11
10
9
8
7
6
5
4
3
2
1
0
ppm
0.24
1.00
2.58
0.56
2.39
0.87
3.62
1.23

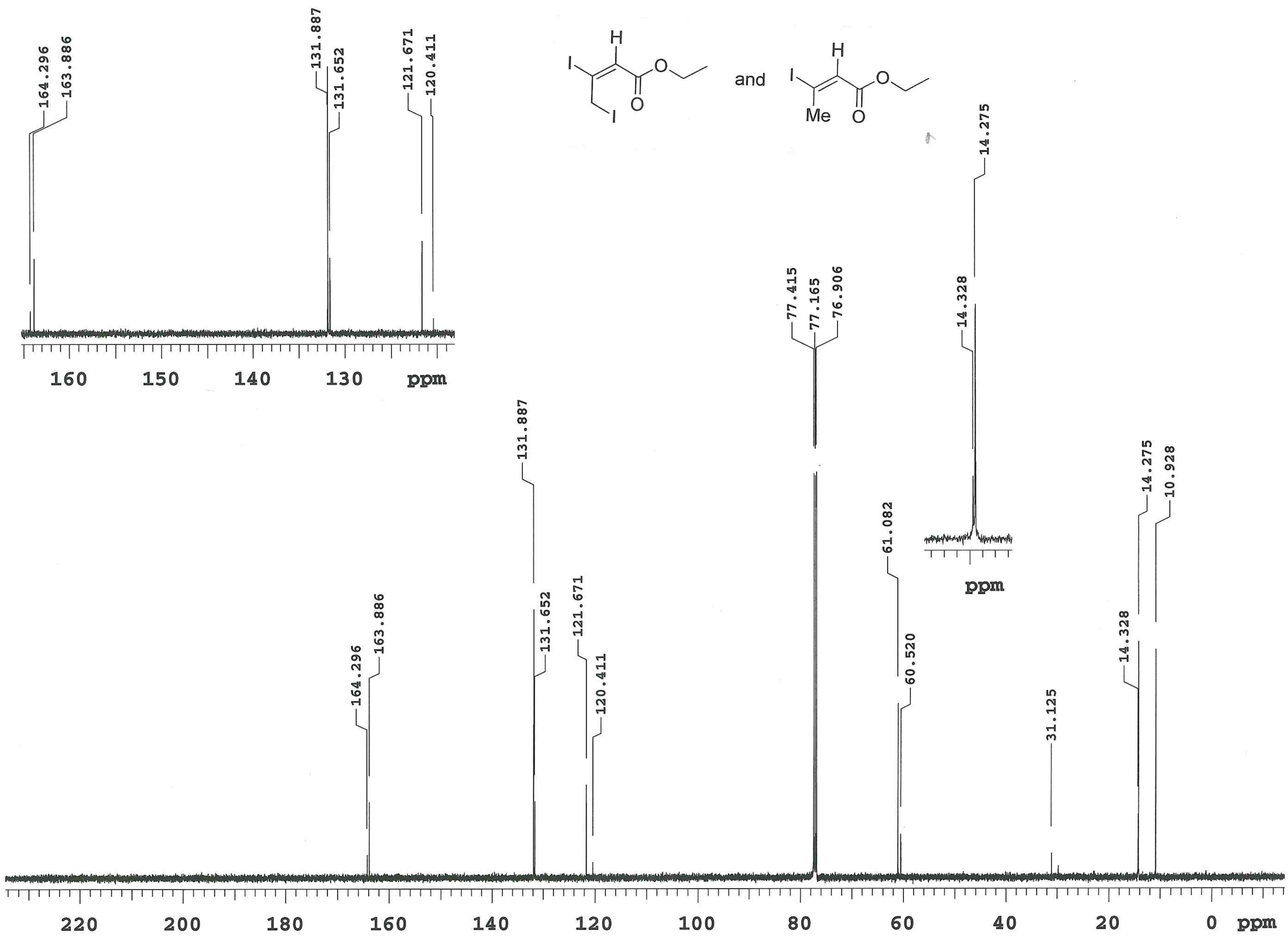

164.296
163.886
131.887
131.652
121.671
120.411
160
150
140
130
ppm
and
Me
14.275
14.328
77.415
77.165
76.906
61.082
60.520
31.125
14.328
14.275
10.928
220
200
180
160
140
120
100
80
60
40
20
0
ppm

A 23

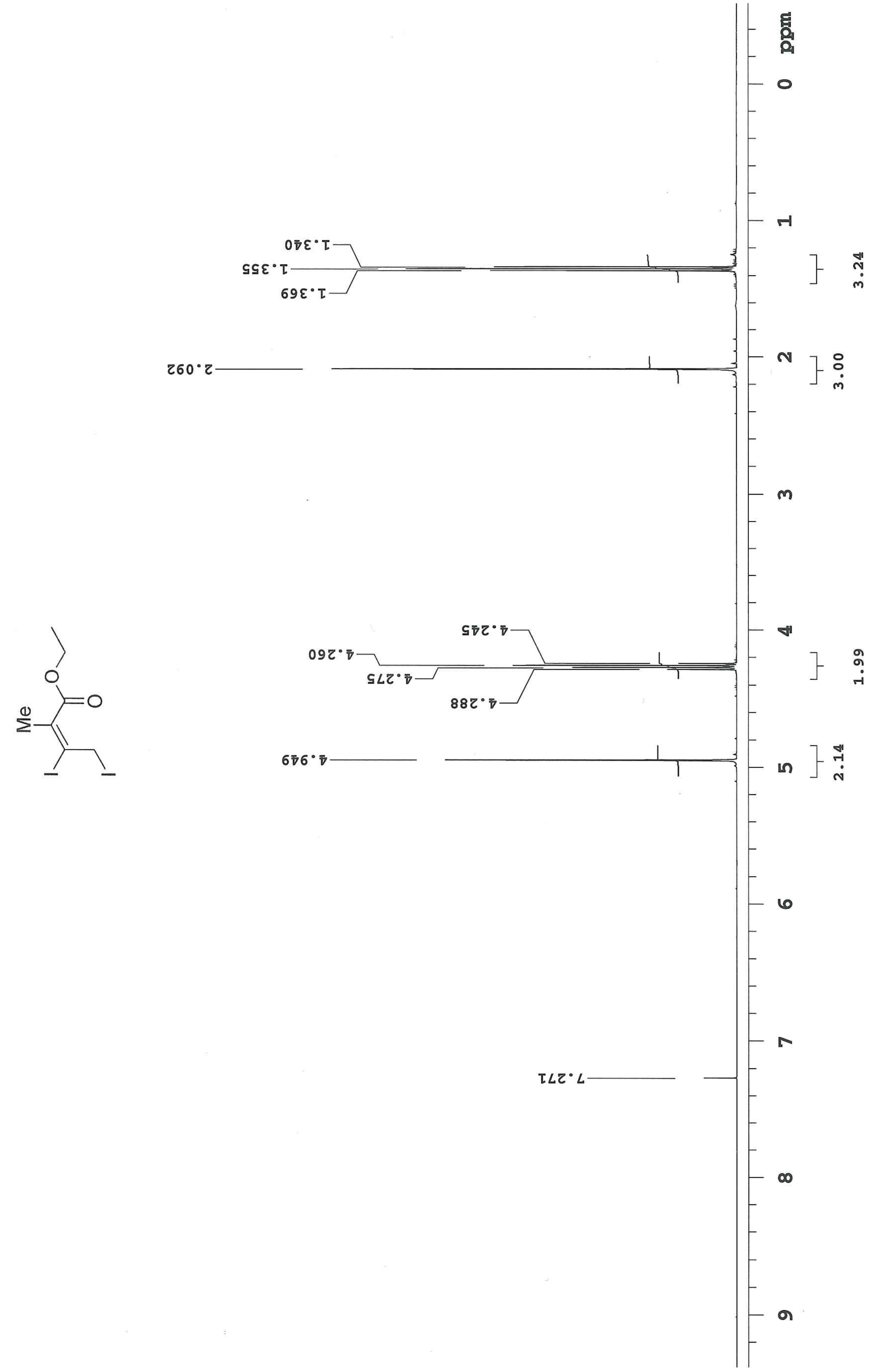

1.340
1.355
1.369
2.092
4.245
4.260
4.275
4.288
4.949
7.271
3.24
3.00
1.99
2.14
ppm
Me
O
I

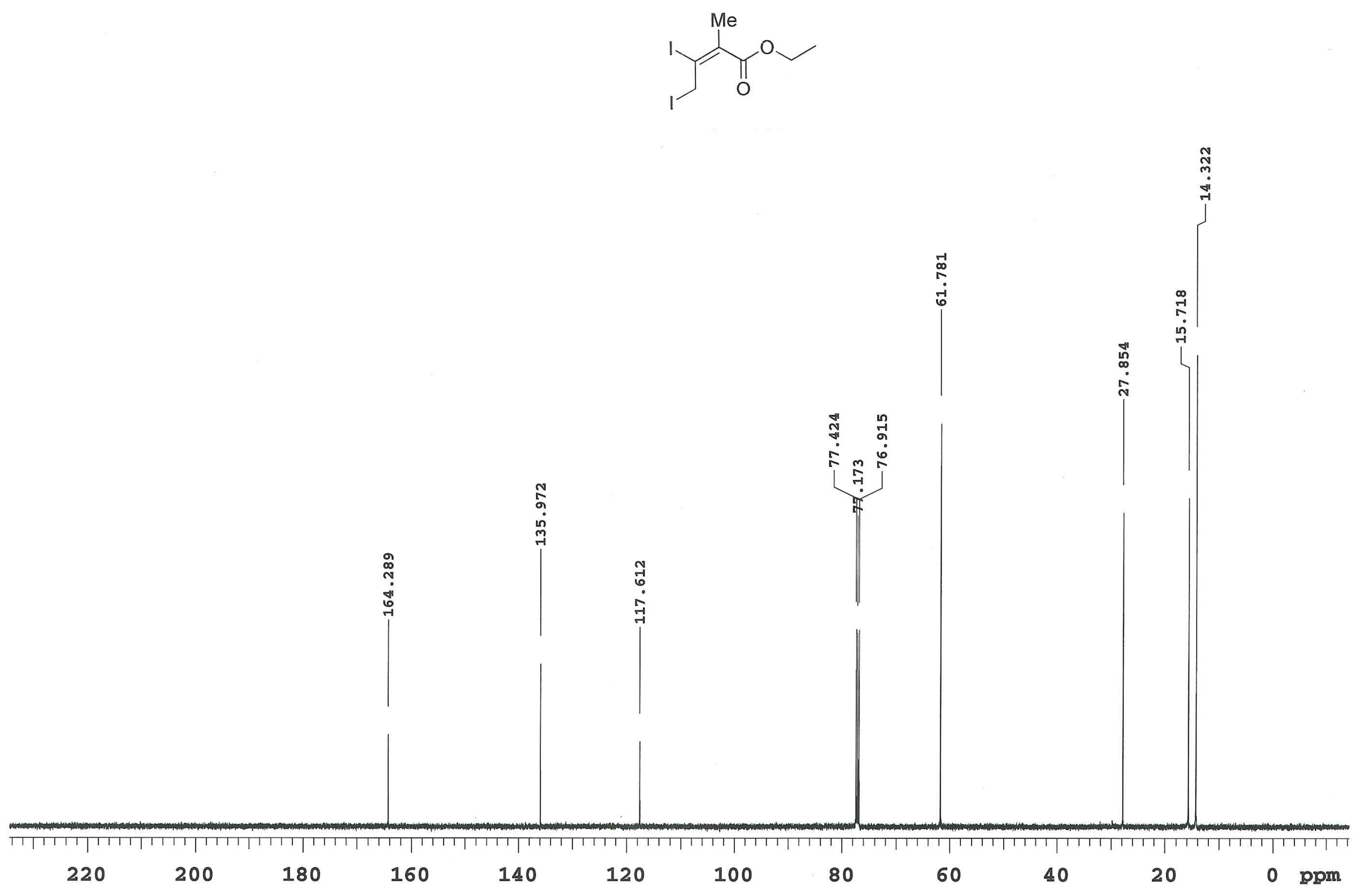
Me
I
O
O
I
164.289
135.972
117.612
77.424
77.173
76.915
61.781
27.854
15.718
14.322
220
200
180
160
140
120
100
80
60
40
20
0
ppm
